# Interpretable machine learning models based on multi-dimensional fusion data for predicting positive surgical margins in robot-assisted radical prostatectomy: a retrospective study

**DOI:** 10.3389/fonc.2025.1661695

**Published:** 2025-10-03

**Authors:** Zhangcheng Liu, Wenjun Zhou, Pan Dong, Jingyan Liu, Li Luo, Yu Luo, Shuai Su, Santigie Junior Sankoh, Yong Wang, Linhai Liu, Yang Zhang, Shilin Qiu, Lincen Jiang, Kun Han, Jindong Zhang, Jiang He, Delin Wang

**Affiliations:** ^1^ Department of Urology, The First Affiliated Hospital of Chongqing Medical University, Chongqing, China; ^2^ Department of Urology, The Second People’s Hospital of Neijiang, Neijiang, Sichuan, China; ^3^ Department of Urology, Guangan People's Hospital, Guangan, Sichuan, China; ^4^ Department of Radiology, The Second People’s Hospital of Neijiang, Neijiang, Sichuan, China; ^5^ Department of Respiratory and Critical Care Medicine, The First People’s Hospital of Neijiang, Neijiang, Sichuan, China; ^6^ Department of Medical Informatics Library, Chongqing Medical University, Chongqing, China; ^7^ Department of Urology, The University-Town Hospital of Chongqing Medical University, Chongqing, China

**Keywords:** prostate cancer, robot-assisted radical prostatectomy (RARP), multi-dimensional fusion data, multiparametric magnetic resonance imaging (mpMRI), machine learning, interpretation

## Abstract

**Objective:**

This study aimed to develop and validate interpretable machine learning (ML) models based on multi-dimensional fusion data for predicting positive surgical margins (PSM) in robot-assisted radical prostatectomy (RARP).

**Methods:**

Patients who underwent RARP at our institution between January 2016 and July 2025 were enrolled. Demographic, clinical, biopsy pathology data, and MRI-derived anatomical features (measured using ITK-SNAP on axial, sagittal, and coronal planes) were collected. Feature selection was performed using intraobserver and interobserver correlation coefficients (ICCs), low-variance filtering, univariable logistic regression, Spearman’s correlation analysis, the least absolute shrinkage and selection operator (LASSO) algorithm, and the Boruta algorithm. Six ML models were constructed, with performance evaluated using area under the curve (AUC), calibration curves, and decision curve analyses (DCA) to identify the optimal model. Five-fold and ten-fold cross-validation were used to assess the optimal model’s generalizability, and its interpretability was evaluated via Shapley Additive exPlanations (SHAP) analysis.

**Results:**

A total of 347 patients were included, comprising a training set (n=193, January 2016–December 2024), validation set (n=84, January 2016–December 2024), and test set (n=70, January 2025–July 2025). From 164 initial features, 7 key features were retained through a four-step screening. The Random Forest (RF) model outperformed other models, achieving AUCs of 0.99 (95% CI: 0.97–1.00) in the training set, 0.88 (95% CI: 0.80–0.95) in the validation set, and 0.97 (95% CI: 0.94–1.00) in the test set. Calibration curve and decision curve analyses confirmed its strong clinical utility. Five-fold cross-validation for the RF model showed fold-specific AUCs of 0.82–0.92, with a mean AUC of 0.87 (95% CI: 0.84–0.90). Ten-fold cross-validation showed fold-specific AUCs of 0.80–0.99, with a mean AUC of 0.88 (95% CI: 0.83–0.93). SHAP analysis revealed five novel spatial anatomical features (such as Sagittal plane-posterior spatial anatomical structure index, Coronal plane-Left anatomical structure interval) were negatively associated with PSM risk, while the number of positive biopsy cores and clinical tumor stage were positively associations.

**Conclusions:**

Multi-dimensional fusion data combined with ML models improves PSM prediction accuracy in RARP. The RF model, with excellent performance and interpretability, shows promise for preoperative PSM risk stratification, facilitates optimized clinical decision-making, and supports personalized treatment discussions during preoperative planning, but requires prospective and external validation before clinical implementation.

## Introduction

Prostate cancer (PCa) is one of the most common malignancies in men worldwide and ranks fifth among cancer-related deaths in males (1, 2). The “Cancer Statistics 2024” report estimates 299,010 new PCa cases and 35,250 related deaths in 2024 (2). Robot-assisted radical prostatectomy (RARP) is the primary surgical treatment for localized PCa and has become the gold standard for radical prostatectomy (RP) (3, 4), significantly improving overall and tumor-specific survival rates (5). By 2013, up to 80% of RPs in the United States were RARP procedures (6).

Positive surgical margin (PSM) in the prostate specimen following RP is a well-established predictor of biochemical recurrence (BCR) (7, 8). The incidence of PSM is influenced by multiple factors, including preoperative prostate-specific antigen (PSA) levels, clinical tumor stage (cT stage), Gleason score/International Society of Urological Pathology (ISUP) grade group, pathological extension of the primary tumor, and others (9–15). Patients with PSM face higher risks of BCR, disease progression, additional treatments, and psychological distress, which negatively impact quality of life (16–18). Given the diversity of RARP patients, preoperative prediction of surgical complexity and prognostic factors is critical for ensuring safety, optimizing scheduling, enhancing care, and reducing costs (19, 20).

Due to the prostate’s deep location within the pelvic cavity, RARP presents challenges such as limited surgical spatial related to prostate size and pelvic anatomy (21, 22). Recent studies have proposed pelvic measurement indicators to characterize pelvic anatomy (21, 23–26) and demonstrated that artificial intelligence (AI) models based on pelvic-prostate spatial features can predict RP surgical difficulty (22, 27–29). However, the stability of AI models depends on the quantity and quality of the training set, and existing models lack integration of radiomics, clinical, and biopsy pathology features, limiting their generalizability.

This study aimed to establish and validate a comprehensive machine learning (ML) algorithm integrating multi-dimensional fusion data (radiomics, prostate/pelvic measurements, clinical, and biopsy pathology features) for preoperative PSM prediction in RARP.

## Materials and methods

### Study cohorts

This retrospective single-center study was conducted at the Department of Urology, The First Affiliated Hospital of Chongqing Medical University, with collaborative support from co-authors at other institutions for data analysis and imaging feature quantification. This study was approved by the Institutional Review Board (IRB) of our hospital (Approval No. K2023-599) (**Supplementary Material 1**). As a retrospective study, informed consent from patients was waived. All study protocols were in accordance with the Declaration of Helsinki (30). Clinical data (demographic and laboratory variables), mpMRI data (anatomical features and relevant parameters), and biopsy pathological data were anonymized prior to analysis. PSM was defined as tumor cells at the inked surgical margin, regardless of anatomical location.

#### Training and validation sets

Patients who underwent RARP between January 2016 and December 2024 were enrolled. Exclusion criteria: (1) Missing or poor-quality mpMRI (n=279); (2) Incomplete biopsy pathology data (n=271); (3) Prior PCa treatment (androgen deprivation therapy, radiotherapy and others; n=200); (4) Non-puncture biopsy pathology diagnosis (such as transurethral resection of the prostate (TURP) and light laser vaporization, which may cause edema of the surrounding tissues) (n=70). (5) mpMRI performed after biopsy (n=42); (6) Missing laboratory data (n=17); (7) Other treated malignancies (n=13); (8) Distant metastases (n=4); (9) mpMRI performed more than 5 months before RARP (n=2); (10) Prostatic leiomyosarcoma (n=1); (11) TURP within 1 year (n=1).

#### Test set

**Figure 1:**
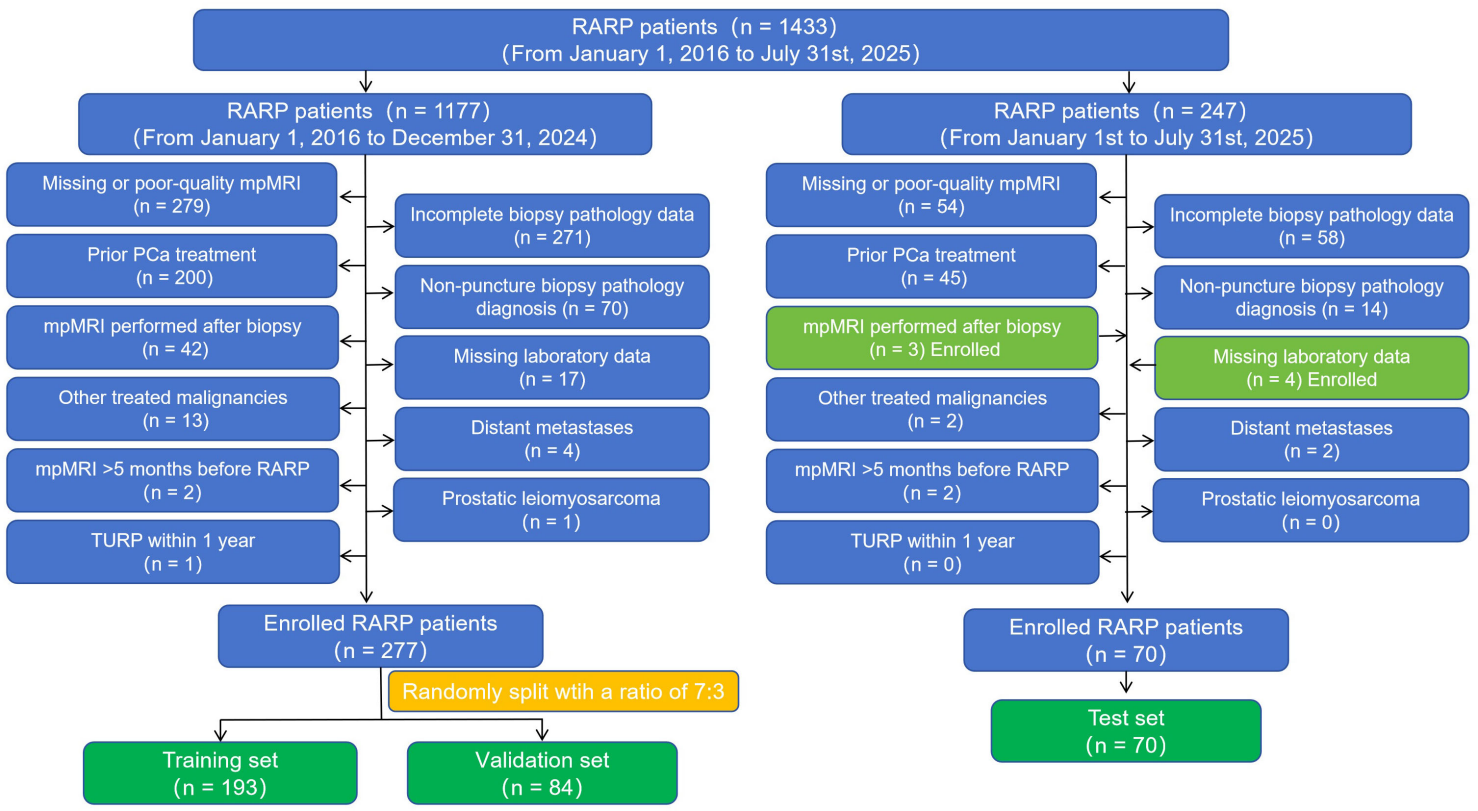
Screening flowchart of patients in this study (January 1, 2016, to July 31, 2025). RARP, Robot-Assisted Radical Prostatectomy; mpMRI, Multiparametric Magnetic Resonance Imaging; PCa, Prostate Cancer.

Patients who underwent RARP between January 2025 and July 2025 were enrolled. Exclusion criteria: (1) Missing or poor-quality mpMRI (n=54); (2) Incomplete biopsy pathology data (n=58); (3) Prior PCa treatment (n=45); (4) Non-puncture biopsy pathology diagnosis (n=14); (5) Other treated malignancies (n=2); (6) Distant metastases (n=2); (7) mpMRI performed more than 5 months before RARP (n=2); (8) prostatic leiomyosarcoma (n=0); (9) TURP within 1 year (n=0). The patient screening flowchart is shown in **Figure 1**.

### Clinical, mpMRI, and biopsy pathology data collection

#### Clinical data

A clinician blinded to mpMRI and pathology data extracted 49 features from electronic records, including demographics (age, BMI), lifestyle factors (smoking status, alcohol consumption), comorbidities (hypertension, diabetes), surgical details, laboratory tests (complete blood count, biochemical function, coagulation), and PCa markers (total prostate-specific antigen (tPSA), free prostate-specific antigen (fPSA), fPSA/PSA ratio).

#### mpMRI data

Imaging was performed using a 3.0 T MR scanner (GE Discovery MR750W, General Healthcare, Milwaukee, USA) with T2-weighted sequences (TR = 3,500 ms, TE = 85 ms, slice thickness=3 mm) and diffusion-weighted imaging (b-values=0, 1,000 s/mm²). Measurements were manually performed by two radiologists (Readers A and B, >8 years of PCa diagnosis experience) using ITK-SNAP (http://www.itksnap.org/). They were blinded to clinicopathological data and assessed Prostate Imaging-Reporting and Data System (PI-RADS) scores, clinical tumour stage, and measured pelvic, prostate, and tumor features. Intraobserver and interobserver correlation coefficients (ICCs) for MRI features ranged from 0.70 to 0.99, indicating good agreement (**Supplementary Table 1**). Controversial cases were re-evaluated by a senior radiologist (>15 years of experience).

Radiomics features (10 items): PI-RADS score, seminal vesicle invasion (SVI), lymph-node invasion (LNI), lympho-vascular invasion (LVI), perineural invasion, and others.

Anatomical measurements (97 items, **Supplementary Table 2**, **Supplementary Figure 1**):

- Axial plane (26 items): thickness of right obturator internus muscle (TROIM), thickness of left obturator internus muscle (TLOIM), distance of outer of the levator ani muscle (DOLAM), and others.
- Sagittal plane (31 items): prostatic urethral length (PUL), membranous urethral length (MUL), membranous urethral angle (MUA), and others.
- Coronal plane (12 items): right anal sphincter thickness (RST), left anal sphincter thickness (LST), thickness of right levator ani muscle (TRLAM), and others.
- Calculated values (28 items): thickness of levator ani muscle (TLAM), prostate-muscle index (PMI), roundness ratio (RR), and others.

#### Biopsy pathology evaluation

All patients underwent transrectal ultrasound-guided prostate biopsy by a single urologist. Biopsy pathology was reviewed by a senior pathologist (>10 years of PCa experience), blinded to MRI and postoperative pathology. Tumor classification was based on the 2016 WHO criteria, with grading via Gleason score and cancer group grades (31, 32). Eight features were recorded, including biopsy method, number of positive biopsy cores (PBC), Percentage of PBC, and others.

### Feature extraction and selection

Features with missing rates <10% were included. For imputation, continuous variables were filled with median values, and categorical variables with mode values to ensure comparability.

A four-step selection process was used: (1) Removal of low-variance features (baseline analysis). (2) Initial screening using univariable logistic regression. (3) Remove redundancy using Spearman’s rank correlation analysis (r ≥ 0.7). (4) Select optimal subsets via LASSO and Boruta algorithms, with the final features being the intersection of both.

### Hyperparameter tuning

To optimize each algorithm’s performance, we conducted hyperparameter tuning (33). This modeling process utilized a Bayesian hyperparameter search method (34), which systematically evaluated a comprehensive set of hyperparameter values to identify configurations maximizing efficiency and accuracy. Through this detailed and iterative exploration of the hyperparameter space, we were able to fine-tune the models effectively. This meticulous adjustment ensures that our models are precisely calibrated, significantly enhancing their ability to analyze and predict outcomes accurately with the dataset at hand.

### ML model construction, validation, and testing

Six ML models for predicting PSM were built: Logistic Regression (LR), Support Vector Machine (SVM), K-nearest Neighbor (KNN), Decision Tree (DT), Random Forest (RF), and Extreme Gradient Boosting (XGBoost). The receiver operating characteristic (ROC) curve analysis, area under the ROC curve (AUC), accuracy (ACC), sensitivity (SEN), specificity (SPE), positive predictive value (PPV), negative predictive value (NPV), and F1 score were calculated to evaluate model performance. To compare the predictive performance and clinical utility of the constructed ML models, the DeLong test, calibration curve analysis with Brier score loss, and decision curve analysis were conducted. A lower Brier score indicated superior model calibration.

### Cross-validation of the target model

To further validate model robustness, five-fold and ten-fold cross-validation were performed for the optimal model. In N-fold cross-validation, the dataset is divided into N equal folds; the model is trained on N-1 folds and validated on the remaining fold in each iteration. This process is repeated N times, with the final performance metric derived by averaging results to ensure a robust assessment (35).

### Machine learning model interpretation

**Figure 2:**
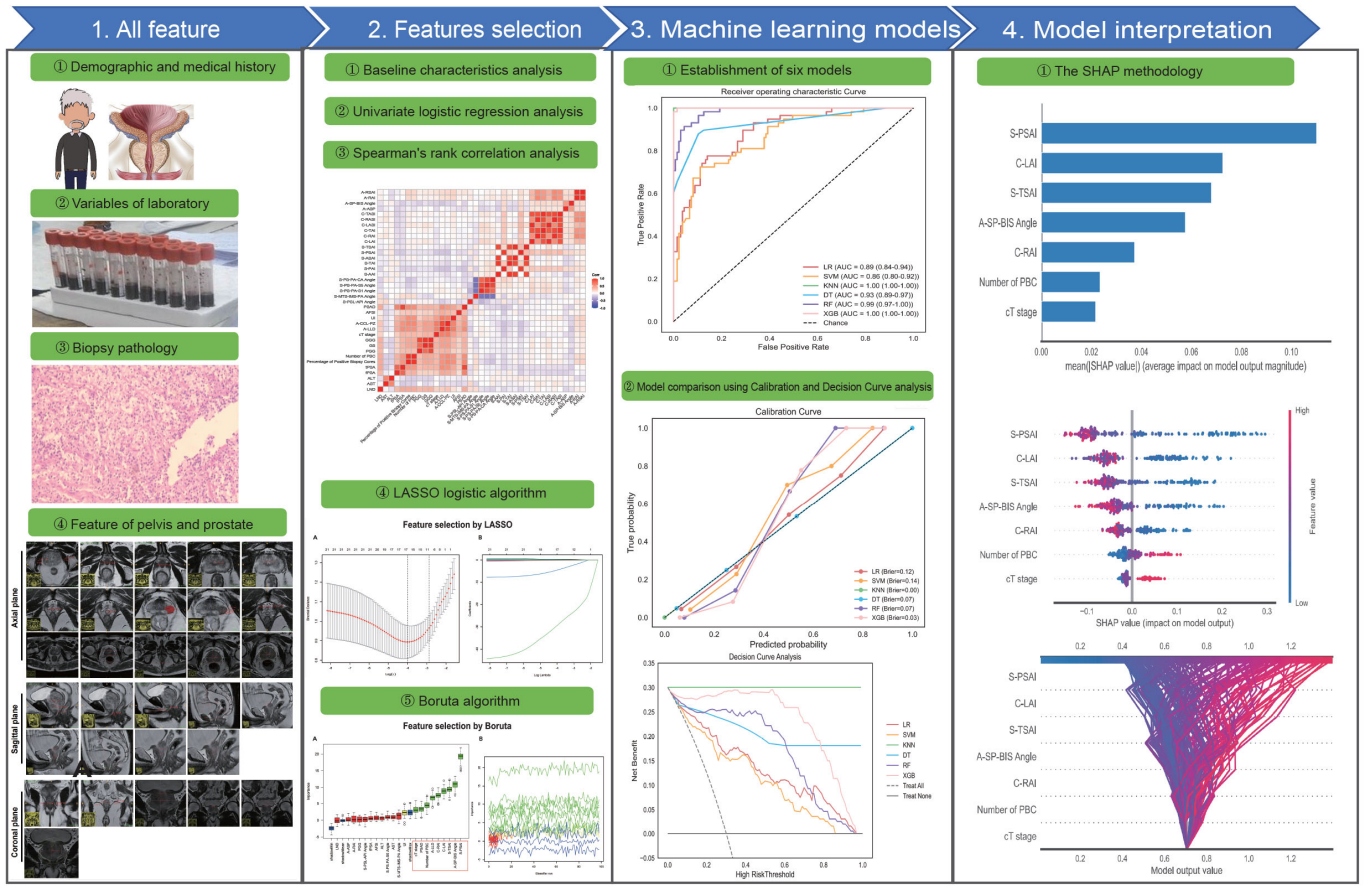
The overall workflow of this study. LASSO, Least Absolute Shrinkage and Selection Operator; SHAP, Shapley Additive exPlanations; ROC, Receiver Operating Characteristic Curve.

The optimal model was interpreted using SHAP (Shapley Additive exPlanations) analysis (36, 37). Based on cooperative game theory, SHAP quantifies each feature’s contribution to model predictions by evaluating its marginal impact across all feature combinations, ensuring a balanced representation of feature importance. It provides interpretability at two scales: (1) Local interpretability: clarifies individual predictions by quantifying feature contributions; (2) Global interpretability: synthesizes features’ relative impacts across the entire dataset. The workflow is illustrated in **Figure 2**.

### Statistical analysis

SPSS 25.0 (SPSS, Armonk, NY, USA), R software (version 4.3.1; https://www.r-project.org/), and Python (version 3.8.0; https://www.python.org/) were used for statistical analysis. Continuous variables were presented as medians with interquartile ranges (IQRs) and compared using Mann–Whitney U tests. Categorical data were presented as counts (percentages) and compared using chi-square, Fisher’s exact test, or Yates’ continuity correction. Accuracy, sensitivity, specificity, PPV, and NPV based on the optimal cutoff (Youden index) were calculated, with 95% confidence intervals (CIs) estimated using 1,000 bootstraps. A two-tailed *P*-value < 0.05 was considered statistically significant.

## Results

### Clinical characteristics

A total of 347 patients (median age: 70 years, IQR: 65.00-74.00 years) were included, with 238 (68.6%) negative surgical margins (NSM) and 109 (31.4%) PSM. No significant differences in clinical, mpMRI, or biopsy pathology features were observed between the training and validation sets (all P > 0.05; **Table 1**).

**Table 1:** Baseline characteristics (training vs. validation sets).

Characteristics	[ALL] N=277	[Training set] N=193	[Validation set] N=84	*P*-value
Positive surgical margin (PSM), n(% ) :				0.837
No	192 (69.3%)	135 (69.9%)	57 (67.9%)	
Yes	85 (30.7%)	58 (30.1%)	27 (32.1%)	
Demographic and medical history data:				
Age, median (IQR) (year):	69.0 [65.0;74.0]	70.0 [65.0;74.0]	68.5 [64.0;73.0]	0.32
Body mass index, median (IQR) (kg/m2):	24.2 [22.4;25.7]	23.9 [22.0;25.7]	24.5 [23.0;25.7]	0.145
Family history of PCa, n(% ) :				0.303
No	276 (99.6%)	193 (100%)	83 (98.8%)	
Yes	1 (0.36%)	0 (0.00%)	1 (1.19%)	
Abdominal surgery, n(% ) :				0.634
No	208 (75.1%)	147 (76.2%)	61 (72.6%)	
Yes	69 (24.9%)	46 (23.8%)	23 (27.4%)	
TURP, Over 1 year, n(% ) :				0.177
No	267 (96.4%)	188 (97.4%)	79 (94.0%)	
Yes	10 (3.61%)	5 (2.59%)	5 (5.95%)	
Smoking, n(% ) :				0.882
No	168 (60.6%)	116 (60.1%)	52 (61.9%)	
Yes	109 (39.4%)	77 (39.9%)	32 (38.1%)	
Drinking, n(% ) :				0.801
No	186 (67.1%)	131 (67.9%)	55 (65.5%)	
Yes	91 (32.9%)	62 (32.1%)	29 (34.5%)	
Hypertension, n(% ) :				1
No	160 (57.8%)	111 (57.5%)	49 (58.3%)	
Yes	117 (42.2%)	82 (42.5%)	35 (41.7%)	
Diabetes, n(% ) :				0.953
No	222 (80.1%)	154 (79.8%)	68 (81.0%)	
Yes	55 (19.9%)	39 (20.2%)	16 (19.0%)	
Cardiovascular disease, n(% ) :				0.359
No	234 (84.5%)	160 (82.9%)	74 (88.1%)	
Yes	43 (15.5%)	33 (17.1%)	10 (11.9%)	
Variables of laboratory data, Preoperative:				
Urinalysis white blood cell, median (IQR) (cell/ul):	2.00 [1.00;6.00]	2.00 [1.00;6.00]	2.00 [1.00;5.00]	0.972
Platelet, median (IQR) (109/L):	184 [156;212]	183 [157;210]	185 [155;217]	0.870
Hematocrit, median (IQR) (%):	42.5 [40.2;44.6]	42.6 [40.4;44.8]	42.0 [40.0;44.3]	0.157
Hemoglobin, median (IQR) (g/L):	141 [132;148]	140 [132;149]	141 [131;147]	0.519
White blood cell, median (IQR) (109/L):	5.68 [4.96;6.82]	5.68 [4.97;6.72]	5.84 [4.96;6.84]	0.602
Lymphocyte, median (IQR) (109/L):	1.61 [1.31;1.97]	1.60 [1.31;1.90]	1.65 [1.36;2.03]	0.461
Monocyte, median (IQR) (109/L):	0.47 [0.39;0.56]	0.47 [0.39;0.55]	0.46 [0.39;0.58]	0.603
Neutrophil, median (IQR) (109/L):	3.36 [2.69;4.26]	3.32 [2.62;4.22]	3.42 [2.73;4.32]	0.371
Neutrophil percentage, median (IQR) (%):	59.1 [53.4;64.1]	59.1 [53.0;64.0]	59.2 [54.9;64.9]	0.517
Neutrophil-to-lymphocyte ratio, median (IQR) :	2.06 [1.60;2.72]	2.06 [1.57;2.72]	2.07 [1.63;2.70]	0.753
Lymphocyte-to-monocyte ratio, median (IQR) :	3.50 [2.82;4.32]	3.52 [2.82;4.24]	3.48 [2.84;4.60]	0.905
Platelet-to-lymphocyte ratio, median (IQR) :	116 [90.1;139]	118 [90.9;139]	112 [85.2;138]	0.592
Monocyte-to-lymphocyte ratio, median (IQR) :	0.29 [0.23;0.35]	0.28 [0.24;0.35]	0.29 [0.22;0.35]	0.905
SII, median (IQR) :	372 [269;546]	370 [267;546]	377 [271;555]	0.619
Fasting blood glucose, median (IQR) (mmol/L):	5.40 [5.00;6.00]	5.40 [5.00;6.00]	5.40 [5.00;6.00]	0.655
Aspartate aminotransferas, median (IQR) (IU/L):	20.0 [17.0;24.0]	20.0 [16.0;25.0]	21.0 [17.0;24.0]	0.792
Alanine aminotransferase, median (IQR) (IU/L) :	19.0 [14.0;25.0]	18.0 [14.0;24.0]	19.5 [15.0;25.2]	0.300
DeRitis ratio, median (IQR) :	1.05 [0.85;1.29]	1.04 [0.85;1.31]	1.05 [0.85;1.25]	0.475
Blood urea nitrogen, median (IQR) (mmol/L) :	5.90 [4.90;7.00]	5.90 [5.00;7.00]	6.05 [4.70;7.12]	0.975
Serum creatinine, median (IQR) (umol/L):	81.0 [74.0;94.0]	81.0 [73.0;93.0]	83.5 [74.8;96.0]	0.191
eGFR, median (IQR) (ml/min/1.73m2):	88.4 [74.4;98.2]	88.9 [76.9;97.4]	87.5 [73.0;99.8]	0.545
Uric acid, median (IQR) (umol/L):	350 [302;399]	345 [296;400]	359 [312;398]	0.346
Prothrombin time, median (IQR) (s):	12.9 [12.3;13.5]	12.9 [12.3;13.4]	13.1 [12.5;13.6]	0.279
APTT, median (IQR) (s):	35.0 [32.4;37.5]	34.9 [32.5;37.8]	35.0 [32.4;37.3]	0.604
Fibrinogen, median (IQR) (g/L):	2.87 [2.55;3.23]	2.86 [2.57;3.25]	2.88 [2.47;3.20]	0.736
Thrombin time, median (IQR) (s):	17.6 [17.0;18.4]	17.7 [17.1;18.4]	17.4 [16.8;18.5]	0.122
International normalized ratio, median (IQR):	0.99 [0.95;1.04]	0.99 [0.94;1.04]	1.01 [0.96;1.05]	0.050
D-dimer, median (IQR) (mg/L):	0.34 [0.20;0.71]	0.34 [0.21;0.67]	0.34 [0.20;0.85]	0.851
fPSA, median (IQR) (ng/ml):	1.46 [0.93;2.54]	1.42 [0.91;2.48]	1.49 [0.94;2.71]	0.647
tPSA, median (IQR) (ng/ml):	13.6 [9.06;25.0]	13.6 [8.86;26.3]	13.8 [9.71;22.1]	0.943
fPSA/tPSA	0.10 [0.07;0.14]	0.10 [0.07;0.13]	0.11 [0.08;0.16]	0.074
Biopsy pathology:				
Biopsy Methods, n(% ):				0.091
Conventional	60 (21.7%)	47 (24.4%)	13 (15.5%)	
Systematic biopsy	157 (56.7%)	110 (57.0%)	47 (56.0%)	
MRI-ultrasound fusion-guided targeted biopsy	60 (21.7%)	36 (18.7%)	24 (28.6%)	
Number of biopsy cores, median (IQR):	12.0 [12.0;12.0]	12.0 [12.0;12.0]	12.0 [12.0;12.0]	0.407
Number of positive biopsy cores, median (IQR):	5.00 [3.00;7.00]	5.00 [3.00;7.00]	5.00 [2.75;7.00]	0.459
Percentage of PBC, median (IQR) (%):	41.7 [22.2;58.3]	41.7 [22.2;58.3]	41.4 [25.0;60.9]	0.646
Primary Gleason grade, n(% ):				0.409
3	150 (54.2%)	102 (52.8%)	48 (57.1%)	
4	118 (42.6%)	86 (44.6%)	32 (38.1%)	
5	9 (3.25%)	5 (2.59%)	4 (4.76%)	
Secondary Gleason grade, n(% ):				0.854
3	139 (50.2%)	99 (51.3%)	40 (47.6%)	
4	113 (40.8%)	77 (39.9%)	36 (42.9%)	
5	25 (9.03%)	17 (8.81%)	8 (9.52%)	
Gleason score, n(% ):				0.749
3+3	78 (28.2%)	55 (28.5%)	23 (27.4%)	
3+4, 4+3	128 (46.2%)	90 (46.6%)	38 (45.2%)	
3+5, 4+4, 5+3	43 (15.5%)	30 (15.5%)	13 (15.5%)	
4+5, 5+4	25 (9.03%)	15 (7.77%)	10 (11.9%)	
5+5	3 (1.08%)	3 (1.55%)	0 (0.00%)	
Gleason grade group, n(% ):				0.914
1	80 (28.9%)	55 (28.5%)	25 (29.8%)	
2	68 (24.5%)	47 (24.4%)	21 (25.0%)	
3	58 (20.9%)	43 (22.3%)	15 (17.9%)	
4	43 (15.5%)	30 (15.5%)	13 (15.5%)	
5	28 (10.1%)	18 (9.33%)	10 (11.9%)	
MRI data:				
PI-RADS v2, n(% ):				0.627
2	27 (9.75%)	20 (10.4%)	7 (8.33%)	
3	21 (7.58%)	14 (7.25%)	7 (8.33%)	
4	54 (19.5%)	34 (17.6%)	20 (23.8%)	
5	175 (63.2%)	125 (64.8%)	50 (59.5%)	
Lymph-node invasion:				0.587
No	273 (98.6%)	191 (99.0%)	82 (97.6%)	
Yes	4 (1.44%)	2 (1.04%)	2 (2.38%)	
Lympho-vascular invasion:				0.357
No	265 (95.7%)	183 (94.8%)	82 (97.6%)	
Yes	12 (4.33%)	10 (5.18%)	2 (2.38%)	
Perineural invasion:				0.892
No	258 (93.1%)	179 (92.7%)	79 (94.0%)	
Yes	19 (6.86%)	14 (7.25%)	5 (5.95%)	
Urethral invasion:				0.398
No	193 (69.7%)	131 (67.9%)	62 (73.8%)	
Yes	84 (30.3%)	62 (32.1%)	22 (26.2%)	
External urethral sphincter invasion:				0.521
No	265 (95.7%)	186 (96.4%)	79 (94.0%)	
Yes	12 (4.33%)	7 (3.63%)	5 (5.95%)	
Seminal vesicle invasion:				0.182
No	266 (96.0%)	183 (94.8%)	83 (98.8%)	
Yes	11 (3.97%)	10 (5.18%)	1 (1.19%)	
Rectal invasion:				1.000
No	276 (99.6%)	192 (99.5%)	84 (100%)	
Yes	1 (0.36%)	1 (0.52%)	0 (0.00%)	
Anterior Fibromuscular Stroma invasion:				0.291
No	192 (69.3%)	138 (71.5%)	54 (64.3%)	
Yes	85 (30.7%)	55 (28.5%)	30 (35.7%)	
Clinical primary tumor Stage (cT stage), n(%):				0.963
1	27 (9.75%)	19 (9.84%)	8 (9.52%)	
2	168 (60.6%)	118 (61.1%)	50 (59.5%)	
3	68 (24.5%)	47 (24.4%)	21 (25.0%)	
4	14 (5.05%)	9 (4.66%)	5 (5.95%)	
Axial plane				
A-TROIM, median (IQR) (mm):	19.5 [17.5;21.5]	19.6 [17.4;21.6]	19.2 [17.5;21.2]	0.570
A-TLOIM, median (IQR) (mm):	19.2 [17.2;21.1]	19.0 [17.2;20.8]	19.4 [17.3;21.5]	0.376
A-DOLAM, median (IQR) (mm):	40.8 [38.3;43.0]	40.7 [38.6;43.0]	40.8 [38.1;43.1]	0.615
A-DILAM, median (IQR) (mm):	15.2 [14.0;16.6]	15.2 [13.9;16.6]	15.2 [14.1;16.7]	0.894
A-UW, median (IQR) (mm):	1.28 [1.14;1.41]	1.27 [1.13;1.41]	1.31 [1.18;1.45]	0.361
A-UWT, median (IQR) (mm):	1.93 [1.72;2.29]	1.98 [1.76;2.29]	1.87 [1.63;2.33]	0.071
A-TMUT, median (IQR) (mm):	7.69 [6.76;8.63]	7.65 [6.69;8.67]	7.77 [6.84;8.56]	0.895
A-APMUT, median (IQR) (mm):	7.64 [6.84;8.59]	7.48 [6.74;8.57]	7.77 [7.04;8.67]	0.209
A-RLP, median (IQR) (mm):	4.93 [3.75;6.62]	4.86 [3.71;6.50]	5.12 [4.01;6.74]	0.247
A-LLP, median (IQR) (mm):	4.92 [3.78;6.53]	4.71 [3.68;6.58]	5.06 [4.01;6.45]	0.309
A-LLD, median (IQR) (mm):	18.1 [10.9;27.9]	18.7 [10.8;27.8]	16.8 [11.4;28.8]	0.683
A-CCL-PZ, median (IQR) (mm):	14.0 [0.00;33.9]	15.3 [0.00;34.9]	11.8 [0.00;32.1]	0.434
A-OID, median (IQR) (mm):	73.1 [65.4;81.6]	72.9 [65.4;81.3]	73.6 [65.7;82.4]	0.908
A-AAI, median (IQR) (mm):	9.84 [7.57;13.0]	9.80 [7.62;12.8]	9.91 [7.56;13.6]	0.803
A-ISD, median (IQR) (mm):	92.0 [88.1;96.2]	92.1 [88.1;97.5]	91.2 [88.2;94.8]	0.317
A-SW, median (IQR) (mm):	79.4 [68.2;86.3]	80.0 [68.9;86.0]	76.3 [66.9;87.2]	0.212
A-BFW, median (IQR) (mm):	95.2 [91.1;99.5]	95.2 [91.2;99.2]	95.2 [91.0;100]	0.829
A-ITD, median (IQR) (mm):	118 [112;125]	119 [113;125]	116 [111;123]	0.154
A-ASP, median (IQR) (°):	73.3 [68.8;77.5]	73.7 [69.3;77.5]	72.4 [68.2;77.9]	0.285
A-SP-BIS Angle, median (IQR) (°):	56.4 [52.8;60.1]	56.9 [52.9;60.6]	55.8 [52.8;58.6]	0.183
A-PTD, median (IQR) (mm):	49.0 [45.6;53.1]	48.9 [46.0;53.0]	49.2 [44.6;53.5]	0.924
A-PAD, median (IQR) (mm):	49.0 [45.6;53.2]	48.6 [45.8;53.0]	49.3 [44.6;53.8]	0.696
A-LAI, median (IQR) (mm):	5.24 [3.86;7.61]	5.18 [3.92;7.45]	5.54 [3.67;7.74]	0.903
A-RAI, median (IQR) (mm):	5.42 [3.63;7.65]	5.28 [3.47;7.26]	5.76 [3.87;8.02]	0.180
A-NTL, n(%):				0.874
0	40 (14.4%)	27 (14.0%)	13 (15.5%)	
1	179 (64.6%)	126 (65.3%)	53 (63.1%)	
2	45 (16.2%)	32 (16.6%)	13 (15.5%)	
≥3	13 (4.69%)	8 (4.15%)	5 (5.95%)	
A-TLI , n(%):				0.891
No	40 (14.4%)	27 (14.0%)	13 (15.5%)	
Yes	237 (85.6%)	166 (86.0%)	71 (84.5%)	
Sagittal plane				
S-PUL, median (IQR) (mm):	45.0 [41.4;49.6]	44.2 [41.3;49.3]	46.0 [41.8;51.2]	0.151
S-MUL, median (IQR) (mm):	15.0 [14.0;15.9]	15.0 [14.0;15.8]	15.2 [14.1;16.1]	0.306
S-MUA, median (IQR) (°):	122 [116;128]	122 [116;129]	121 [117;128]	0.568
S-LASP, median (IQR) (mm):	40.8 [38.3;43.6]	40.9 [38.8;43.7]	39.9 [37.6;43.1]	0.050
S-API, median (IQR) (mm):	110 [103;116]	110 [104;116]	110 [103;116]	0.571
S-APM, median (IQR) (mm):	107 [103;112]	108 [103;112]	106 [101;111]	0.167
S-APO, median (IQR) (mm):	86.9 [81.4;91.5]	87.1 [81.7;91.4]	85.5 [81.0;92.1]	0.728
S-PD, median (IQR) (mm):	124 [118;131]	124 [119;131]	124 [117;132]	0.544
S-SD, median (IQR) (mm):	33.2 [28.6;37.6]	33.6 [28.3;38.4]	32.1 [29.0;36.6]	0.208
S-S1AMCAL, median (IQR) (mm):	125 [118;133]	127 [118;133]	124 [117;132]	0.453
S-AVPJ, median (IQR) (mm):	16.0 [12.3;20.4]	16.8 [12.3;20.9]	14.9 [12.0;18.5]	0.033
S-AD, median (IQR) (mm):	33.4 [30.1;37.3]	33.4 [30.1;37.6]	33.4 [29.8;36.9]	0.688
S-BH, median (IQR) (mm):	12.4 [6.89;18.1]	12.1 [6.84;17.6]	12.7 [7.34;18.9]	0.345
S-IPPH, median (IQR) (mm):	0.00 [0.00;5.22]	1.23 [0.00;5.33]	0.00 [0.00;5.17]	0.405
S-UUP, median (IQR) (mm):	6.55 [2.66;11.1]	6.35 [2.52;10.4]	7.12 [2.88;13.3]	0.107
S-DUP, median (IQR) (mm):	30.6 [27.5;34.3]	30.6 [27.6;34.5]	30.3 [26.7;34.0]	0.491
S-SA, median (IQR) (°):	39.1 [35.9;42.4]	38.6 [35.1;42.3]	39.3 [37.2;42.9]	0.180
S-RMA, median (IQR) (°):	155 [146;163]	155 [145;163]	157 [148;163]	0.343
S-PIA, median (IQR) (°):	69.1 [66.0;72.7]	69.1 [66.2;72.8]	69.0 [66.0;72.4]	0.631
S-LASP-APO Angle, median (IQR) (°):	130 [126;135]	130 [126;135]	130 [126;134]	0.967
S-LASP-API Angle, median (IQR) (°):	101 [95.7;105]	100 [95.3;105]	101 [96.3;105]	0.479
S-LASP-PD Angle, median (IQR) (°):	59.4 [55.9;62.6]	59.9 [55.9;63.1]	58.8 [56.4;61.7]	0.474
S-APO-API Angle, median (IQR) (°):	51.2 [45.9;56.5]	51.2 [45.6;56.5]	51.3 [47.8;56.3]	0.581
S-MTSP-IMSPA Angle, median (IQR) (°):	130 [122;137]	131 [122;137]	128 [120;134]	0.053
S-SP-PA-S1 Angle, median (IQR) (°):	74.8 [67.8;81.3]	74.7 [67.5;80.9]	75.0 [69.7;82.6]	0.334
S-SP-PA-S5 Angle, median (IQR) (°):	131 [124;140]	131 [124;139]	132 [123;142]	0.595
S-SP-PA-CA Angle, median (IQR) (°):	148 [139;159]	149 [138;158]	146 [140;160]	0.914
S-PAD, median (IQR) (mm):	36.8 [33.0;40.8]	37.0 [33.0;41.3]	36.5 [33.2;40.4]	0.562
S-PCD, median (IQR) (mm):	45.3 [41.2;51.3]	45.2 [41.1;50.4]	45.6 [41.5;52.5]	0.433
S-AAI, median (IQR) (mm):	13.2 [10.3;16.4]	13.5 [10.8;16.6]	11.8 [9.32;16.1]	0.084
S-PAI, median (IQR) (mm):	2.51 [1.89;3.35]	2.51 [1.94;3.34]	2.50 [1.80;3.40]	0.973
Coronal plane				
C-RST, median (IQR) (mm):	7.31 [6.15;8.81]	7.38 [6.12;8.78]	7.30 [6.20;8.84]	0.763
C-LST, median (IQR) (mm):	7.39 [6.14;8.75]	7.39 [6.11;8.75]	7.39 [6.16;8.70]	0.895
C-TRLAM, median (IQR) (mm):	4.84 [4.03;5.42]	4.89 [4.06;5.42]	4.64 [4.00;5.38]	0.179
C-TLLAM, median (IQR) (mm):	4.74 [4.16;5.52]	4.81 [4.21;5.66]	4.64 [4.08;5.26]	0.104
C-TVPJ, median (IQR) (mm):	20.1 [15.3;25.2]	20.2 [15.5;25.6]	19.8 [15.1;24.4]	0.415
C-IPPH, median (IQR) (mm):	2.79 [0.00;6.24]	2.79 [0.00;6.49]	2.87 [0.00;6.07]	0.733
C-TIP, median (IQR) (mm):	111 [106;116]	111 [106;116]	110 [106;116]	0.667
C-TTP, median (IQR) (mm):	104 [100.0;108]	104 [101;108]	104 [99.8;106]	0.429
C-PTD, median (IQR) (mm):	49.3 [46.0;53.1]	49.3 [46.2;53.1]	49.4 [45.8;53.1]	0.735
C-PCD, median (IQR) (mm):	41.8 [37.6;47.7]	41.5 [37.4;47.5]	42.8 [38.1;48.6]	0.380
C-LAI, median (IQR) (mm):	4.32 [2.94;6.10]	4.22 [2.87;6.10]	4.36 [3.06;6.09]	0.495
C-RAI, median (IQR) (mm):	4.62 [3.17;6.36]	4.62 [3.06;6.21]	4.64 [3.26;6.84]	0.343
Calculated value				
A-TLAM, median (IQR) (mm):	12.6 [11.6;13.9]	12.6 [11.6;14.0]	12.5 [11.5;13.7]	0.642
A-PMI, median (IQR) (mm):	23.1 [17.8;31.2]	23.1 [17.6;30.9]	23.1 [17.9;32.2]	0.814
A-RR, median (IQR):	0.78 [0.72;0.85]	0.79 [0.72;0.85]	0.78 [0.72;0.84]	0.757
A-TAI, median (IQR) (mm):	10.7 [7.83;14.4]	10.6 [7.70;14.1]	10.8 [8.18;15.3]	0.366
A-LSAI, median (IQR):	0.11 [0.07;0.16]	0.11 [0.07;0.15]	0.11 [0.07;0.16]	0.846
A-RSAI, median (IQR):	0.11 [0.07;0.15]	0.11 [0.07;0.15]	0.12 [0.08;0.16]	0.186
A-TSAI, median (IQR):	0.22 [0.15;0.31]	0.22 [0.15;0.30]	0.24 [0.17;0.32]	0.358
S-RR, median (IQR):	0.80 [0.74;0.87]	0.81 [0.75;0.87]	0.78 [0.73;0.85]	0.057
S-TAI, median (IQR) (mm):	16.0 [12.8;19.3]	16.3 [13.3;19.3]	15.0 [11.6;19.2]	0.167
S-ASAI, median (IQR):	0.36 [0.27;0.46]	0.37 [0.28;0.47]	0.32 [0.25;0.41]	0.071
S-PSAI, median (IQR):	0.07 [0.05;0.09]	0.07 [0.05;0.09]	0.07 [0.05;0.09]	0.962
S-TSAI, median (IQR):	0.43 [0.33;0.53]	0.44 [0.34;0.53]	0.39 [0.32;0.49]	0.115
C-RR, median (IQR):	0.85 [0.77;0.92]	0.84 [0.76;0.92]	0.88 [0.78;0.92]	0.153
C-TAI, median (IQR) (mm):	9.19 [6.78;12.2]	9.15 [6.73;11.7]	9.25 [6.94;12.8]	0.440
C-LSAI, median (IQR):	0.09 [0.06;0.13]	0.08 [0.06;0.12]	0.09 [0.06;0.13]	0.403
C-RSAI, median (IQR):	0.09 [0.06;0.12]	0.09 [0.06;0.12]	0.10 [0.07;0.13]	0.243
C-TSAI, median (IQR):	0.18 [0.13;0.25]	0.18 [0.13;0.24]	0.19 [0.14;0.26]	0.319
A-CSAMU, median (IQR) (mm2):	46.0 [36.6;57.1]	45.3 [34.8;54.9]	46.9 [37.6;57.4]	0.498
MUV, median (IQR) (mm3):	678 [541;839]	669 [523;811]	708 [563;870]	0.266
PV, median (IQR) (ml):	44.2 [36.0;57.8]	44.2 [36.4;57.8]	44.3 [34.5;58.9]	0.833
PSAD, median (IQR) (ng/ml/ml):	0.33 [0.19;0.59]	0.34 [0.19;0.58]	0.32 [0.22;0.59]	0.827
PCI, median (IQR) (mm):	80.9 [76.6;85.4]	81.3 [76.4;85.8]	80.1 [76.6;83.6]	0.188
PV/PCI, median (IQR), median (IQR) (mm2):	0.54 [0.44;0.72]	0.53 [0.44;0.70]	0.56 [0.42;0.73]	0.823
S-BH/AD, median (IQR) (mm):	0.38 [0.21;0.57]	0.37 [0.21;0.56]	0.38 [0.21;0.62]	0.318
BWI, median (IQR):	2.86 [2.55;3.14]	2.86 [2.54;3.14]	2.85 [2.61;3.15]	0.637
SWI, median (IQR) :	2.30 [2.01;2.63]	2.30 [2.02;2.65]	2.31 [2.00;2.60]	0.541
PDI , median (IQR) (mm):	2.77 [2.49;3.08]	2.74 [2.49;3.08]	2.81 [2.48;3.07]	0.878
PDI/PV, median (IQR) (/ml):	0.06 [0.05;0.08]	0.06 [0.05;0.08]	0.06 [0.05;0.08]	0.947
Robot-assisted radical prostatectomy (RARP) , n(%):				
TI-MRI-PB, (IQR) (day):	3.00 [1.00;7.00]	3.00 [1.00;7.00]	3.00 [1.75;5.00]	0.308
TI-PB-S, median (IQR) (day):	14.0 [10.0;20.0]	14.0 [10.0;21.0]	12.5 [8.00;16.0]	0.056
Inpatient ward, n(% ):				0.784
1	138 (49.8%)	95 (49.2%)	43 (51.2%)	
2	113 (40.8%)	81 (42.0%)	32 (38.1%)	
3	26 (9.39%)	17 (8.81%)	9 (10.7%)	
Surgeons:				0.208
1	89 (32.1%)	68 (35.2%)	21 (25.0%)	
2	46 (16.6%)	30 (15.5%)	16 (19.0%)	
3	45 (16.2%)	34 (17.6%)	11 (13.1%)	
4	40 (14.4%)	27 (14.0%)	13 (15.5%)	
Others	57 (20.6%)	34 (17.6%)	23 (27.4%)	
Concomitant surgical procedures :				0.165
No	272 (98.2%)	191 (99.0%)	81 (96.4%)	
Yes	5 (1.81%)	2 (1.04%)	3 (3.57%)	
Number of laparoscopic incisions :				0.835
5	128 (46.2%)	87 (45.1%)	41 (48.8%)	
6	145 (52.3%)	103 (53.4%)	42 (50.0%)	
Others	4 (1.44%)	3 (1.55%)	1 (1.19%)	
Surgical approach:				0.055
Intraperitoneal	201 (72.6%)	133 (68.9%)	68 (81.0%)	
Extraperitoneal	76 (27.4%)	60 (31.1%)	16 (19.0%)	
Lymph node dissection, n(% ):				0.12
No	209 (75.5%)	140 (72.5%)	69 (82.1%)	
Yes	68 (24.5%)	53 (27.5%)	15 (17.9%)	

TURP, Transurethral resection of the prostate; SII, Systemic immune-inflammation index; Neutrophil* Platelet/Lymphocyte; DeRitis ratio=Aspartate aminotransferas/Alanine aminotransferase; eGFR, Estimated glomerular filtration rate; fPSA, Free prostate-specific antigen; tPSA, Total prostate-specific antigen; APTT, Activated partial thromboplastin time; Percentage of PBC, Percentage of positive biopsy cores; PI-RADS v2, Prostate imaging reporting and data system version 2; TI-MRI-PB, The time interval of MRI to prostate biopsy; TI-PB-S, The time interval of prostate biopsy to surgery; MRI measurement abbreviations, names, and definitions were detailed in **Supplementary Table 2**.

### Features selection

**Figure 3:**
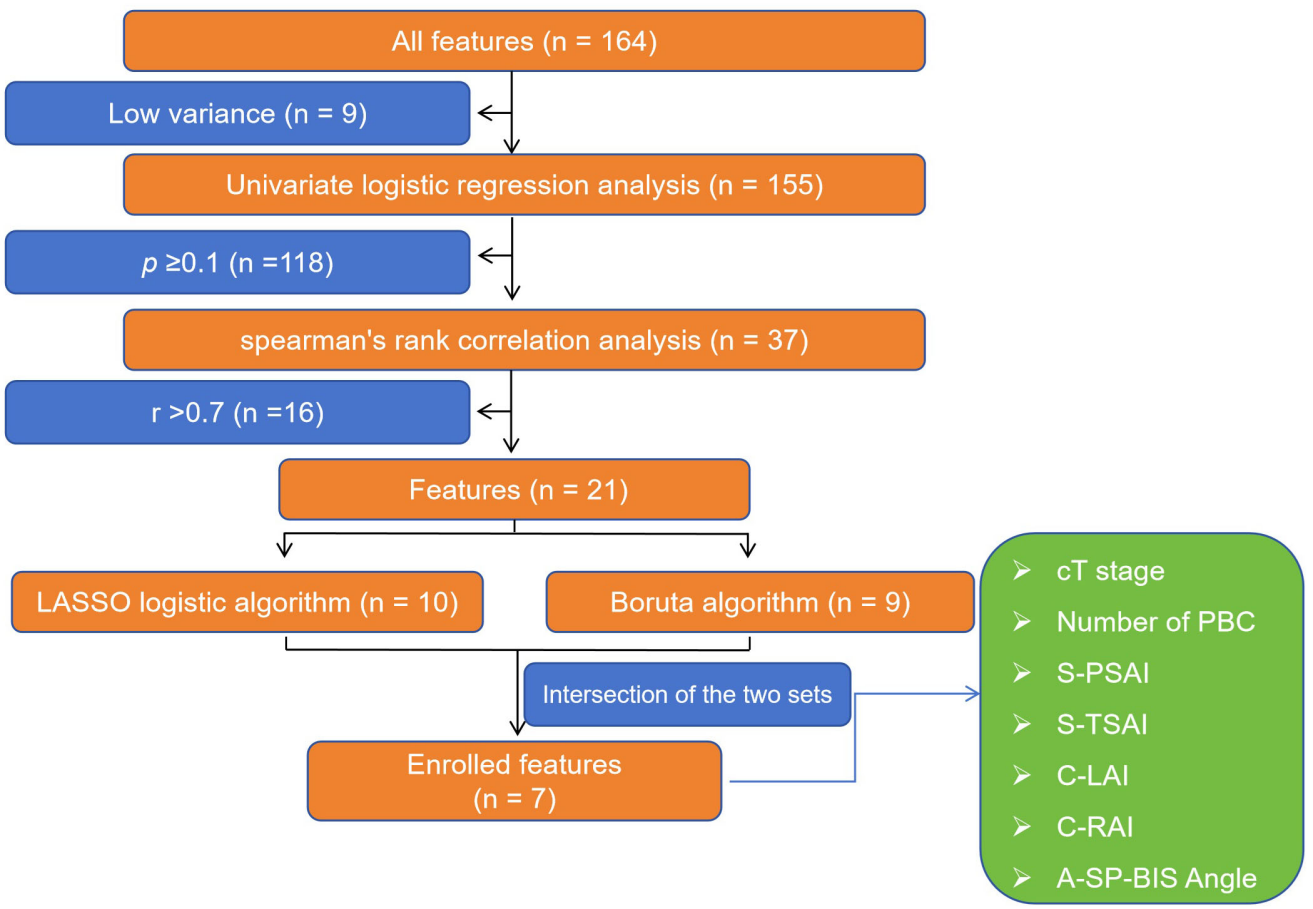
Screening flowchart of key features for model establishment in this study. cT stage, Clinical primary tumor stage. Number of PBC, Number of positive biopsy cores. S-PSAI, Sagittal plane-Posterior spatial anatomical structure index. S-TSAI, Sagittal plane-Total spatial anatomical structure index. C-LAI, Coronal plane-Left anatomical structure interval. C-RAI, Coronal plane-Right anatomical structure interval. A-SP-BIS Angle, Axial plane-Inferior margin of symphysis pubis - bilateral ischial spinous angle. LASSO, the least absolute shrinkage and selection operator.

Based on previous studies (9–15, 21–28) and the authors’ interests. From 164 initial features, 7 key features were retained through four-step screening (**Figure 3**): (1) 8 low-variance features were excluded (**Supplementary Table 3**). (2) 119 features unrelated to PSM were excluded via univariable logistic regression analysis (**Supplementary Table 4**). (3) 16 redundant features (r ≥ 0.7) were removed via Spearman’s rank correlation analysis (**Supplementary Figure 2**). (4) The LASSO logistic algorithm and the Boruta algorithm retained 10 and 9 features (**Supplementary Figure 3**), with final features as their intersection. The 7 features (**Supplementary Figure 3**) included:

▪ Number of positive biopsy cores (Number of PBC).
▪ Clinical tumor stage (cT stage).
▪ Sagittal plane-posterior spatial anatomical structure index (S-PSAI).
▪ Sagittal plane-total spatial anatomical structure index (S-TSAI).
▪ Coronal plane-left anatomical structure interval (C-LAI).
▪ Coronal plane-right anatomical structure interval (C-RAI).
▪ Axial plane-inferior margin of symphysis pubis-bilateral ischial spinous angle (A-SP-BIS Angle).

No significant differences in these 7 features were observed across the training, validation, and test sets (all *P* > 0.05; **Table 2**).

**Table 2:** Key features comparison in the training, validation, and test sets.

Characteristics	[ALL] N=347	[Training set] N=193	[Validation set] N=84	[Test set] N=70	*P*-value
Positive surgical margin (PSM), n(% ) :					0.797
No	238(68.6%)	135 (69.9%)	57 (67.9%)	46(65.7%)	
Yes	109 (31.4%)	58 (30.1%)	27 (32.1%)	24 (34.3%)	
Age, median (IQR) (year):	70.0 [65.0;74.0]	70.0 [65.0;74.0]	68.5 [64.0;73.0]	71.0 [67.0;76.0]	0.088
A-SP-BIS Angle (IQR) (°):	56.6 [53.2;60.2]	56.9 [52.9;60.6]	55.8 [52.8;58.6]	57.0 [54.8;60.7]	0.102
C-RAI, median (IQR) (mm):	4.73 [3.24;6.48]	4.62 [3.06;6.21]	4.64 [3.26;6.84]	5.07 [3.82;6.64]	0.161
C-LAI, median (IQR) (mm):	4.32 [3.09;6.10]	4.22 [2.87;6.10]	4.36 [3.06;6.09]	4.36 [3.43;6.07]	0.488
S-PSAI, median (IQR):	0.07 [0.05;0.09]	0.07 [0.05;0.09]	0.07 [0.05;0.09]	0.07 [0.05;0.09]	0.907
S-TSAI, median (IQR):	0.43 [0.33;0.54]	0.44 [0.34;0.53]	0.39 [0.32;0.49]	0.48 [0.35;0.57]	0.109
cT stage, n(%):					0.694
1	36 (10.4%)	19 (9.84%)	8 (9.52%)	9 (12.9%)	
2	216 (62.2%)	118 (61.1%)	50 (59.5%)	48 (68.6%)	
3	79 (22.8%)	47 (24.4%)	21 (25.0%)	11 (15.7%)	
4	16 (4.61%)	9 (4.66%)	5 (5.95%)	2 (2.86%)	
Number of PBC, median (IQR):	5.00 [3.00;7.00]	5.00 [3.00;7.00]	5.00 [2.75;7.00]	5.00 [3.00;7.00]	0.320

cT stage, Clinical primary tumor Stage; Number of PBC, Number of positive biopsy cores; MRI measurement abbreviations, names, and definitions were detailed in **Supplementary Table 2**.

### ML model establishment

**Figure 4:**
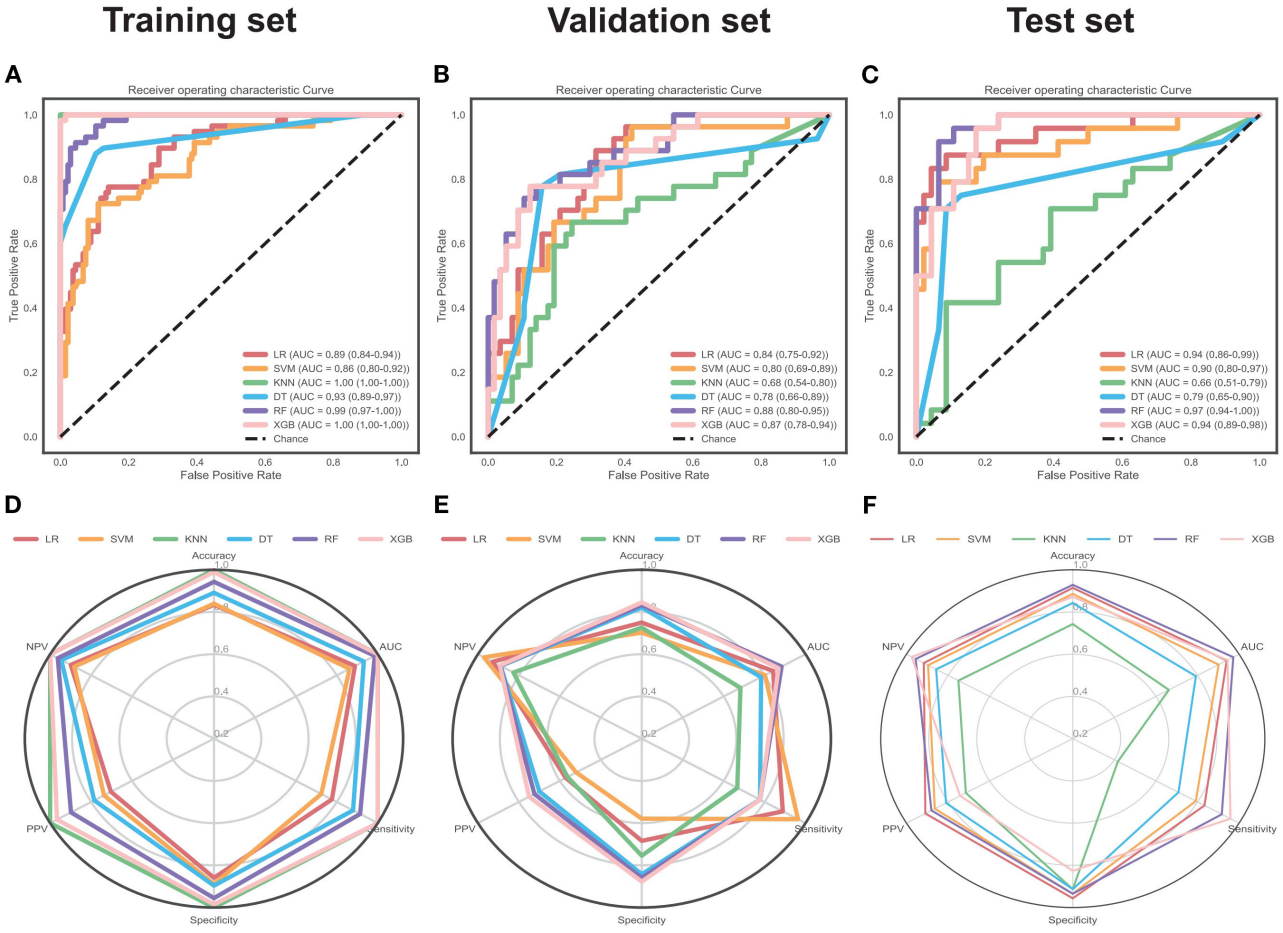
Predictive performance of six ML models. This figure presents the Receiver Operating Characteristic (ROC) curve analysis of the established models in the training **(A)**, validation **(B)**, and test **(C)** sets, as well as the radar plots of the models’ prediction metrics in the training **(D)**, validation **(E)**, and test **(F)** sets. LR, Logistic Regression; SVM, Support Vector Machine; KNN, K-Nearest Neighbors; DT, Decision Tree; RF, Random Forest; XGB, Extreme Gradient Boosting; AUC, Area Under the ROC Curve; PPV, Positive Predictive Value; NPV, Negative Predictive Value.

**Table 3** summarizes model parameters, adjustment ranges, and optimal values via Bayesian optimization. Among the six models, KNN and XGB showed high training-set AUCs of 1.00 (95% CI: 1.00-1.00), suggesting overfitting. The RF model achieved optimal balanced performance: (1) Training set: AUC of 0.99 (95% CI: 0.97–1.00), accuracy of 0.94. (2) Validation set: AUC of 0.88 (95% CI: 0.80–0.95), accuracy of 0.83. (3) Test set: AUC of 0.97 (95% CI: 0.94–1.00), accuracy of 0.93. The DT, SVM, and LR models ranked as the second, third, and fourth predicting models in the training set. The DT, LR, and SVM models ranked as the second, third, and fourth predicting models in the validation set. The LR, SVM, and DT models ranked as the second, third, and fourth predicting models in the test set. Model performance metrics are detailed in **Table 4** and **Figures 4A–F**.

**Table 3:** Model parameters screening via Bayesian analysis.

Models	Parameter and adjustment range	Optimal parameters
LR	C': (0.01, 5)	4.664
	penalty': ['l1', 'l2']	l1'
SVM	C': (0.01, 5),	4.397
	kernel': ['linear', 'rbf'],	'linear'
	gamma': (0.001, 1.0, 'log-uniform')	0.013
KNN	n_neighbors': (3, 20)	9
	weights': ['uniform', 'distance']	distance'
	p': (1, 2)	2
DT	max_depth': (2, 8),	4
	min_samples_split': (2, 10),	10
	min_samples_leaf': (1, 5)	1
RF	n_estimators': (10, 100),	76
	max_depth': (2, 8),	8
	min_samples_split': (2, 10),	10
	min_samples_leaf': (1, 5),	4
	bootstrap': [True, False],	TRUE
XGB	n_estimators': (10, 100),	88
	max_depth': (2, 8),	8
	learning_rate': (0.01, 0.5, 'log-uniform'),	0.047
	subsample': (0.7, 1.0),	0.828
	colsample_bytree': (0.7, 1.0)	0.873

LR, Logistic Regression; SVM, Support Vector Machine; KNN, K-Nearest Neighbors; DT, Decision Tree; RF, Random Forest; XGB, Extreme Gradient Boosting.

**Table 4:** Predictive performance of six ML models.

Data set	Models	Accuracy	AUC (95% CI)	Sensitivity	Specificity	PPV	NPV	F1 score
Training set	LR	0.83	0.89 (0.84-0.93)	0.78	0.86	0.7	0.9	0.74
	SVM	0.84	0.86 (0.80-0.91)	0.72	0.89	0.74	0.88	0.73
	KNN	1.00	1.00 (1.00-1.00)	1.00	1.00	1.00	1.00	1.00
	DT	0.89	0.93 (0.89-0.97)	0.88	0.90	0.78	0.95	0.83
	RF	0.94	0.99 (0.97-1.00)	0.91	0.96	0.9	0.96	0.91
	XGB	0.99	1.00 (1.00-1.00)	1.00	0.99	0.97	1.00	0.98
Validation set	LR	0.75	0.84 (0.75-0.92)	0.89	0.68	0.57	0.93	0.70
	SVM	0.70	0.80 (0.70-0.89)	0.96	0.58	0.52	0.97	0.68
	KNN	0.73	0.68 (0.56-0.81)	0.67	0.75	0.56	0.83	0.61
	DT	0.82	0.78 (0.66-0.89)	0.78	0.84	0.70	0.89	0.74
	RF	0.83	0.88 (0.80-0.95)	0.78	0.86	0.72	0.89	0.75
	XGB	0.85	0.87 (0.77-0.94)	0.78	0.88	0.75	0.89	0.76
Test set	LR	0.91	0.94 (0.86-0.99)	0.83	0.96	0.91	0.92	0.87
	SVM	0.89	0.90 (0.80-0.97)	0.79	0.93	0.86	0.90	0.83
	KNN	0.74	0.66 (0.51-0.79)	0.42	0.91	0.71	0.75	0.53
	DT	0.84	0.79 (0.65-0.90)	0.71	0.91	0.81	0.86	0.76
	RF	0.93	0.97 (0.94-1.00)	0.92	0.93	0.88	0.96	0.90
	XGB	0.87	0.94 (0.89-0.98)	0.96	0.83	0.74	0.97	0.84

PPV, Positive Predictive Value; NPV, Negative Predictive Value; LR, Logistic Regression; SVM, Support Vector Machine; KNN, K-Nearest Neighbors; DT, Decision Tree; RF, Random Forest; XGB, Extreme Gradient Boosting.

### Comparison of ML models

DeLong tests confirmed the RF model outperformed LR, SVM, and DT in the training set (all *P* < 0.05) but not KNN/XGB (overfitting models). In validation and test sets, RF outperformed all five other models (positive Z-scores). In the validation set, RF showed non-significant differences vs. LR/XGB (*P* > 0.05) but superiority vs. SVM/KNN/DT (*P* < 0.05). In the test set, RF showed non-significant differences vs. LR/SVM/XGB (*P* > 0.05) but superiority vs. KNN/DT (*P* < 0.05) (**Table 5**).

**Table 5:** Results of DeLong’s test analysis comparing AUCs of the RF model with those of other ML models.

Data set	Models	Z-score	*P*-value
Training set	RF vs. LR	4.6157	0.0000
	RF vs. SVM	4.6805	0.0000
	RF vs. KNN	-2.4243	0.0153
	RF vs. DT	3.0600	0.0022
	RF vs. XGB	-2.4662	0.0137
Validation set	RF vs. LR	1.3588	0.1742
	RF vs. SVM	2.1151	0.0344
	RF vs. KNN	3.7698	0.0002
	RF vs. DT	2.7313	0.0063
	RF vs. XGB	0.9903	0.3220
Test set	RF vs. LR	1.1390	0.2547
	RF vs. SVM	1.9091	0.0562
	RF vs. KNN	4.6554	0.0000
	RF vs. DT	3.1687	0.0015
	RF vs. XGB	1.8528	0.0639

LR, Logistic Regression; SVM, Support Vector Machine; KNN, K-Nearest Neighbors; DT, Decision Tree; RF, Random Forest; XGB, Extreme Gradient Boosting.

**Figure 5:**
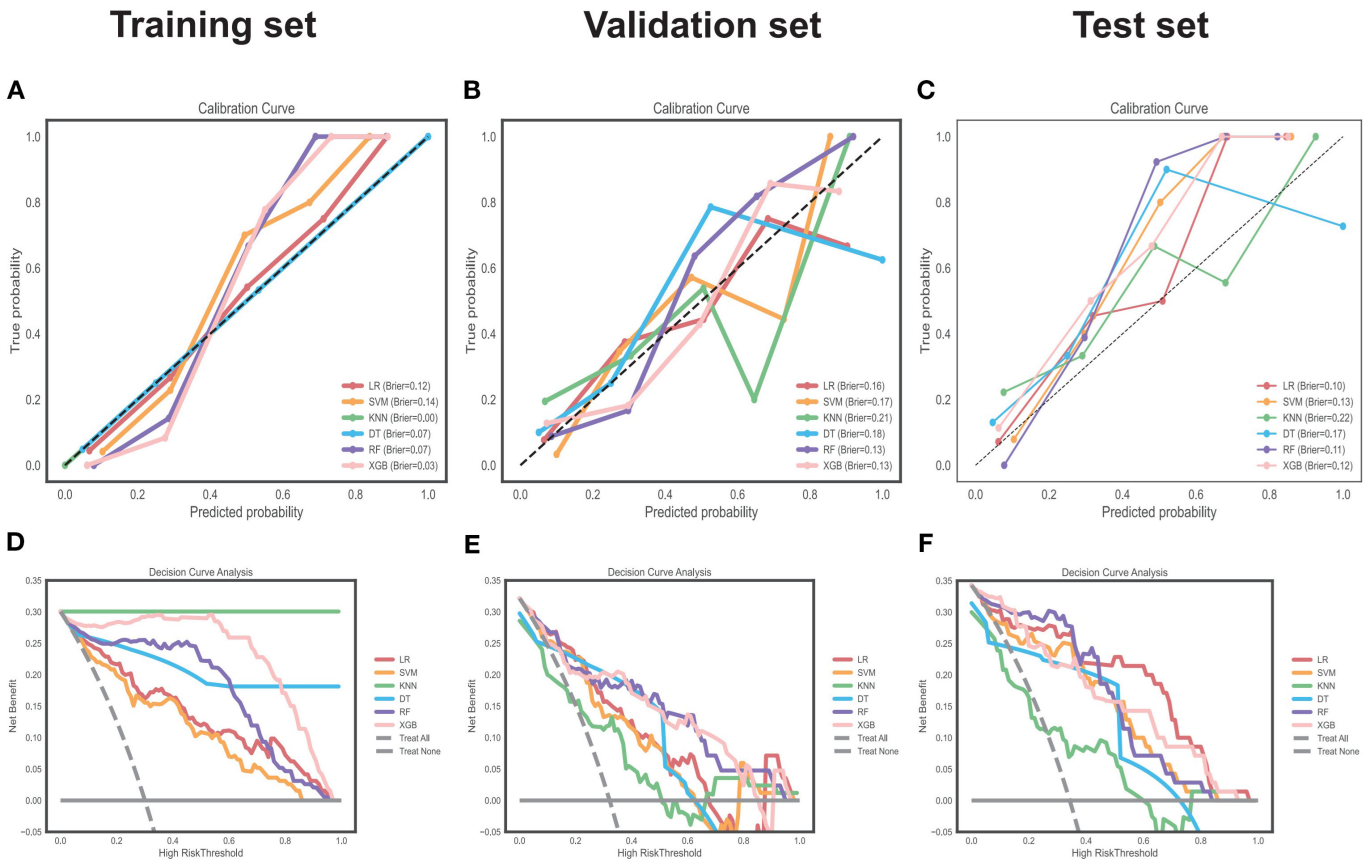
Clinical utility evaluation via calibration and decision curves. Calibration curve analysis of the six models in the training **(A)**, validation **(B)** and test **(C)** sets. Decision curve analysis of the six models in the training **(D)**, validation **(E)** and test **(F)** sets. "Treat All" and "Treat None" are baselines. Different colors represent models. Brier score, measure of calibration (lower = better).

Also, RF had the lowest Brier scores (except overfitted KNN/XGB models) and well-matched calibration curves (**Figures 5A–C**), with the highest net benefit in most threshold probabilities at decision curve analysis (**Figures 5D–F**).

### Robustness checks

**Figure 6:**
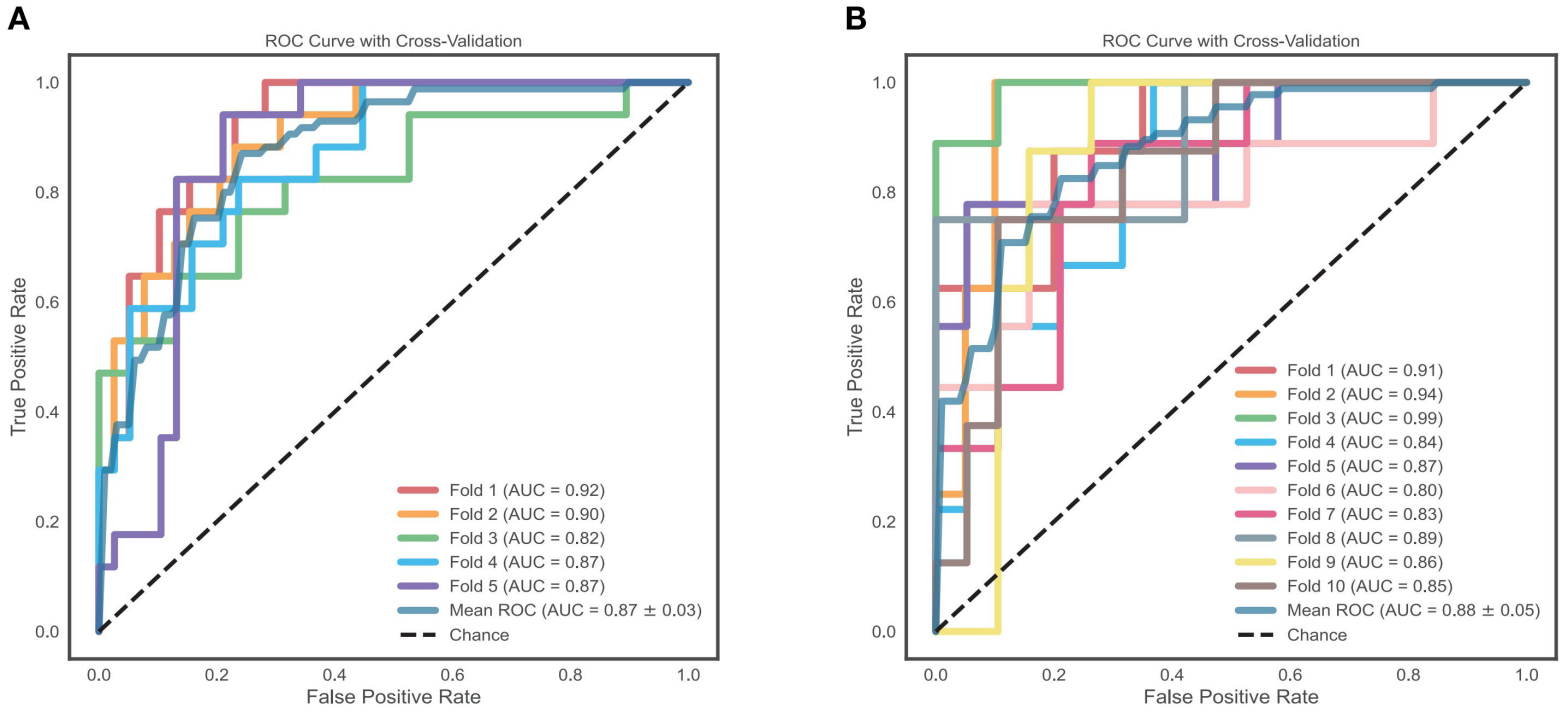
Five-fold and ten-fold cross-validation of the RF model. **(A)** Five-fold cross-validation ROC curves of the RF model (AUC values range from 0.82 to 0.92, mean AUC = 0.87, 95% CI: 0.84–0.90); **(B)** Ten-fold cross-validation ROC curves of the RF model (AUC values range from 0.80 to 0.99, mean AUC = 0.88, 95% CI: 0.83–0.93). The dashed line represents chance (AUC = 0.5). RF, Random Forest; AUC, Area Under the ROC Curve; CI, Confidence Interval.

Five-fold cross-validation for RF showed fold-specific AUCs of 0.82–0.92, with a mean AUC of 0.87 (95% CI: 0.84–0.90). Ten-fold cross-validation showed fold-specific AUCs of 0.80–0.99, with a mean AUC of 0.88 (95% CI: 0.83–0.93), indicating stable performance (**Figure 6**).

### SHAP interpretation of the RF model

**Figure 7:**
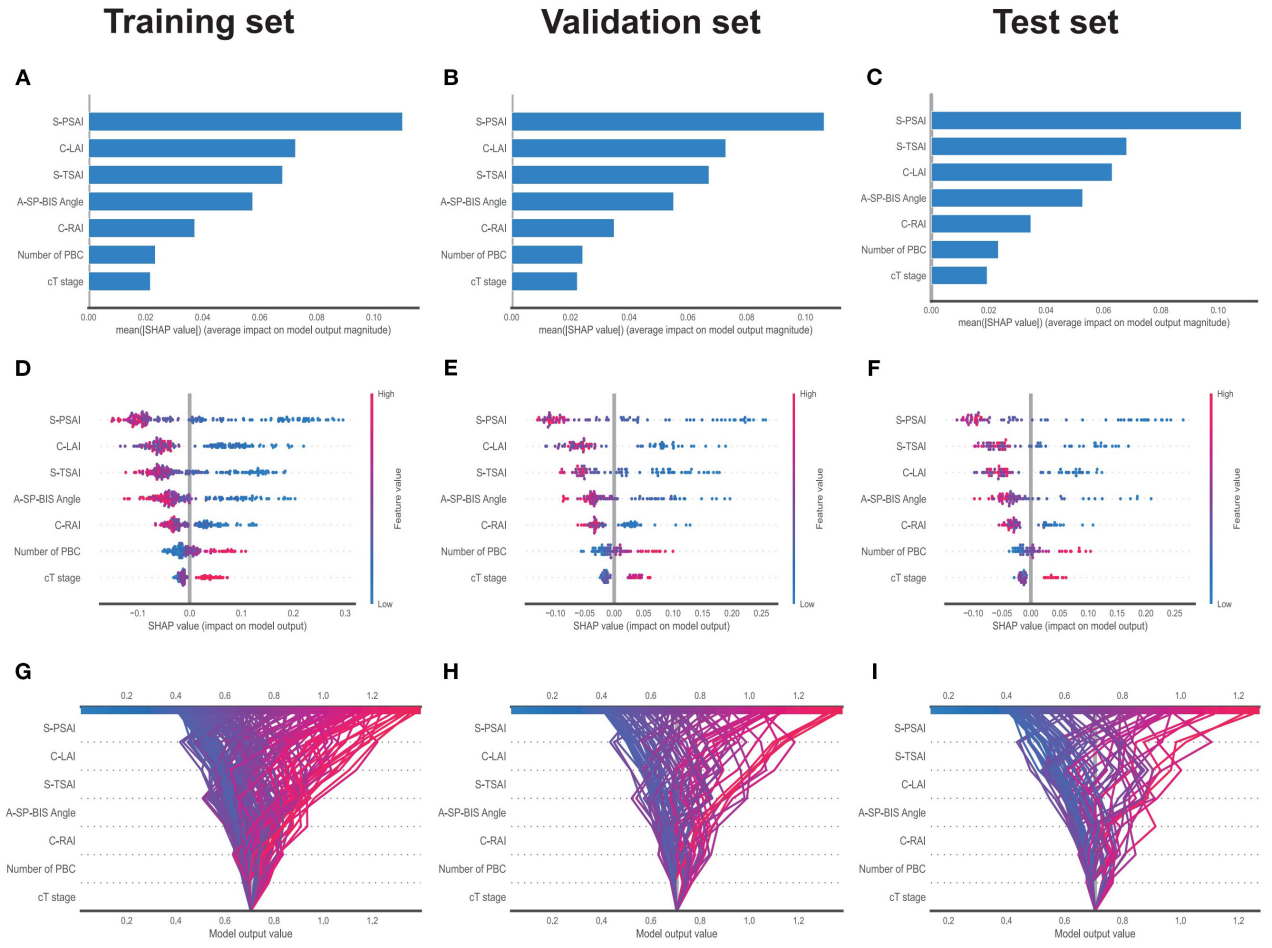
The SHAP analysis of the RF model. Bar plot of mean SHAP values (feature importance) in the training, validation and test sets **(A–C)**; Bee-swarm plot of SHAP values (color gradients represent feature value by impact) in the training, validation and test sets **(D–F)**; Parallel plot of model output values and feature impact in the training, validation and test sets **(G–I)**; SHAP, Shapley Additive exPlanations; RF, Random Forest; S-PSAI, Sagittal plane-posterior spatial anatomical structure index; C-LAI, Coronal plane-left anatomical structure interval; S-TSAI, Sagittal plane-total spatial anatomical structure index; A-SP-BIS Angle, Axial plane-inferior margin of symphysis pubis-bilateral ischial spinous angle; C-RAI, Coronal plane-right anatomical structure interval; Number of PBC, Number of positive biopsy cores; cT stage, Clinical primary tumor Stage.

**Figure 8:**
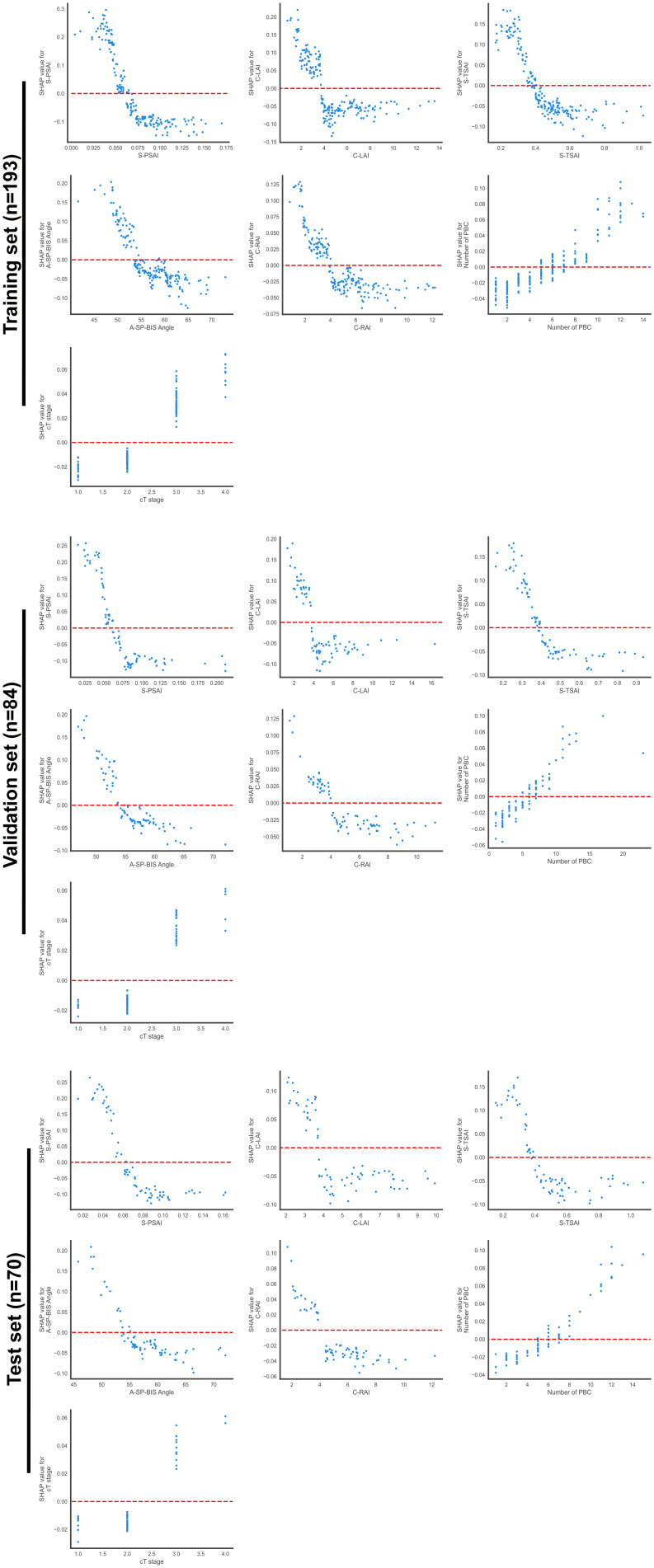
SHAP dependence plots for seven features. SHAP dependence plot is a visualization tool in SHAP tools used to analyze the influence of a single feature on model prediction and its interaction with other features, which reveals the direct impact (positive or negative) and nonlinear relationship of features on the prediction results by presenting the relationship between feature values and the corresponding SHAP values.

**Figure 9:**
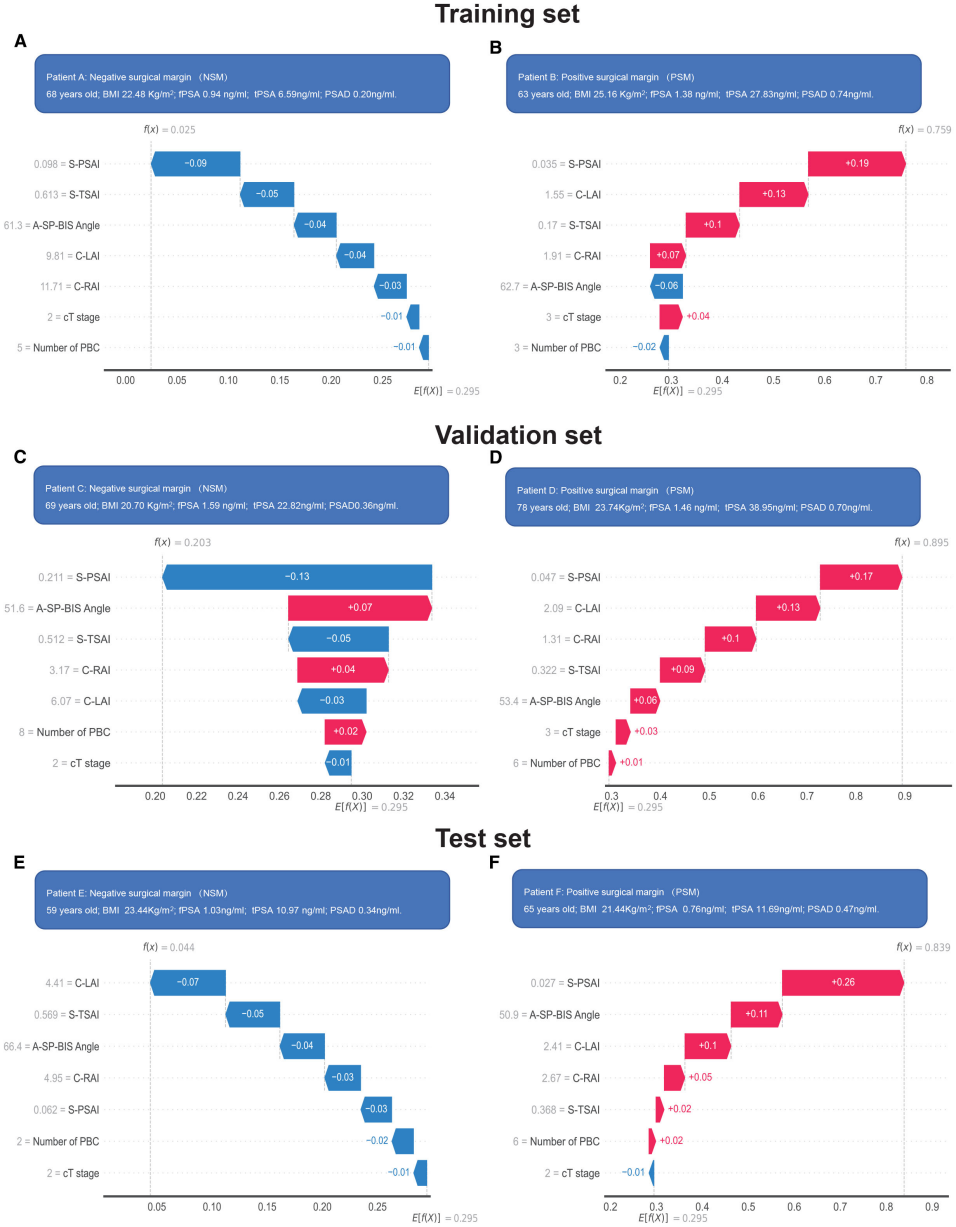
Representative NSM **(A, C, E)** and PSM **(B, D, F)** cases predicted by the RF model. Representative NSM (negative surgical margin) case in the training **(A)**, validation **(C)** and test **(E)** sets; Representative PSM (positive surgical margin) case in the training **(B)**, validation **(D)** and test **(F)** sets. The distinct contributions of each feature within the RF model for individual predictions are illustrated using the SHAP waterfall plot. RF, random forest. SHAP, SHapley Additive exPlanations.

Feature importance rankings were consistent across the training and validation datasets: S-PSAI > C-LAI > S-TSAI > A-SP-BIS Angle > C-RAI > Number of PBC > cT stage (**Figures 7A, B**). In the test set, the ranking in the test set was: S-SPAI > S-TSAI > C-LAI > A-SP-BIS Angle > C-RAI > Number of PBC > cT stage (**Figures 7A-C**). Five spatial features (S-PSAI, C-LAI, S-TSAI, A-SP-BIS Angle, C-RAI) were negatively associated with PSM risk, while Number of PBC and cT stage were positively associated (**Figures 7D–F**). The SHAP decision plot illustrates the influences of all contributing features on the final predicted probability (**Figures 7G-I**). SHAP dependence plots further clarified feature relationships (**Figure 8**). Representative cases (NSM vs. PSM) illustrated feature contributions of each of the 7 key features within the RF model (**Figure 9**).

## Discussion

To our knowledge, this is the first study to investigate ML models that integrate clinical, mpMRI, and biopsy pathology data for predicting PSM before RARP. The RF model exhibited excellent performance across the training, validation, and test sets, with its robustness validated via cross-validation. SHAP analysis identified the feature importance rankings, thereby improving model transparency. This innovative approach will improve preoperative surgical risk stratification, optimize clinical decision-making processes, and establish a framework for automated robotic surgery case screening, ultimately advancing the precision and individualization of RARP therapeutic strategies.

Previous studies have identified predictors of PSM, including surgical experience, body mass index (BMI), tPSA, extracapsular extension (ECE), neurovascular bundle (NVB) invasion, cT stage, percentage of positive biopsy cores, number of positive biopsy cores, Gleason score (GS), pathological stage (pT stage), time interval between prostate biopsy and surgery, among others (9–14), but these studies focused on single feature types. Multiparametric MRI (mpMRI), a standard PCa imaging tool (38–41), provides critical anatomical insights, with prior studies linking prostate and pelvic dimensions (e.g., prostate volume (PV), pelvic dimension index (PDI)/PV ratio, prostate-muscle index (PMI), apical depth (AD), symphysis angle (SA), transverse diameter of the pelvic entrance and intertuberous distance (ITD), among others) to PSM risk (21–24, 26, 28, 42, 43). However, focusing exclusively on a single category of features while neglecting their holistic nature when evaluating PSM in RARP offers a limited perspective. This study addressed this limitation by integrating 164 features across multiple domains and screening 7 key predictors through a rigorous multi-step selection process, thereby ensuring the scientific validity and rigor of the selected features.

Optimal hyperparameter tuning is critical for ML performance (44). Data-efficient optimization algorithms, such as Bayesian optimization (44), were employed to automate this process, screening both the parameter adjustment range and optimal parameters. Based on evaluation metrics, KNN and XGB approached or reached a value of 1 for accuracy, AUC, and other metrics, indicating overfitting. The KNN and XGB regression methods were susceptible to overfitting and fit discontinuity, which remain significant challenges in the field (45). In contrast, the RF model obtained suitable AUCs of 0.99 (95% CI: 0.97-1.00), 0.88 (95% CI: 0.80-0.95), and 0.97 (95% CI: 0.94-1.00) in the training, validation, and test sets. The RF outperformed LR, SVM, and DT, indicating superior generalization. Its high accuracy (0.94, 0.83, 0.93), specificity (0.96, 0.86, 0.93), and sensitivity (0.91, 0.78, 0.92) across datasets confirm its predictive reliability. Overall, these results confirm that the RF model is the optimal classifier, consistent with previous studies (46, 47).

Notably, the DeLong test confirmed that in the training set, the AUCs of the RF model were superior to those of LR, SVM, and DT, but lower than those of KNN and XGB. Given that KNN and XGB are overfitting models, these comparisons lack practical significance. The AUCs of the RF model were comparable to those of LR and XGB in the validation set, but superior to those of SVM, KNN, and DT. The AUCs of the RF model were comparable to those of LR, SVM, and XGB in the test set, but superior to those of KNN and DT. The DeLong test confirmed that the overall advantage of the RF model holds, but this advantage has practical value for high-variance models (KNN/DT/SVM models). For LR and XGB models in the validation and test sets, the RF model does not exhibit a significant advantage, which may be attributed to the insufficient sample size of the current validation and test sets. In addition, the RF model exhibited the optimal calibration (lower Brier score, well-aligned calibration curves) and the highest net benefit across most threshold probabilities (decision curve analysis). In conclusion, the RF model demonstrated excellent performance in terms of sensitivity, specificity, accuracy, ROC, and F1 score across the three sets, affirming its predictive reliability and clinical decision-support value. Five-fold and ten-fold cross-validation confirmed the stable performance of the RF model. These results suggest that the RF model could facilitate the identification of surgical difficulty, guide personalized surgical planning, and optimize resource allocation (such as assigning experienced surgeons), thereby reducing the risk of biochemical recurrence after surgery.

ML models are often criticized as “black boxes” (48, 49), which limits their clinical acceptance, particularly in critical applications such as healthcare, where transparency and reliability in clinical decision-making tools are crucial (50, 51). To address this challenge, researchers have focused on developing methods to improve the interpretability of these models, such as SHapley Additive exPlanations (SHAP) analysis, which assigns contribution values to individual features in the dataset to indicate the extent of each feature’s influence on predicted outcomes. This holistic approach enables researchers to identify which features most significantly impact outcomes and whether their influence is positive or negative, thereby promoting the acceptance of ML-based diagnostic or predictive tools in clinical settings (47, 52–56). To our knowledge, this is the first study to investigate ML models based on multi-dimensional fusion data that use SHAP methods for PSM prediction. The contribution relationships of the 7 selected features were successfully visualized using SHAP bar plots, bee-swarm plots, and decision plots. As a result, our study identified that 5 newly discovered spatial features were negatively associated with PSM, with S-PSAI being the most influential. In preoperative PSM prediction, the RF model assigns the highest importance to this feature. Specifically, lower values of S-PSAI, S-TSAI, C-LAI, and C-RAI indicate limited surgical space, which increases surgical difficulty and the risk of tumor residue, prompting surgeons to adjust dissection techniques (such as expanding the resection range or performing more meticulous operations) or assign experienced surgeons. A narrow A-SP-BIS Angle could inform surgical planning (such as adjusting port placement to improve access). However, the number of PBC and cT stage are positively correlated with PSM, consistent with previous studies (11, 12, 15). Surgeons can use feature contributions to prioritize intraoperative vigilance in high-risk regions. SHAP visualizations enhance transparency, thereby improving trust in model-derived decisions and patient understanding and compliance.

This study has several key strengths: (1) Robust data quality: Strict inclusion and exclusion criteria were applied, and enrolled cases underwent rigorous screening; (2) Comprehensive feature integration: Integration of radiomics, prostate and pelvic measurements, clinical, and biopsy pathological features, comprehensively covering factors influencing PSM; (3) Scientific rigor in feature screening: A four-step screening process (low-variance elimination, univariate regression, Spearman correlation-based redundancy removal, and intersection of LASSO and Boruta algorithms) was applied to 164 initial features; (4) Model diversity and validation: Six ML algorithms were used to develop models, and stable predictive models were identified through comparison, with robustness confirmed via sensitivity analysis and N-fold (5-fold and 10-fold) cross-validation; (5) Model interpretability: The SHAP method was employed to clarify the model’s decision-making process, providing valuable insights into its predictive mechanism.

Several limitations of this study should be acknowledged. (1) Retrospective bias: The retrospective design introduced inevitable selection bias, highlighting the need for prospective studies with predefined criteria. (2) Selection bias: The high exclusion rate (primarily due to missing data) may introduce selection bias, as patients with complete data may differ from those excluded. (3) Limited generalizability: Although the dataset is larger than those in previous studies, the single-center Chinese cohort limits global applicability, necessitating external and multi-ethnic validation. (4) Automation limitations: Automatic mpMRI feature recognition is lacking and will be addressed in future work. (5) Restricted surgical scope: Patients undergoing laparoscopic RP were excluded because the surgical assistant’s experience level may impact outcomes, and thus the generalizability of the RF model to RP patients requires further investigation. (6) Data limitations: Genomic data (e.g., PTEN deletion) were not included; future iterations will integrate genomic data and long-term functional outcomes to provide a more comprehensive risk assessment.

In conclusion, ML models based on multi-dimensional fusion data improve PSM prediction in RARP. The RF model, with robust performance and SHAP-based interpretability, enhances preoperative risk stratification, optimizes decision-making, and supports personalized treatment, thereby improving patient treatment compliance and potentially enhancing patient outcomes. Prospective and external validation are required prior to clinical implementation.

## Data Availability

The raw data supporting the conclusions of this article will be made available by the authors, without undue reservation.

## Supplementary material

Supplementary Figure 1 Definitions and images of parameters in MRI measurements. Note: MRI measurement abbreviations, names, and definitions were detailed in **Supplementary Table 2**.

Supplementary Figure 2 Feature elimination via Spearman’s rank correlation analysis. Note: A-RSAI, Axial plane-Right spatial anatomical structure index. A-RAI, Axial plane-Right anatomical structure interval. A-SP-BIS Angle, Axial plane-Inferior margin of symphysis pubis - bilateral ischial spinous angle. A-ASP, Axial plane-The angle of the symphysis pubis. C-TSAI, Coronal plane-Total spatial anatomical structure index. C-RSAI, Coronal plane-Right spatial anatomical structure index. C-LSAI, Coronal plane-Left spatial anatomical structure index. C-TAI, Coronal plane-Total anatomical structure interval. C-RAI, Coronal plane-Right anatomical structure interval. C-LAI, Coronal plane-Left anatomical structure interval. S-TSAI, Sagittal plane-Total spatial anatomical structure index. S-PSAI, Sagittal plane-Posterior spatial anatomical structure index. S-ASAI, Sagittal plane-Anterior spatial anatomical structure index. S-TAI, Sagittal plane-Total anatomical structure interval. S-PAI, Sagittal plane-Posterior anatomical structure interval. S-AAI, Sagittal plane-Anterior anatomical structure interval. S-SP-PA-CA Angle, Sagittal plane-Symphysis pubis-prostate apical-coccyx apical angle. S-SP-PA-S5 Angle, Sagittal plane-Symphysis pubis-prostate apical-S5 angle. S-SP-PA-S1 Angle, Sagittal plane-Symphysis pubis-prostate apical-S1 angle. S-MTSP-IMSPA Angle, Sagittal plane-Angle between the medial tangent of the symphysis pubis, and the line connecting the inferior margin of the symphysis pubis and the prostate apical. S-LASP-API Angle, Sagittal plane-Angle between the long axis diameter of the symphysis pubis and anteroposterior diameter of the pelvic outlet angle. PSAD, Total prostate-specific antigen density. AFSI, Anterior fibromuscular stroma invasion. UI, Urethral invasion. A-CCL-PZ, Axial plane-Cumulative contact length-prostate peripheral zone. A-LLD, Axial plane-Largest lesion diameter. cT stage, Clinical primary tumor stage. GGG, Gleason grade group. GS, Gleason score. PGG, Primary Gleason grade. tPSA, Total prostate-specific antigen. fPSA, Free prostate-specific antigen. ALT, Alanine aminotransferase. AST, Aspartate aminotransferas. LND, Lymph node dissection.

Supplementary Figure 3 Feature screening via LASSO and Boruta algorithms. Note: LND, Lymph node dissection. A-ASP, Axial plane-The angle of the symphysis pubis. A-RAI, Axial plane-Right anatomical structure interval. PGG, Primary Gleason grade. S-LASP-API Angle, Sagittal plane-Angle between the long axis diameter of the symphysis pubis and anteroposterior diameter of the pelvic outlet angle. fPSA, Free prostate-specific antigen. AFSI, Anterior fibromuscular stroma invasion. S-SP-PA-S5 Angle, Sagittal plane-Symphysis pubis-prostate apical-S5 angle. AST, Aspartate aminotransferas. S-MTSP-IMSPA Angle, Sagittal plane-Angle between the medial tangent of the symphysis pubis, and the line connecting the inferior margin of the symphysis pubis and the prostate apical. UI, Urethral invasion. cT stage, Clinical primary tumor stage. PSAD, Total prostate-specific antigen density. Number of PBC, Number of positive biopsy cores. A-LLD, Axial plane-Largest lesion diameter. C-RAI, Coronal plane-Right anatomical structure interval. C-LAI, Coronal plane-Left anatomical structure interval. S-TSAI, Sagittal plane-Total spatial anatomical structure index. A-SP-BIS Angle, Axial plane-Inferior margin of symphysis pubis - bilateral ischial spinous angle. S-PSAI, Sagittal plane-Posterior spatial anatomical structure index. LASSO, the least absolute shrinkage and selection operator.

Supplementary Figure 4 Definitions and images of five key features in MRI measurements. Note: **(A)** Axial plane of MRI on T2 showing the maximum area of the prostate. A-SP-BIS-Angle was defined as the angle between the lines connecting the inferior margin of the symphysis pubis to the medial aspects of the bilateral ischial spinous processes. **(B)** Sagittal plane of MRI on T2 showing the maximum area of the prostate. Prostate maximum anteroposterior diameter(PAD) was defined as The maximum anteroposterior diameter at the largest area of the prostate(bc). Prostate maximum craniocaudal diameter(PCD) was defined as The maximum craniocaudal diameter at the largest area of the prostate(ef). Anterior anatomical structure interval diameter(S-AAI) was defined as the horizontal distance between the posterior margin of the symphysis pubis and the anterior margin of the prostate, which was measured at the level of the maximum anteroposterior diameter with the largest area of the prostate (ab). Posterior anatomical structure interval diameter(S-PAI) was defined as the horizontal distance between the posterior margin of the prostate and the anterior margin of the rectum, which was measured at the level of the maximum anteroposterior diameter with the largest area of the prostate (cd). Posterior spatial anatomical structure index(S-PSAI), Calculated based on the formula: (S-RAI) / (S-PAD); Total spatial anatomical structure index(S-TSAI), Calculated based on the formula: ('S-AAI'+'S-PAI') / (S-PAD). (C) Coronal plane of MRI on T2 showing the maximum area of the prostate. Prostate transverse diameter(C-PTD) was defined as the maximum transverse diameter at the largest area of the prostate(bc). Prostate craniocaudal diameter(C-PCD) was defined as the maximum craniocaudal diameter at the largest area of the prostate and the inner edge of the left obturator internus muscle(ef). Left anatomical structure interval(C-LAI) was defined as the horizontal distance between the left edge at the maximum transverse diameter of the prostate and the inner edge of the left obturator internus muscle(cd). Right anatomical structure interval(C-RAI) was defined as the horizontal distance between the horizontal distance between the right edge at the maximum transverse diameter of the prostate and the inner edge of the right obturator internus muscle(ab). P, prostate. SP, symphysis pubis. OIM, obturator internus muscle. Fe, femoral bone. R, rectum. Bl, bladder.
