# Supplementary figures and images for: Interpretable machine learning models based on multi-dimensional fusion data for predicting positive surgical margins in robot-assisted radical prostatectomy: a retrospective study
Source: Front Oncol. 2025 Oct 3;15:1661695. doi: 10.3389/fonc.2025.1661695 (PMC12531042; doi:10.3389/fonc.2025.1661695)

Axial plane

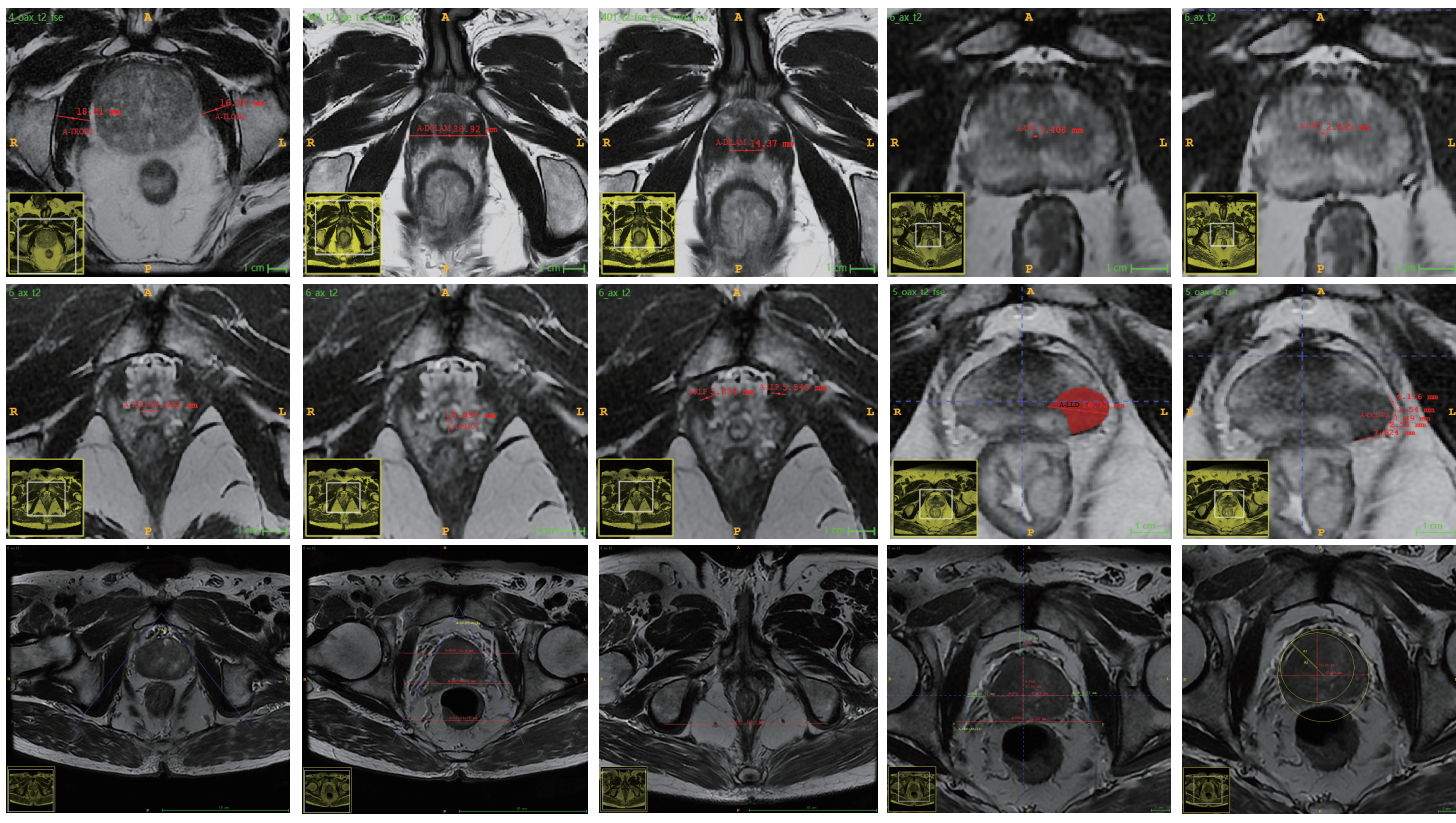

Sagittal plane

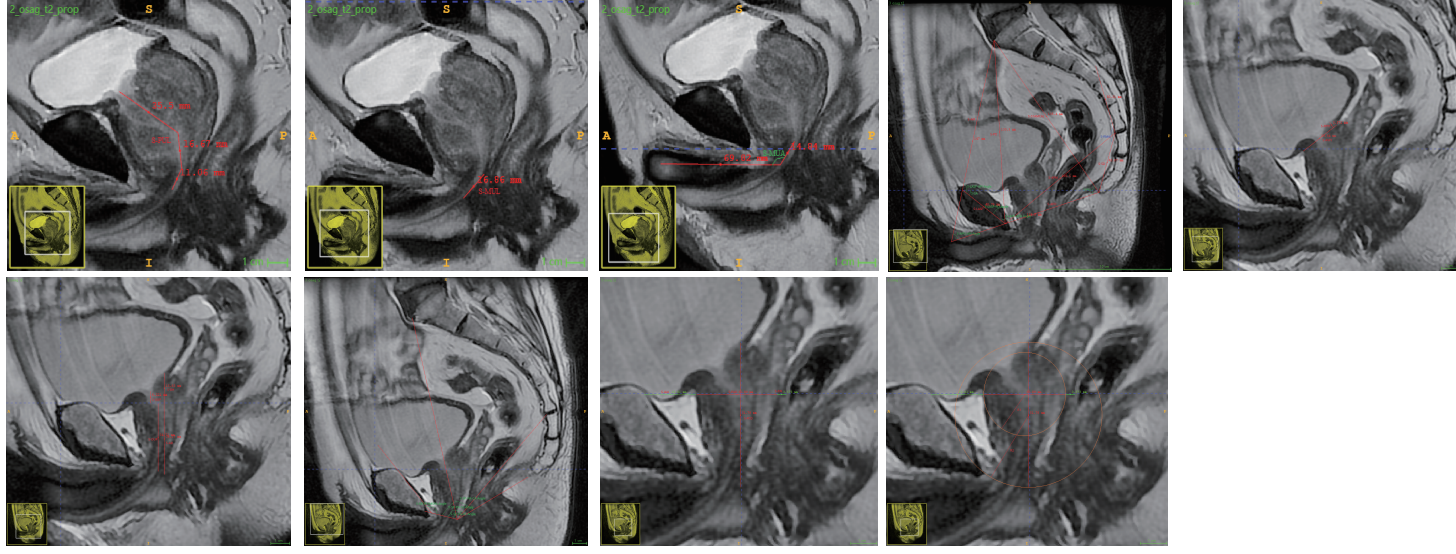

Coronal plane

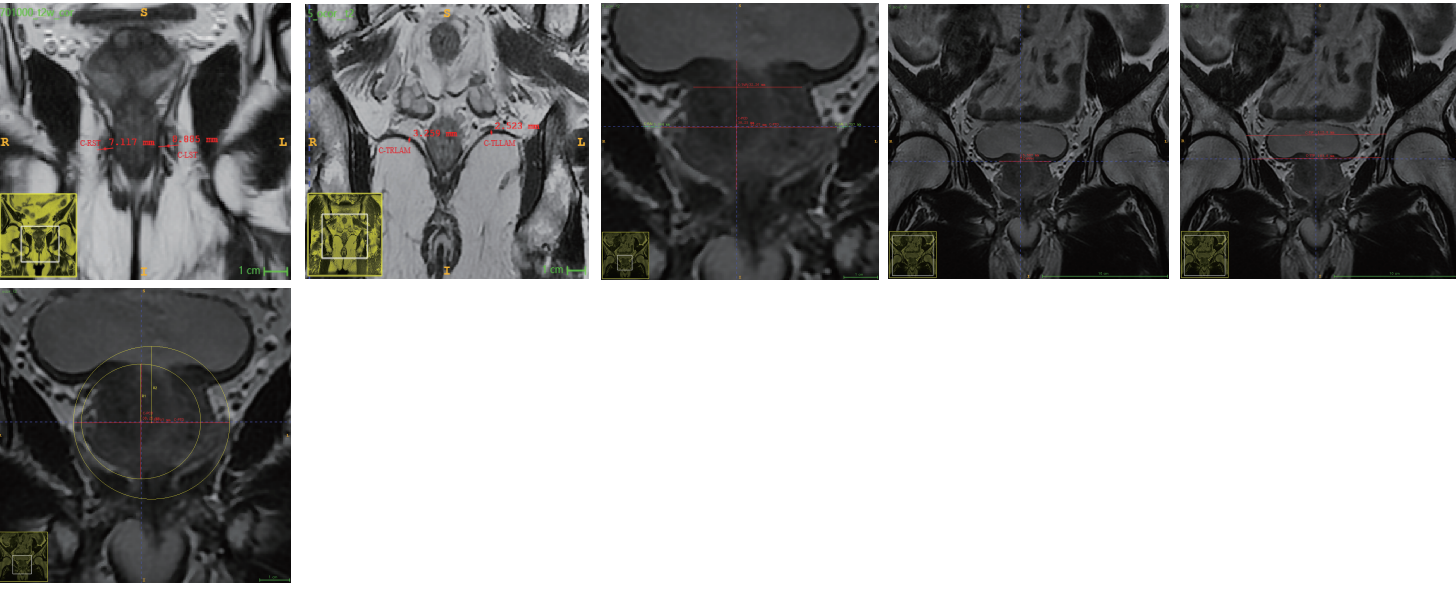

Supplement: Supplementary Figure 1 — Definitions and images of parameters in MRI measurements. Note: MRI measurement abbreviations, names, and definitions were detailed in Supplementary Table 2. [file Image1.pdf]

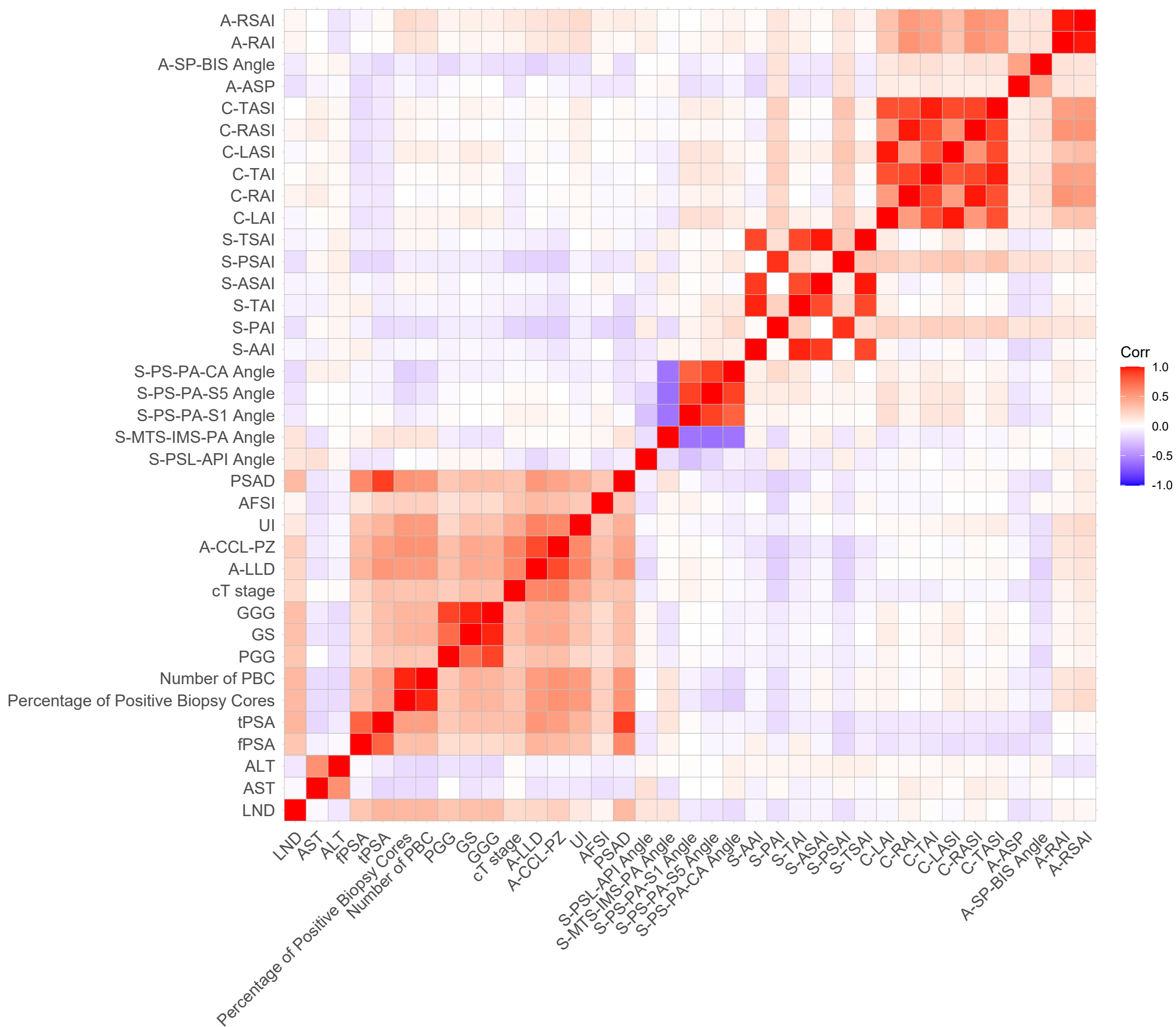

Supplement: Supplementary Figure 2 — Feature elimination via Spearman’s rank correlation analysis. Note: A-RSAI, Axial plane-Right spatial anatomical structure index. A-RAI, Axial plane-Right anatomical structure interval. A-SP-BIS Angle, Axial plane-Inferior margin of symphysis pubis - bilateral ischial spinous angle. A-ASP, Axial plane-The angle of the symphysis pubis. C-TSAI, Coronal plane-Total spatial anatomical structure index. C-RSAI, Coronal plane-Right spatial anatomical structure index. C-LSAI, Coronal plane-Left spatial anatomical structure index. C-TAI, Coronal plane-Total anatomical structure interval. C-RAI, Coronal plane-Right anatomical structure interval. C-LAI, Coronal plane-Left anatomical structure interval. S-TSAI, Sagittal plane-Total spatial anatomical structure index. S-PSAI, Sagittal plane-Posterior spatial anatomical structure index. S-ASAI, Sagittal plane-Anterior spatial anatomical structure index. S-TAI, Sagittal plane-Total anatomical structure interval. S-PAI, Sagittal plane-Posterior anatomical structure interval. S-AAI, Sagittal plane-Anterior anatomical structure interval. S-SP-PA-CA Angle, Sagittal plane-Symphysis pubis-prostate apical-coccyx apical angle. S-SP-PA-S5 Angle, Sagittal plane-Symphysis pubis-prostate apical-S5 angle. S-SP-PA-S1 Angle, Sagittal plane-Symphysis pubis-prostate apical-S1 angle. S-MTSP-IMSPA Angle, Sagittal plane-Angle between the medial tangent of the symphysis pubis, and the line connecting the inferior margin of the symphysis pubis and the prostate apical. S-LASP-API Angle, Sagittal plane-Angle between the long axis diameter of the symphysis pubis and anteroposterior diameter of the pelvic outlet angle. PSAD, Total prostate-specific antigen density. AFSI, Anterior fibromuscular stroma invasion. UI, Urethral invasion. A-CCL-PZ, Axial plane-Cumulative contact length-prostate peripheral zone. A-LLD, Axial plane-Largest lesion diameter. cT stage, Clinical primary tumor stage. GGG, Gleason grade group. GS, Gleason score. PGG, Primary Gleason gra [file Image2.pdf]

# Feature selection by LASSO

A

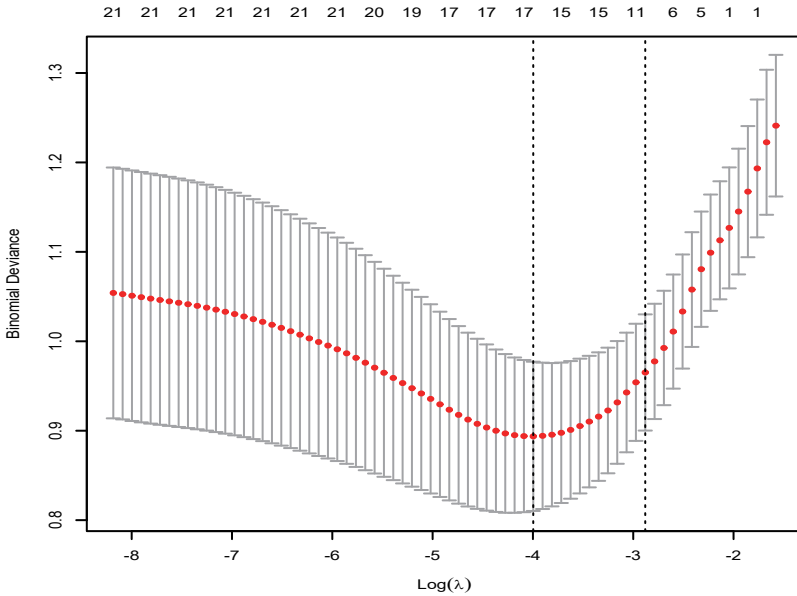

B

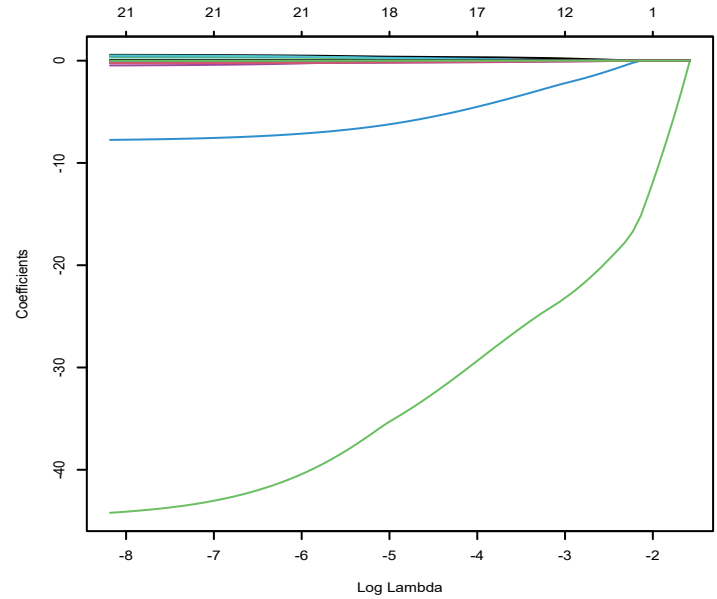

# Feature selection by Boruta

C

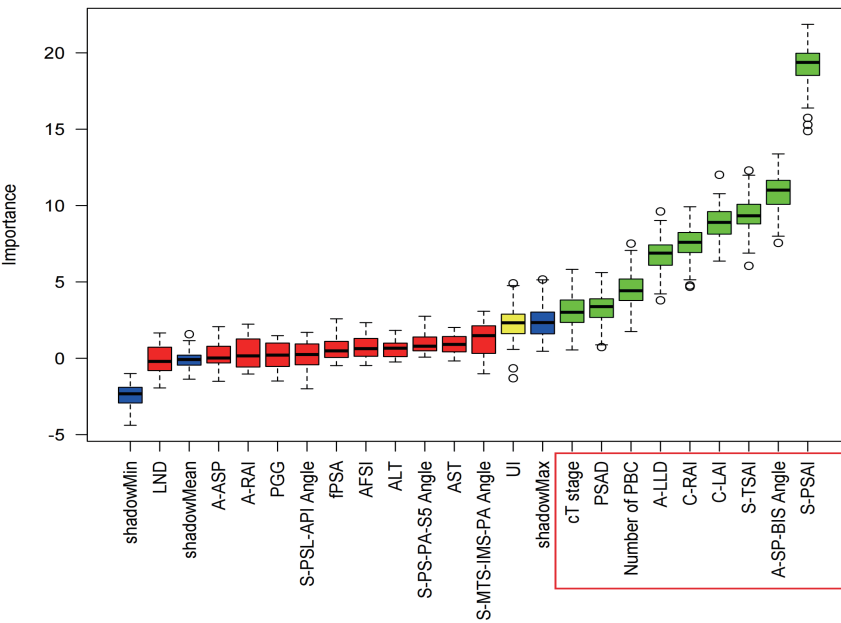

D

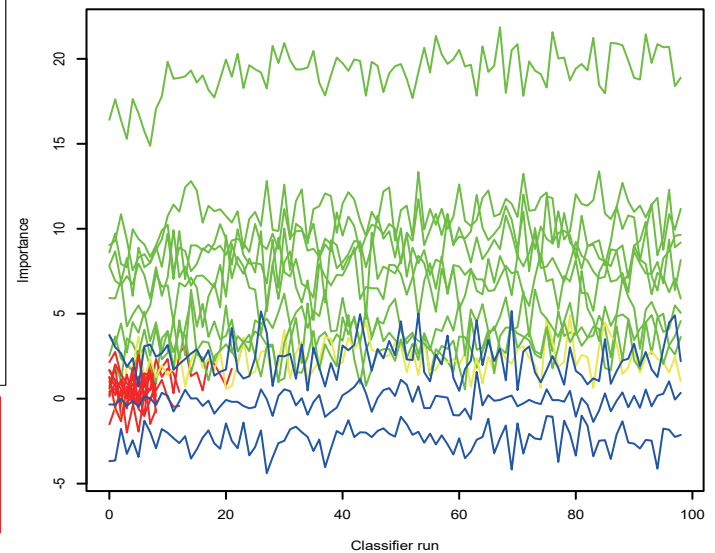

Supplement: Supplementary Figure 3 — Feature screening via LASSO and Boruta algorithms. Note: LND, Lymph node dissection. A-ASP, Axial plane-The angle of the symphysis pubis. A-RAI, Axial plane-Right anatomical structure interval. PGG, Primary Gleason grade. S-LASP-API Angle, Sagittal plane-Angle between the long axis diameter of the symphysis pubis and anteroposterior diameter of the pelvic outlet angle. fPSA, Free prostate-specific antigen. AFSI, Anterior fibromuscular stroma invasion. S-SP-PA-S5 Angle, Sagittal plane-Symphysis pubis-prostate apical-S5 angle. AST, Aspartate aminotransferas. S-MTSP-IMSPA Angle, Sagittal plane-Angle between the medial tangent of the symphysis pubis, and the line connecting the inferior margin of the symphysis pubis and the prostate apical. UI, Urethral invasion. cT stage, Clinical primary tumor stage. PSAD, Total prostate-specific antigen density. Number of PBC, Number of positive biopsy cores. A-LLD, Axial plane-Largest lesion diameter. C-RAI, Coronal plane-Right anatomical structure interval. C-LAI, Coronal plane-Left anatomical structure interval. S-TSAI, Sagittal plane-Total spatial anatomical structure index. A-SP-BIS Angle, Axial plane-Inferior margin of symphysis pubis - bilateral ischial spinous angle. S-PSAI, Sagittal plane-Posterior spatial anatomical structure index. LASSO, the least absolute shrinkage and selection operator. [file Image3.pdf]

**A**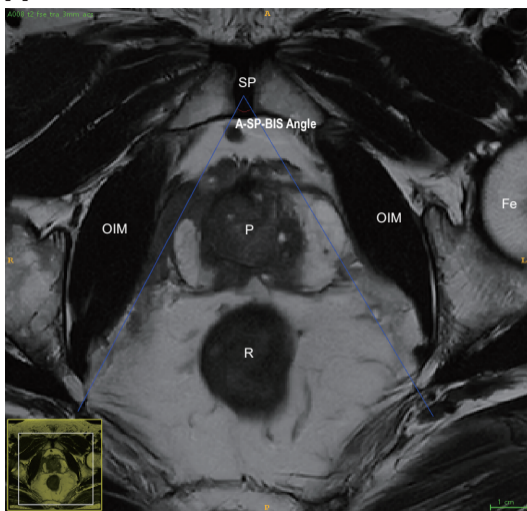

Axial plane

**B**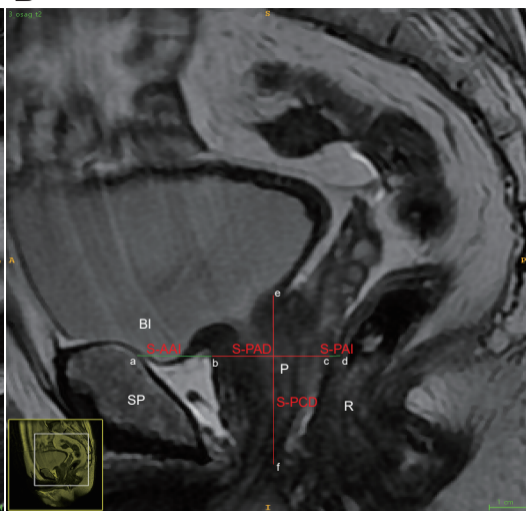

Sagittal plane

**C**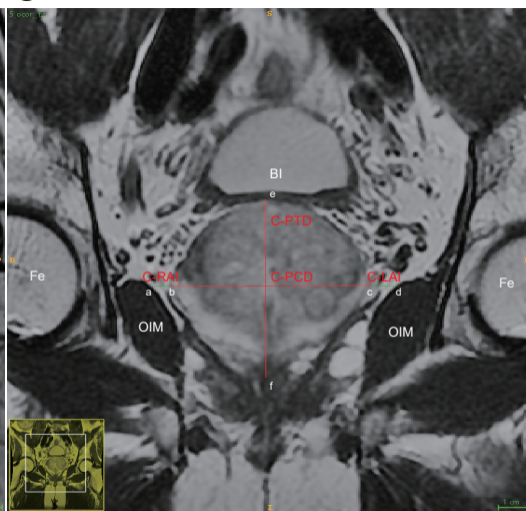

Coronal plane

Supplement: Supplementary Figure 4 — Definitions and images of five key features in MRI measurements. Note: (A) Axial plane of MRI on T2 showing the maximum area of the prostate. A-SP-BIS-Angle was defined as the angle between the lines connecting the inferior margin of the symphysis pubis to the medial aspects of the bilateral ischial spinous processes. (B) Sagittal plane of MRI on T2 showing the maximum area of the prostate. Prostate maximum anteroposterior diameter(PAD) was defined as The maximum anteroposterior diameter at the largest area of the prostate(bc). Prostate maximum craniocaudal diameter(PCD) was defined as The maximum craniocaudal diameter at the largest area of the prostate(ef). Anterior anatomical structure interval diameter(S-AAI) was defined as the horizontal distance between the posterior margin of the symphysis pubis and the anterior margin of the prostate, which was measured at the level of the maximum anteroposterior diameter with the largest area of the prostate (ab). Posterior anatomical structure interval diameter(S-PAI) was defined as the horizontal distance between the posterior margin of the prostate and the anterior margin of the rectum, which was measured at the level of the maximum anteroposterior diameter with the largest area of the prostate (cd). Posterior spatial anatomical structure index(S-PSAI), Calculated based on the formula: (S-RAI) / (S-PAD); Total spatial anatomical structure index(S-TSAI), Calculated based on the formula: ('S-AAI'+'S-PAI') / (S-PAD). (C) Coronal plane of MRI on T2 showing the maximum area of the prostate. Prostate transverse diameter(C-PTD) was defined as the maximum transverse diameter at the largest area of the prostate(bc). Prostate craniocaudal diameter(C-PCD) was defined as the maximum craniocaudal diameter at the largest area of the prostate and the inner edge of the left obturator internus muscle(ef). Left anatomical structure interval(C-LAI) was defined as the horizontal distance between the left edge at the maximum transverse diame [file Image4.pdf]
