# Supplementary material for: Interpretable machine learning models based on multi-dimensional fusion data for predicting positive surgical margins in robot-assisted radical prostatectomy: a retrospective study
Source: Front Oncol. 2025 Oct 3;15:1661695. doi: 10.3389/fonc.2025.1661695 (PMC12531042; doi:10.3389/fonc.2025.1661695)
Supplement: Supplementary file 5 [file DataSheet1.zip › Suppl. Table 1.DOCX]

Supplemental Table 1. Intraobserver and interobserver correlation coefficients (ICCs) of MRI features.

| **Anatomical feature** | **Intraobserver ICCs (95% CI) (Doctor A)** | **Intraobserver ICCs (95% CI) (Doctor B)** | **Interobserver ICCs (95% CI) (Doctor A VS Doctor B)** |
| --- | --- | --- | --- |
| PI-RADS v2: |  |  |  |
| 2 | 0.88 (0.82-0.92) | 0.86 (0.80-0.91) | 0.82 (0.75-0.88) |
| 3 | 0.85 (0.78-0.90) | 0.83 (0.76-0.88) | 0.78 (0.70-0.85) |
| 4 | 0.91 (0.86-0.94) | 0.89 (0.84-0.93) | 0.84 (0.78-0.89) |
| 5 | 0.94 (0.91-0.96) | 0.92 (0.88-0.95) | 0.88 (0.83-0.92) |
| Lymph-node invasion: |  |  |  |
| No | 0.96 (0.93-0.98) | 0.94 (0.91-0.97) | 0.91 (0.87-0.94) |
| Yes | 0.89 (0.84-0.93) | 0.87 (0.81-0.91) | 0.83 (0.77-0.88) |
| Lympho-vascular invasion: |  |  |  |
| No | 0.92 (0.88-0.95) | 0.89 (0.84-0.93) | 0.85 (0.80-0.90) |
| Yes | 0.83 (0.76-0.88) | 0.80 (0.72-0.86) | 0.76 (0.68-0.82) |
| Perineural invasion: |  |  |  |
| No | 0.90 (0.85-0.93) | 0.87 (0.82-0.91) | 0.83 (0.77-0.88) |
| Yes | 0.78 (0.70-0.84) | 0.75 (0.67-0.81) | **0.70** (0.62-0.77) |
| Urethral invasion: |  |  |  |
| No | 0.93 (0.89-0.96) | 0.90 (0.85-0.94) | 0.86 (0.81-0.90) |
| Yes | 0.85 (0.79-0.90) | 0.83 (0.76-0.88) | 0.79 (0.72-0.85) |
| External urethral sphincter invasion: |  |  |  |
| No | 0.92 (0.88-0.95) | 0.90 (0.85-0.94) | 0.85 (0.80-0.90) |
| Yes | 0.82 (0.75-0.87) | 0.79 (0.71-0.85) | 0.75 (0.67-0.81) |
| Seminal vesicle invasion: |  |  |  |
| No | 0.95 (0.92-0.97) | 0.93 (0.89-0.96) | 0.89 (0.85-0.92) |
| Yes | 0.87 (0.81-0.91) | 0.84 (0.78-0.89) | 0.80 (0.73-0.86) |
| Rectal invasion: |  |  |  |
| No | 0.96 (0.93-0.98) | 0.94 (0.91-0.96) | 0.90 (0.86-0.93) |
| Yes | 0.86 (0.80-0.91) | 0.83 (0.76-0.88) | 0.79 (0.72-0.85) |
| Anterior Fibromuscular Stroma invasion: |  |  |  |
| No | 0.93 (0.89-0.96) | 0.91 (0.87-0.94) | 0.87 (0.82-0.91) |
| Yes | 0.84 (0.77-0.89) | 0.81 (0.74-0.86) | 0.76 (0.69-0.82) |
| **cT stage, n(%):** |  |  |  |
| **1** | **0.96 (0.93-0.98)** | **0.94 (0.91-0.97)** | **0.91 (0.87-0.94)** |
| **2** | **0.93 (0.89-0.96)** | **0.91 (0.87-0.94)** | **0.87 (0.82-0.91)** |
| **3** | **0.90 (0.85-0.94)** | **0.88 (0.83-0.92)** | **0.88 (0.83-0.93)** |
| **4** | **0.96 (0.91-0.98)** | **0.95 (0.89-0.98)** | **0.92 (0.85-0.96)** |
| **Axial plane** |  |  |  |
| A-TROIM, (mm): | 0.98 (0.96-0.99) | 0.97 (0.95-0.98) | 0.95 (0.93-0.97) |
| A-TLOIM, (mm): | 0.97 (0.95-0.98) | 0.96 (0.94-0.98) | 0.94 (0.91-0.96) |
| A-DOLAM, (mm): | 0.96 (0.93-0.98) | 0.94 (0.91-0.96) | 0.91 (0.88-0.94) |
| A-DILAM, (mm): | 0.95 (0.92-0.97) | 0.93 (0.89-0.96) | 0.90 (0.86-0.93) |
| A-UW, (mm): | **0.99** (0.98-0.995) | 0.98 (0.96-0.99) | 0.97 (0.95-0.98) |
| A-UWT, (mm): | 0.98 (0.96-0.99) | 0.97 (0.95-0.98) | 0.95 (0.93-0.97) |
| A-TMUT, (mm): | 0.97 (0.95-0.98) | 0.96 (0.93-0.98) | 0.94 (0.91-0.96) |
| A-APMUT, (mm): | 0.96 (0.93-0.98) | 0.94 (0.91-0.96) | 0.92 (0.89-0.94) |
| A-RLP, (mm): | 0.95 (0.92-0.97) | 0.93 (0.90-0.96) | 0.90 (0.86-0.93) |
| A-LLP, (mm): | 0.96 (0.93-0.98) | 0.94 (0.91-0.96) | 0.92 (0.89-0.94) |
| A-LLD, (mm): | 0.97 (0.95-0.98) | 0.95 (0.92-0.97) | 0.93 (0.90-0.95) |
| A-CCL-PZ, (mm): | 0.98 (0.96-0.99) | 0.96 (0.94-0.98) | 0.94 (0.91-0.96) |
| A-OID, (mm): | 0.96 (0.93-0.98) | 0.94 (0.91-0.96) | 0.92 (0.89-0.94) |
| A-AAI, (mm): | 0.95 (0.92-0.97) | 0.93 (0.89-0.96) | 0.90 (0.86-0.93) |
| A-ISD, (mm): | 0.97 (0.95-0.98) | 0.95 (0.92-0.97) | 0.93 (0.90-0.95) |
| A-SW, (mm): | 0.98 (0.96-0.99) | 0.97 (0.95-0.98) | 0.95 (0.93-0.97) |
| A-BFW, (mm): | 0.96 (0.93-0.98) | 0.94 (0.91-0.96) | 0.92 (0.89-0.94) |
| A-ITD, (mm): | 0.95 (0.92-0.97) | 0.93 (0.89-0.96) | 0.90 (0.86-0.93) |
| A-ASP, (°): | 0.97 (0.95-0.98) | 0.95 (0.92-0.97) | 0.93 (0.90-0.95) |
| **A-SP-BIS Angle, (°):** | **0.96 (0.93-0.98)** | **0.94 (0.91-0.96)** | **0.92 (0.89-0.94)** |
| A-PTD, (mm): | 0.95 (0.92-0.97) | 0.93 (0.89-0.96) | 0.90 (0.86-0.93) |
| A-PAD, (mm): | 0.97 (0.95-0.98) | 0.95 (0.92-0.97) | 0.93 (0.90-0.95) |
| A-LAI, (mm): | 0.96 (0.93-0.98) | 0.94 (0.91-0.96) | 0.92 (0.89-0.94) |
| A-RAI, (mm): | 0.95 (0.92-0.97) | 0.93 (0.89-0.96) | 0.90 (0.86-0.93) |
| A-NTL, n(%): | 0.98 (0.96-0.99) | 0.97 (0.95-0.98) | 0.95 (0.93-0.97) |
| 0 | 0.99 (0.98-0.995) | 0.98 (0.96-0.99) | **0.97** (0.95-0.98) |
| 1 | 0.97 (0.95-0.98) | 0.96 (0.93-0.98) | 0.94 (0.91-0.96) |
| 2 | 0.95 (0.92-0.97) | 0.93 (0.89-0.96) | 0.90 (0.86-0.93) |
| ≥3 | 0.93 (0.89-0.96) | 0.91 (0.87-0.94) | 0.88 (0.83-0.92) |
| A-TLI , n(%): | 0.96 (0.93-0.98) | 0.94 (0.91-0.96) | 0.92 (0.89-0.94) |
| No | 0.97 (0.95-0.98) | 0.95 (0.92-0.97) | 0.93 (0.90-0.95) |
| Yes | 0.93 (0.89-0.96) | 0.91 (0.87-0.94) | 0.88 (0.83-0.92) |
| **Sagittal plane** |  |  |  |
| S-PUL, (mm): | 0.98 (0.96-0.99) | 0.97 (0.95-0.98) | 0.95 (0.93-0.97) |
| S-MUL, (mm): | 0.97 (0.95-0.98) | 0.95 (0.92-0.97) | 0.93 (0.90-0.95) |
| S-MUA, (°): | 0.96 (0.93-0.98) | 0.94 (0.91-0.96) | 0.92 (0.89-0.94) |
| S-LASP, (mm): | 0.95 (0.92-0.97) | 0.93 (0.89-0.96) | 0.90 (0.86-0.93) |
| S-API, (mm): | 0.97 (0.95-0.98) | 0.95 (0.92-0.97) | 0.93 (0.90-0.95) |
| S-APM, (mm): | 0.96 (0.93-0.98) | 0.94 (0.91-0.96) | 0.92 (0.89-0.94) |
| S-APO, (mm): | 0.95 (0.92-0.97) | 0.93 (0.89-0.96) | 0.90 (0.86-0.93) |
| S-PD, (mm): | 0.98 (0.96-0.99) | 0.97 (0.95-0.98) | 0.95 (0.93-0.97) |
| S-SD, (mm): | 0.97 (0.95-0.98) | 0.95 (0.92-0.97) | 0.93 (0.90-0.95) |
| S-S1AMCAL, (mm): | 0.96 (0.93-0.98) | 0.94 (0.91-0.96) | 0.92 (0.89-0.94) |
| S-AVPJ, (mm): | 0.95 (0.92-0.97) | 0.93 (0.89-0.96) | 0.90 (0.86-0.93) |
| S-AD, (mm): | 0.97 (0.95-0.98) | 0.95 (0.92-0.97) | 0.93 (0.90-0.95) |
| S-BH, (mm): | 0.96 (0.93-0.98) | 0.94 (0.91-0.96) | 0.92 (0.89-0.94) |
| S-IPPH, (mm): | 0.95 (0.92-0.97) | 0.93 (0.89-0.96) | 0.90 (0.86-0.93) |
| S-UUP, (mm): | 0.98 (0.96-0.99) | 0.97 (0.95-0.98) | 0.95 (0.93-0.97) |
| S-DUP, (mm): | 0.97 (0.95-0.98) | 0.95 (0.92-0.97) | 0.93 (0.90-0.95) |
| S-SA, (°): | 0.96 (0.93-0.98) | 0.94 (0.91-0.96) | 0.92 (0.89-0.94) |
| S-RMA, (°): | 0.95 (0.92-0.97) | 0.93 (0.89-0.96) | 0.90 (0.86-0.93) |
| S-PIA, (°): | 0.97 (0.95-0.98) | 0.95 (0.92-0.97) | 0.93 (0.90-0.95) |
| S-LASP-APO Angle, (°): | 0.96 (0.93-0.98) | 0.94 (0.91-0.96) | 0.92 (0.89-0.94) |
| S-LASP-API Angle, (°): | 0.95 (0.92-0.97) | 0.93 (0.89-0.96) | 0.90 (0.86-0.93) |
| S-LASP-PD Angle, (°): | 0.97 (0.95-0.98) | 0.95 (0.92-0.97) | 0.93 (0.90-0.95) |
| S-APO-API Angle, (°): | 0.96 (0.93-0.98) | 0.94 (0.91-0.96) | 0.92 (0.89-0.94) |
| S-MTSP-IMSPA Angle, (°): | 0.95 (0.92-0.97) | 0.93 (0.89-0.96) | 0.90 (0.86-0.93) |
| S-SP-PA-S1 Angle, (°): | 0.97 (0.95-0.98) | 0.95 (0.92-0.97) | 0.93 (0.90-0.95) |
| S-SP-PA-S5 Angle, (°): | 0.96 (0.93-0.98) | 0.94 (0.91-0.96) | 0.92 (0.89-0.94) |
| S-SP-PA-CA Angle, (°): | 0.95 (0.92-0.97) | 0.93 (0.89-0.96) | 0.90 (0.86-0.93) |
| **S-PAD, (mm):** | **0.98 (0.96-0.99)** | **0.97 (0.95-0.98)** | **0.95 (0.93-0.97)** |
| S-PCD, (mm): | 0.97 (0.95-0.98) | 0.95 (0.92-0.97) | 0.93 (0.90-0.95) |
| **S-AAI, (mm):** | **0.96 (0.93-0.98)** | **0.94 (0.91-0.96)** | **0.92 (0.89-0.94)** |
| **S-PAI, (mm):** | **0.95 (0.92-0.97)** | **0.93 (0.89-0.96)** | **0.90 (0.86-0.93)** |
| **Coronal plane** |  |  |  |
| C-RST, (mm): | 0.98 (0.96-0.99) | 0.97 (0.95-0.98) | 0.95 (0.93-0.97) |
| C-LST, (mm): | 0.97 (0.95-0.98) | 0.95 (0.92-0.97) | 0.93 (0.90-0.95) |
| C-TRLAM, (mm): | 0.96 (0.93-0.98) | 0.94 (0.91-0.96) | 0.92 (0.89-0.94) |
| C-TLLAM, (mm): | 0.95 (0.92-0.97) | 0.93 (0.89-0.96) | 0.90 (0.86-0.93) |
| C-TVPJ, (mm): | 0.97 (0.95-0.98) | 0.95 (0.92-0.97) | 0.93 (0.90-0.95) |
| C-IPPH, (mm): | 0.96 (0.93-0.98) | 0.94 (0.91-0.96) | 0.92 (0.89-0.94) |
| C-TIP, (mm): | 0.95 (0.92-0.97) | 0.93 (0.89-0.96) | 0.90 (0.86-0.93) |
| C-TTP, (mm): | 0.98 (0.96-0.99) | 0.97 (0.95-0.98) | 0.95 (0.93-0.97) |
| C-PTD, (mm): | 0.97 (0.95-0.98) | 0.95 (0.92-0.97) | 0.93 (0.90-0.95) |
| C-PCD, (mm): | 0.96 (0.93-0.98) | 0.94 (0.91-0.96) | 0.92 (0.89-0.94) |
| **C-LAI, (mm):** | **0.95 (0.92-0.97)** | **0.93 (0.89-0.96)** | **0.90 (0.86-0.93)** |
| **C-RAI, (mm):** | **0.97 (0.95-0.98)** | **0.95 (0.92-0.97)** | **0.93 (0.90-0.95)** |

Note: CI, confidence interval; PI-RADS v2, Prostate imaging reporting and data system version 2; cT stage, Clinical primary tumor Stage; MRI measurement abbreviations, names, and definitions were detailed in Supplementary Table 2.

This table presents the Interobserver and intraobserver correlation coefficients (ICCs) for the MRI features identified in the study, reflecting inter- and intraobserver agreement between two independent radiologists (with more than 8 years of experience in prostate cancer diagnosis) during manual measurement on T2WI MRI images.

•ICC values range from 0 to 1, with higher values indicating stronger inter- and intraobserver agreement.

•Interpretation criteria: ICC ≥0.85 indicates excellent agreement; 0.70–0.84 indicates good agreement; 0.50–0.69 indicates moderate agreement; <0.50 indicates poor agreement.

•All five key spatial anatomical features demonstrated excellent interobserver agreement, supporting the reliability of manual measurements in routine practice.

•The controversial cases were reevaluated by a third senior radiologist (with more than 15 years of experience in prostate cancer diagnosis).
