# Supplementary material for: Interpretable machine learning models based on multi-dimensional fusion data for predicting positive surgical margins in robot-assisted radical prostatectomy: a retrospective study
Source: Front Oncol. 2025 Oct 3;15:1661695. doi: 10.3389/fonc.2025.1661695 (PMC12531042; doi:10.3389/fonc.2025.1661695)
Supplement: Supplementary file 5 [file DataSheet1.zip › Suppl. Table 3.DOCX]

Supplemental Table 3. Baseline characteristics of selected patients.

| **Positive surgical margin (PSM)** | **[ALL] N=277** | **[Negative] N=192** | **[Positive] N=85** | P-value |
| --- | --- | --- | --- | --- |
| **Demographic and medical history:** |  |  |  |  |
| Age, median (IQR) (year): | 69.0 [65.0;74.0] | 69.0 [63.0;73.0] | 69.0 [66.0;75.0] | 0.214 |
| Body mass index, median (IQR) (kg/m^2^): | 24.2 [22.4;25.7] | 24.3 [22.4;25.7] | 23.9 [22.6;25.7] | 0.788 |
| Family history of PCa, n(% ) : |  |  |  | 1 |
| No | 276 (99.6%) | 191 (99.5%) | 85 (100%) |  |
| Yes | 1 (0.36%) | 1 (0.52%) | 0 (0.00%) |  |
| Abdominal surgery, n(% ) : |  |  |  | 0.316 |
| No | 208 (75.1%) | 148 (77.1%) | 60 (70.6%) |  |
| Yes | 69 (24.9%) | 44 (22.9%) | 25 (29.4%) |  |
| TURP, Over 1 year, n(% ) : |  |  |  | 0.729 |
| No | 267 (96.4%) | 184 (95.8%) | 83 (97.6%) |  |
| Yes | 10 (3.61%) | 8 (4.17%) | 2 (2.35%) |  |
| Smoking, n(% ) : |  |  |  | 0.28 |
| No | 168 (60.6%) | 121 (63.0%) | 47 (55.3%) |  |
| Yes | 109 (39.4%) | 71 (37.0%) | 38 (44.7%) |  |
| Drinking, n(% ) : |  |  |  | 0.873 |
| No | 186 (67.1%) | 130 (67.7%) | 56 (65.9%) |  |
| Yes | 91 (32.9%) | 62 (32.3%) | 29 (34.1%) |  |
| Hypertension, n(% ) : |  |  |  | 0.154 |
| No | 160 (57.8%) | 105 (54.7%) | 55 (64.7%) |  |
| Yes | 117 (42.2%) | 87 (45.3%) | 30 (35.3%) |  |
| Diabetes, n(% ) : |  |  |  | 0.153 |
| No | 222 (80.1%) | 149 (77.6%) | 73 (85.9%) |  |
| Yes | 55 (19.9%) | 43 (22.4%) | 12 (14.1%) |  |
| Cardiovascular disease, n(% ) : |  |  |  | 0.639 |
| No | 234 (84.5%) | 164 (85.4%) | 70 (82.4%) |  |
| Yes | 43 (15.5%) | 28 (14.6%) | 15 (17.6%) |  |
| **Variables of laboratory, Preoperative:** |  |  |  |  |
| Urinalysis white blood cell, median (IQR) (cell/ul): | 2.00 [1.00;6.00] | 2.00 [1.00;7.00] | 2.00 [1.00;5.00] | 0.62 |
| Platelet, median (IQR) (109/L): | 184 [156;212] | 184 [156;215] | 179 [155;207] | 0.578 |
| Hematocrit, median (IQR) (%): | 42.5 [40.2;44.6] | 42.5 [40.4;44.5] | 42.0 [39.9;44.8] | 0.651 |
| Hemoglobin, median (IQR) (g/L): | 141 [132;148] | 140 [133;148] | 141 [131;148] | 0.505 |
| White blood cell, median (IQR) (109/L): | 5.68 [4.96;6.82] | 5.68 [4.96;6.72] | 5.78 [4.97;6.93] | 0.895 |
| Lymphocyte, median (IQR) (109/L): | 1.61 [1.31;1.97] | 1.60 [1.33;1.91] | 1.67 [1.29;2.03] | 0.71 |
| Monocyte, median (IQR) (109/L): | 0.47 [0.39;0.56] | 0.46 [0.38;0.56] | 0.47 [0.39;0.54] | 0.783 |
| Neutrophil, median (IQR) (109/L): | 3.36 [2.69;4.26] | 3.33 [2.70;4.15] | 3.41 [2.68;4.50] | 0.77 |
| Neutrophil percentage, median (IQR) (%): | 59.1 [53.4;64.1] | 59.1 [53.7;63.8] | 59.2 [53.4;64.4] | 0.79 |
| Neutrophil-to-lymphocyte ratio, median (IQR) : | 2.06 [1.60;2.72] | 2.07 [1.58;2.71] | 2.04 [1.63;2.74] | 0.922 |
| Lymphocyte-to-monocyte ratio, median (IQR) : | 3.50 [2.82;4.32] | 3.47 [2.76;4.31] | 3.63 [2.89;4.32] | 0.684 |
| Platelet-to-lymphocyte ratio, median (IQR) : | 116 [90.1;139] | 119 [89.9;139] | 113 [90.8;144] | 0.613 |
| Monocyte-to-lymphocyte ratio, median (IQR) : | 0.29 [0.23;0.35] | 0.29 [0.23;0.36] | 0.28 [0.23;0.35] | 0.684 |
| Systemic immune-inflammation index, median (IQR) : | 372 [269;546] | 371 [267;553] | 373 [276;519] | 0.94 |
| Fasting blood glucose, median (IQR) (mmol/L): | 5.40 [5.00;6.00] | 5.40 [5.00;6.00] | 5.40 [5.00;5.90] | 0.483 |
| Aspartate aminotransferas, median (IQR) (IU/L): | 20.0 [17.0;24.0] | 21.0 [17.0;25.0] | 19.0 [15.0;24.0] | 0.086 |
| Alanine aminotransferase, median (IQR) (IU/L) : | 19.0 [14.0;25.0] | 20.0 [14.8;25.2] | 16.0 [13.0;24.0] | 0.03 |
| DeRitis ratio, median (IQR) : | 1.05 [0.85;1.29] | 1.02 [0.83;1.29] | 1.08 [0.90;1.31] | 0.423 |
| Blood urea nitrogen, median (IQR) (mmol/L) : | 5.90 [4.90;7.00] | 5.80 [4.77;7.00] | 6.20 [5.10;7.00] | 0.348 |
| Serum creatinine, median (IQR) (umol/L): | 81.0 [74.0;94.0] | 81.0 [72.8;95.0] | 83.0 [74.0;93.0] | 0.909 |
| eGFR, median (IQR) (ml/min/1.73m2): | 88.4 [74.4;98.2] | 89.2 [74.4;98.6] | 87.2 [74.4;95.8] | 0.504 |
| Uric acid, median (IQR) (umol/L): | 350 [302;399] | 350 [300;398] | 353 [312;413] | 0.381 |
| Prothrombin time, median (IQR) (s): | 12.9 [12.3;13.5] | 13.0 [12.3;13.5] | 12.8 [12.4;13.4] | 0.414 |
| Activated partial thromboplastin time, median (IQR) (s): | 35.0 [32.4;37.5] | 35.2 [32.3;38.0] | 34.8 [33.2;36.7] | 0.635 |
| Fibrinogen, median (IQR) (g/L): | 2.87 [2.55;3.23] | 2.88 [2.57;3.23] | 2.86 [2.52;3.25] | 0.654 |
| Thrombin time, median (IQR) (s): | 17.6 [17.0;18.4] | 17.6 [17.0;18.5] | 17.7 [17.0;18.2] | 0.829 |
| International normalized ratio, median (IQR): | 0.99 [0.95;1.04] | 0.99 [0.95;1.04] | 0.97 [0.94;1.03] | 0.067 |
| D-dimer, median (IQR) (mg/L): | 0.34 [0.20;0.71] | 0.34 [0.22;0.71] | 0.33 [0.19;0.66] | 0.6 |
| fPSA, median (IQR) (ng/ml): | 1.46 [0.93;2.54] | 1.33 [0.90;2.21] | 1.79 [1.11;3.32] | 0.001 |
| tPSA, median (IQR) (ng/ml): | 13.6 [9.06;25.0] | 11.7 [8.73;19.0] | 20.2 [12.3;32.3] | <0.001 |
| fPSA/tPSA | 0.10 [0.07;0.14] | 0.10 [0.08;0.14] | 0.10 [0.07;0.14] | 0.36 |
| **Biopsy pathology:** |  |  |  |  |
| Biopsy Methods, n(% ): |  |  |  | 0.697 |
| Conventional | 60 (21.7%) | 39 (20.3%) | 21 (24.7%) |  |
| Systematic biopsy | 157 (56.7%) | 110 (57.3%) | 47 (55.3%) |  |
| MRI-ultrasound fusion-guided targeted biopsy | 60 (21.7%) | 43 (22.4%) | 17 (20.0%) |  |
| Number of biopsy cores, median (IQR): | 12.0 [12.0;12.0] | 12.0 [12.0;12.0] | 12.0 [12.0;13.0] | 0.368 |
| Number of positive biopsy cores, median (IQR): | 5.00 [3.00;7.00] | 4.00 [2.00;7.00] | 6.00 [4.00;9.00] | <0.001 |
| Percentage of positive biopsy cores, median (IQR) (%): | 41.7 [22.2;58.3] | 34.8 [18.2;53.9] | 50.0 [33.3;68.8] | <0.001 |
| Primary Gleason grade, n(% ): |  |  |  | 0.027 |
| 3 | 150 (54.2%) | 113 (58.9%) | 37 (43.5%) |  |
| 4 | 118 (42.6%) | 75 (39.1%) | 43 (50.6%) |  |
| 5 | 9 (3.25%) | 4 (2.08%) | 5 (5.88%) |  |
| Secondary Gleason grade, n(% ): |  |  |  | 0.035 |
| 3 | 139 (50.2%) | 96 (50.0%) | 43 (50.6%) |  |
| 4 | 113 (40.8%) | 84 (43.8%) | 29 (34.1%) |  |
| 5 | 25 (9.03%) | 12 (6.25%) | 13 (15.3%) |  |
| Gleason score, n(% ): |  |  |  | 0.011 |
| 3+3 | 78 (28.2%) | 59 (30.7%) | 19 (22.4%) |  |
| 3+4, 4+3 | 128 (46.2%) | 87 (45.3%) | 41 (48.2%) |  |
| 3+5, 4+4, 5+3 | 43 (15.5%) | 34 (17.7%) | 9 (10.6%) |  |
| 4+5, 5+4 | 25 (9.03%) | 11 (5.73%) | 14 (16.5%) |  |
| 5+5 | 3 (1.08%) | 1 (0.52%) | 2 (2.35%) |  |
| Gleason grade group, n(% ): |  |  |  | 0.004 |
| 1 | 80 (28.9%) | 60 (31.2%) | 20 (23.5%) |  |
| 2 | 68 (24.5%) | 51 (26.6%) | 17 (20.0%) |  |
| 3 | 58 (20.9%) | 35 (18.2%) | 23 (27.1%) |  |
| 4 | 43 (15.5%) | 34 (17.7%) | 9 (10.6%) |  |
| 5 | 28 (10.1%) | 12 (6.25%) | 16 (18.8%) |  |
| **MRI:** |  |  |  |  |
| PI-RADS v2, n(% ): |  |  |  | <0.001 |
| 2 | 27 (9.75%) | 23 (12.0%) | 4 (4.71%) |  |
| 3 | 21 (7.58%) | 14 (7.29%) | 7 (8.24%) |  |
| 4 | 54 (19.5%) | 48 (25.0%) | 6 (7.06%) |  |
| 5 | 175 (63.2%) | 107 (55.7%) | 68 (80.0%) |  |
| Lymph-node invasion: |  |  |  | 0.087 |
| No | 273 (98.6%) | 191 (99.5%) | 82 (96.5%) |  |
| Yes | 4 (1.44%) | 1 (0.52%) | 3 (3.53%) |  |
| Lympho-vascular invasion: |  |  |  | 0.01 |
| No | 265 (95.7%) | 188 (97.9%) | 77 (90.6%) |  |
| Yes | 12 (4.33%) | 4 (2.08%) | 8 (9.41%) |  |
| Perineural invasion: |  |  |  | 0.169 |
| No | 258 (93.1%) | 182 (94.8%) | 76 (89.4%) |  |
| Yes | 19 (6.86%) | 10 (5.21%) | 9 (10.6%) |  |
| Urethral invasion: |  |  |  | 0.002 |
| No | 193 (69.7%) | 145 (75.5%) | 48 (56.5%) |  |
| Yes | 84 (30.3%) | 47 (24.5%) | 37 (43.5%) |  |
| External urethral sphincter invasion: |  |  |  | 0.01 |
| No | 265 (95.7%) | 188 (97.9%) | 77 (90.6%) |  |
| Yes | 12 (4.33%) | 4 (2.08%) | 8 (9.41%) |  |
| Seminal vesicle invasion: |  |  |  | 0.038 |
| No | 266 (96.0%) | 188 (97.9%) | 78 (91.8%) |  |
| Yes | 11 (3.97%) | 4 (2.08%) | 7 (8.24%) |  |
| Rectal invasion: |  |  |  | 0.307 |
| No | 276 (99.6%) | 192 (100%) | 84 (98.8%) |  |
| Yes | 1 (0.36%) | 0 (0.00%) | 1 (1.18%) |  |
| Anterior Fibromuscular Stroma invasion: |  |  |  | 0.003 |
| No | 192 (69.3%) | 144 (75.0%) | 48 (56.5%) |  |
| Yes | 85 (30.7%) | 48 (25.0%) | 37 (43.5%) |  |
| Clinical primary tumor Stage (cT stage), n(%): |  |  |  | <0.001 |
| 1 | 27 (9.75%) | 22 (11.5%) | 5 (5.88%) |  |
| 2 | 168 (60.6%) | 127 (66.1%) | 41 (48.2%) |  |
| 3 | 68 (24.5%) | 39 (20.3%) | 29 (34.1%) |  |
| 4 | 14 (5.05%) | 4 (2.08%) | 10 (11.8%) |  |
| **Axial plane** |  |  |  |  |
| A-TROIM, median (IQR) (mm): | 19.5 [17.5;21.5] | 19.2 [17.4;21.4] | 19.8 [17.7;22.1] | 0.184 |
| A-TLOIM, median (IQR) (mm): | 19.2 [17.2;21.1] | 19.0 [17.1;20.8] | 19.6 [17.3;21.7] | 0.32 |
| A-DOLAM, median (IQR) (mm): | 40.8 [38.3;43.0] | 40.8 [38.8;43.4] | 40.7 [38.2;42.6] | 0.308 |
| A-DILAM, median (IQR) (mm): | 15.2 [14.0;16.6] | 15.3 [14.1;16.5] | 14.9 [13.7;17.0] | 0.705 |
| A-UW, median (IQR) (mm): | 1.28 [1.14;1.41] | 1.28 [1.13;1.41] | 1.28 [1.18;1.48] | 0.561 |
| A-UWT, median (IQR) (mm): | 1.93 [1.72;2.29] | 1.92 [1.69;2.28] | 2.01 [1.79;2.35] | 0.281 |
| A-TMUT, median (IQR) (mm): | 7.69 [6.76;8.63] | 7.82 [6.74;8.62] | 7.61 [6.89;8.63] | 0.825 |
| A-APMUT, median (IQR) (mm): | 7.64 [6.84;8.59] | 7.71 [6.86;8.61] | 7.44 [6.83;8.48] | 0.376 |
| A-RLP, median (IQR) (mm): | 4.93 [3.75;6.62] | 4.97 [3.83;6.44] | 4.92 [3.65;6.93] | 0.957 |
| A-LLP, median (IQR) (mm): | 4.92 [3.78;6.53] | 4.96 [3.79;6.53] | 4.71 [3.75;6.58] | 0.746 |
| A-LLD, median (IQR) (mm): | 18.1 [10.9;27.9] | 16.3 [9.62;24.2] | 24.2 [15.8;35.6] | <0.001 |
| A-CCL-PZ, median (IQR) (mm): | 14.0 [0.00;33.9] | 10.2 [0.00;24.3] | 22.1 [11.5;48.4] | <0.001 |
| A-OID, median (IQR) (mm): | 73.1 [65.4;81.6] | 73.3 [66.3;81.3] | 72.7 [64.4;81.8] | 0.415 |
| A-AAI, median (IQR) (mm): | 9.84 [7.57;13.0] | 9.89 [7.92;13.2] | 9.63 [6.81;12.2] | 0.239 |
| A-ISD, median (IQR) (mm): | 92.0 [88.1;96.2] | 91.8 [88.4;95.6] | 92.6 [86.4;98.2] | 0.675 |
| A-SW, median (IQR) (mm): | 79.4 [68.2;86.3] | 79.5 [69.4;85.0] | 78.7 [66.4;89.3] | 0.704 |
| A-BFW, median (IQR) (mm): | 95.2 [91.1;99.5] | 95.0 [90.8;99.1] | 95.6 [91.5;101] | 0.198 |
| A-ITD, median (IQR) (mm): | 118 [112;125] | 118 [112;124] | 118 [114;125] | 0.583 |
| A-ASP, median (IQR) (°): | 73.3 [68.8;77.5] | 73.7 [69.3;78.0] | 72.3 [67.2;76.7] | 0.099 |
| A-SP-BIS Angle, median (IQR) (°): | 56.4 [52.8;60.1] | 57.1 [53.9;60.6] | 54.4 [51.1;58.6] | <0.001 |
| A-PTD, median (IQR) (mm): | 49.0 [45.6;53.1] | 49.2 [46.2;53.0] | 48.6 [45.0;53.2] | 0.382 |
| A-PAD, median (IQR) (mm): | 38.2 [34.8;42.6] | 38.3 [34.6;42.6] | 37.9 [35.3;41.9] | 0.685 |
| A-LAI, median (IQR) (mm): | 5.24 [3.86;7.61] | 5.39 [4.05;7.64] | 5.12 [3.34;7.05] | 0.079 |
| A-RAI, median (IQR) (mm): | 5.42 [3.63;7.65] | 5.56 [3.88;7.88] | 4.95 [2.87;7.26] | 0.039 |
| A-NTL, n(%): |  |  |  | 0.144 |
| 0 | 40 (14.4%) | 32 (16.7%) | 8 (9.41%) |  |
| 1 | 179 (64.6%) | 117 (60.9%) | 62 (72.9%) |  |
| 2 | 45 (16.2%) | 35 (18.2%) | 10 (11.8%) |  |
| ≥3 | 13 (4.69%) | 8 (4.17%) | 5 (5.88%) |  |
| A-TLI , n(%): |  |  |  | 0.162 |
| No | 40 (14.4%) | 32 (16.7%) | 8 (9.41%) |  |
| Yes | 237 (85.6%) | 160 (83.3%) | 77 (90.6%) |  |
| **Sagittal plane** |  |  |  |  |
| S-PUL, median (IQR) (mm): | 45.0 [41.4;49.6] | 44.8 [41.4;50.8] | 45.7 [42.1;48.8] | 0.576 |
| S-MUL, median (IQR) (mm): | 15.0 [14.0;15.9] | 14.9 [13.9;16.0] | 15.2 [14.2;15.8] | 0.654 |
| S-MUA, median (IQR) (°): | 122 [116;128] | 121 [117;128] | 122 [115;130] | 0.895 |
| S-LASP, median (IQR) (mm): | 40.8 [38.3;43.6] | 40.4 [38.1;42.9] | 41.5 [39.0;44.3] | 0.042 |
| S-API, median (IQR) (mm): | 110 [103;116] | 110 [103;116] | 109 [103;115] | 0.52 |
| S-APM, median (IQR) (mm): | 107 [103;112] | 107 [103;112] | 106 [102;111] | 0.222 |
| S-APO, median (IQR) (mm): | 86.9 [81.4;91.5] | 87.0 [81.6;91.3] | 86.5 [81.3;91.8] | 0.92 |
| S-PD, median (IQR) (mm): | 124 [118;131] | 124 [118;132] | 124 [119;130] | 0.278 |
| S-SD, median (IQR) (mm): | 33.2 [28.6;37.6] | 33.9 [28.5;38.5] | 32.1 [28.6;36.5] | 0.131 |
| S-S1AMCAL, median (IQR) (mm): | 125 [118;133] | 127 [118;133] | 123 [117;132] | 0.092 |
| S-AVPJ, median (IQR) (mm): | 16.0 [12.3;20.4] | 16.1 [12.0;20.3] | 15.6 [12.6;20.4] | 0.853 |
| S-AD, median (IQR) (mm): | 33.4 [30.1;37.3] | 32.8 [29.7;37.3] | 34.0 [30.7;37.6] | 0.196 |
| S-BH, median (IQR) (mm): | 12.4 [6.89;18.1] | 11.6 [6.84;18.1] | 13.0 [8.72;18.4] | 0.264 |
| S-IPPH, median (IQR) (mm): | 0.00 [0.00;5.22] | 0.00 [0.00;5.08] | 1.45 [0.00;5.33] | 0.712 |
| S-UUP, median (IQR) (mm): | 6.55 [2.66;11.1] | 6.22 [2.63;11.1] | 7.82 [2.92;11.9] | 0.226 |
| S-DUP, median (IQR) (mm): | 30.6 [27.5;34.3] | 30.0 [27.3;34.4] | 31.2 [28.9;34.2] | 0.133 |
| S-SA, median (IQR) (°): | 39.1 [35.9;42.4] | 39.4 [36.2;42.9] | 38.1 [35.7;40.6] | 0.049 |
| S-RMA, median (IQR) (°): | 155 [146;163] | 155 [145;163] | 156 [148;165] | 0.365 |
| S-PIA, median (IQR) (°): | 69.1 [66.0;72.7] | 69.0 [66.0;72.7] | 69.2 [66.3;72.4] | 0.674 |
| S-LASP-APO Angle, median (IQR) (°): | 130 [126;135] | 130 [126;135] | 130 [126;134] | 0.683 |
| S-LASP-API Angle, median (IQR) (°): | 101 [95.7;105] | 101 [96.3;106] | 99.1 [94.4;104] | 0.025 |
| S-LASP-PD Angle, median (IQR) (°): | 59.4 [55.9;62.6] | 58.9 [55.7;62.4] | 60.8 [57.2;63.1] | 0.038 |
| S-APO-API Angle, median (IQR) (°): | 51.2 [45.9;56.5] | 51.7 [47.8;56.9] | 49.7 [44.9;54.4] | 0.051 |
| S-MTSP-IMSPA Angle, median (IQR) (°): | 130 [122;137] | 129 [121;136] | 132 [124;140] | 0.033 |
| S-SP-PA-S1 Angle, median (IQR) (°): | 74.8 [67.8;81.3] | 75.2 [68.6;82.4] | 73.9 [67.1;78.9] | 0.141 |
| S-SP-PA-S5 Angle, median (IQR) (°): | 131 [124;140] | 131 [124;141] | 129 [123;138] | 0.072 |
| S-SP-PA-CA Angle, median (IQR) (°): | 148 [139;159] | 150 [140;160] | 145 [138;154] | 0.022 |
| S-PAD, median (IQR) (mm): | 37.1 [33.2;41.2] | 36.5 [32.9;40.8] | 38.6 [33.6;42.5] | 0.073 |
| S-PCD, median (IQR) (mm): | 45.3 [41.2;51.3] | 44.7 [40.8;50.5] | 46.6 [42.7;53.1] | 0.01 |
| S-AAI, median (IQR) (mm): | 13.2 [10.3;16.4] | 13.8 [11.2;16.7] | 11.7 [8.69;14.8] | 0.001 |
| S-PAI, median (IQR) (mm): | 2.51 [1.89;3.35] | 2.89 [2.23;3.77] | 1.79 [1.41;2.21] | <0.001 |
| **Coronal plane** |  |  |  |  |
| C-RST, median (IQR) (mm): | 7.31 [6.15;8.81] | 7.44 [6.15;8.91] | 7.11 [6.12;8.05] | 0.13 |
| C-LST, median (IQR) (mm): | 7.39 [6.14;8.75] | 7.41 [6.09;8.89] | 7.31 [6.23;8.49] | 0.549 |
| C-TRLAM, median (IQR) (mm): | 4.84 [4.03;5.42] | 4.81 [3.95;5.48] | 4.86 [4.11;5.36] | 0.71 |
| C-TLLAM, median (IQR) (mm): | 4.74 [4.16;5.52] | 4.73 [4.07;5.49] | 4.84 [4.25;5.52] | 0.503 |
| C-TVPJ, median (IQR) (mm): | 20.1 [15.3;25.2] | 19.7 [15.3;25.0] | 21.6 [15.5;25.9] | 0.352 |
| C-IPPH, median (IQR) (mm): | 2.79 [0.00;6.24] | 0.64 [0.00;6.17] | 4.15 [0.00;6.82] | 0.013 |
| C-TIP, median (IQR) (mm): | 111 [106;116] | 112 [106;116] | 110 [106;116] | 0.73 |
| C-TTP, median (IQR) (mm): | 104 [100.0;108] | 104 [100.0;107] | 104 [99.8;108] | 0.895 |
| C-PTD, median (IQR) (mm): | 49.5 [46.1;53.2] | 49.3 [46.2;53.0] | 49.8 [45.5;54.0] | 0.643 |
| C-PCD, median (IQR) (mm): | 41.8 [37.6;47.7] | 41.7 [37.5;47.5] | 42.5 [38.2;48.4] | 0.383 |
| C-LAI, median (IQR) (mm): | 4.32 [2.94;6.10] | 4.64 [3.75;6.63] | 3.14 [2.29;4.52] | <0.001 |
| C-RAI, median (IQR) (mm): | 4.62 [3.17;6.36] | 4.97 [3.49;6.68] | 3.51 [2.44;5.40] | <0.001 |
| **Calculated values** |  |  |  |  |
| A-TLAM, median (IQR) (mm): | 12.6 [11.6;13.9] | 12.7 [11.7;14.0] | 12.5 [11.2;13.7] | 0.138 |
| A-PMI, median (IQR) (mm): | 23.1 [17.8;31.2] | 23.2 [18.0;31.1] | 23.1 [17.0;31.4] | 0.421 |
| A-RR, median (IQR): | 0.78 [0.72;0.85] | 0.78 [0.72;0.85] | 0.78 [0.73;0.86] | 0.309 |
| A-TAI, median (IQR) (mm): | 10.7 [7.83;14.4] | 10.7 [8.21;15.0] | 10.8 [6.87;14.1] | 0.061 |
| A-LSAI, median (IQR): | 0.11 [0.07;0.16] | 0.11 [0.08;0.16] | 0.11 [0.06;0.15] | 0.089 |
| A-RSAI, median (IQR): | 0.11 [0.07;0.15] | 0.11 [0.08;0.16] | 0.10 [0.06;0.15] | 0.066 |
| A-TSAI, median (IQR): | 0.22 [0.15;0.31] | 0.22 [0.17;0.31] | 0.21 [0.13;0.30] | 0.066 |
| S-RR, median (IQR): | 0.80 [0.74;0.87] | 0.80 [0.74;0.87] | 0.80 [0.75;0.85] | 0.768 |
| S-TAI, median (IQR) (mm): | 16.0 [12.8;19.3] | 17.3 [14.1;20.3] | 13.3 [10.8;16.8] | <0.001 |
| S-ASAI, median (IQR): | 0.36 [0.27;0.46] | 0.38 [0.29;0.49] | 0.30 [0.24;0.41] | <0.001 |
| S-PSAI, median (IQR): | 0.07 [0.05;0.09] | 0.08 [0.06;0.10] | 0.05 [0.04;0.06] | <0.001 |
| S-TSAI, median (IQR): | 0.43 [0.33;0.53] | 0.46 [0.37;0.57] | 0.37 [0.28;0.46] | <0.001 |
| C-RR, median (IQR): | 0.85 [0.77;0.92] | 0.85 [0.77;0.92] | 0.87 [0.78;0.93] | 0.31 |
| C-TAI, median (IQR) (mm): | 9.19 [6.78;12.2] | 9.79 [7.72;12.8] | 7.06 [4.76;9.58] | <0.001 |
| C-LSAI, median (IQR): | 0.09 [0.06;0.13] | 0.09 [0.07;0.14] | 0.06 [0.04;0.10] | <0.001 |
| C-RSAI, median (IQR): | 0.09 [0.06;0.12] | 0.10 [0.07;0.13] | 0.07 [0.05;0.11] | <0.001 |
| C-TSAI, median (IQR): | 0.18 [0.13;0.25] | 0.20 [0.15;0.27] | 0.14 [0.10;0.19] | <0.001 |
| A-CSAMU, median (IQR) (mm2): | 46.0 [36.6;57.1] | 46.0 [36.7;57.2] | 45.9 [34.8;54.4] | 0.65 |
| MUV, median (IQR) (mm3): | 678 [541;839] | 673 [545;845] | 695 [508;821] | 0.612 |
| PV, median (IQR) (ml): | 44.2 [36.0;57.8] | 42.1 [35.6;57.1] | 46.9 [36.6;58.7] | 0.326 |
| PSAD, median (IQR) (ng/ml/ml): | 0.33 [0.19;0.59] | 0.29 [0.18;0.48] | 0.44 [0.29;0.67] | <0.001 |
| PCI, median (IQR) (mm): | 80.9 [76.6;85.4] | 80.5 [76.6;84.9] | 82.0 [76.4;85.7] | 0.351 |
| PV/PCI, median (IQR), median (IQR) (mm2): | 0.54 [0.44;0.72] | 0.53 [0.44;0.72] | 0.55 [0.44;0.70] | 0.491 |
| S-BH/AD, median (IQR) (mm): | 0.38 [0.21;0.57] | 0.36 [0.19;0.56] | 0.40 [0.23;0.62] | 0.568 |
| BWI, median (IQR): | 2.86 [2.55;3.14] | 2.87 [2.55;3.19] | 2.86 [2.55;3.06] | 0.544 |
| SWI, median (IQR) : | 2.30 [2.01;2.63] | 2.33 [2.02;2.66] | 2.25 [2.00;2.62] | 0.332 |
| PDI , median (IQR) (mm): | 2.77 [2.49;3.08] | 2.78 [2.49;3.11] | 2.69 [2.49;2.98] | 0.339 |
| PDI/PV, median (IQR) (/ml): | 0.06 [0.05;0.08] | 0.06 [0.05;0.08] | 0.06 [0.04;0.08] | 0.269 |
| **Robot-assisted radical prostatectomy (RARP) :** |  |  |  |  |
| TI-MRI-PB, (IQR) (day): | 3.00 [1.00;7.00] | 3.00 [1.75;7.25] | 3.00 [1.00;5.00] | 0.167 |
| TI-PB-S, median (IQR) (day): | 14.0 [10.0;20.0] | 14.0 [10.0;21.0] | 13.0 [7.00;16.0] | 0.021 |
| Inpatient ward, n(% ): |  |  |  | 0.479 |
| 1 | 138 (49.8%) | 91 (47.4%) | 47 (55.3%) |  |
| 2 | 113 (40.8%) | 82 (42.7%) | 31 (36.5%) |  |
| 3 | 26 (9.39%) | 19 (9.90%) | 7 (8.24%) |  |
| Surgeons, n(% ): |  |  |  | 0.785 |
| A | 89 (32.1%) | 64 (33.3%) | 25 (29.4%) |  |
| B | 46 (16.6%) | 29 (15.1%) | 17 (20.0%) |  |
| C | 45 (16.2%) | 32 (16.7%) | 13 (15.3%) |  |
| D | 40 (14.4%) | 26 (13.5%) | 14 (16.5%) |  |
| Others | 57 (20.6%) | 41 (21.4%) | 16 (18.8%) |  |
| Concomitant surgical procedures, n(% ): |  |  |  | 1 |
| No | 272 (98.2%) | 188 (97.9%) | 84 (98.8%) |  |
| Yes | 5 (1.81%) | 4 (2.08%) | 1 (1.18%) |  |
| Number of laparoscopic incisions, n(% ): |  |  |  | 0.005 |
| 5 | 128 (46.2%) | 99 (51.6%) | 29 (34.1%) |  |
| 6 | 145 (52.3%) | 92 (47.9%) | 53 (62.4%) |  |
| Others | 4 (1.44%) | 1 (0.52%) | 3 (3.53%) |  |
| Surgical approach, n(% ): |  |  |  | 0.265 |
| Intraperitoneal | 201 (72.6%) | 135 (70.3%) | 66 (77.6%) |  |
| Extraperitoneal | 76 (27.4%) | 57 (29.7%) | 19 (22.4%) |  |
| Lymph node dissection, n(% ): |  |  |  | 0.021 |
| No | 209 (75.5%) | 153 (79.7%) | 56 (65.9%) |  |
| Yes | 68 (24.5%) | 39 (20.3%) | 29 (34.1%) |  |

Note: TURP, Transurethral resection of the prostate; Systemic immune-inflammation index (SII) = Neutrophil* Platelet/Lymphocyte; DeRitis ratio=Aspartate aminotransferas/Alanine aminotransferase; eGFR, Estimated glomerular filtration rate; fPSA, Free prostate-specific antigen; tPSA, Total prostate-specific antigen; PI-RADS v2, Prostate imaging reporting and data system version 2; TI-MRI-PB, The time interval of MRI to prostate biopsy; TI-PB-S, The time interval of prostate biopsy to surgery; MRI measurement abbreviations, names, and definitions were detailed in Supplementary Table 2.
