# Supplementary material for: Interpretable machine learning models based on multi-dimensional fusion data for predicting positive surgical margins in robot-assisted radical prostatectomy: a retrospective study
Source: Front Oncol. 2025 Oct 3;15:1661695. doi: 10.3389/fonc.2025.1661695 (PMC12531042; doi:10.3389/fonc.2025.1661695)
Supplement: Supplementary file 5 [file DataSheet1.zip › Suppl. Table 4.DOCX]

Supplemental Table 4. Univariable logistic regression results.

| **Name** | **Desc** | **OR (univariable)** |
| --- | --- | --- |
| **DemograPhic and medical history:** |  |  |
| Age (year): |  | 1.04 (0.99-1.09, *P*=0.110) |
| Body mass index (kg/m^2^): |  | 1.02 (0.91-1.15, *P*=0.708) |
| Abdominal surgery : | No |  |
|  | Yes | 1.72 (0.86-3.44, *P*=0.126) |
| Smoking: | No |  |
|  | Yes | 1.21 (0.65-2.26, *P*=0.551) |
| Drinking: | No |  |
|  | Yes | 0.83 (0.42-1.62, *P*=0.583) |
| Hypertension: | No |  |
|  | Yes | 0.85 (0.45-1.58, *P*=0.602) |
| Diabetes: | No |  |
|  | Yes | 0.54 (0.23-1.25, *P*=0.150) |
| Cardiovascular disease: | No |  |
|  | Yes | 1.42 (0.64-3.11, *P*=0.387) |
| **Variables of laboratory, PreoPerative:** |  |  |
| Urinalysis white blood cell (cell/ul): |  | 1.00 (0.98-1.01, *P*=0.668) |
| Platelet (109/L): |  | 1.00 (0.99-1.01, *P*=0.870) |
| Hematocrit (%): |  | 0.97 (0.89-1.06, *P*=0.517) |
| Hemoglobin (g/L): |  | 0.99 (0.97-1.01, *P*=0.418) |
| White blood cell (109/L): |  | 1.05 (0.86-1.28, *P*=0.610) |
| LymPhocyte (109/L): |  | 0.90 (0.53-1.51, *P*=0.680) |
| Monocyte (109/L): |  | 0.40 (0.04-3.82, *P*=0.425) |
| NeutroPhil (109/L): |  | 1.10 (0.86-1.40, *P*=0.467) |
| NeutroPhil Percentage (%): |  | 1.02 (0.98-1.06, *P*=0.303) |
| Neutrophil-to-lymphocyte ratio : |  | 1.13 (0.91-1.40, *P*=0.265) |
| Lymphocyte-to-monocyte ratio: |  | 1.02 (0.84-1.24, *P*=0.821) |
| Platelet-to-lymphocyte ratio: |  | 1.00 (1.00-1.01, *P*=0.481) |
| Monocyte-to-lymphocyte ratio: |  | 0.88 (0.05-14.89, *P*=0.929) |
| Systemic immune-inflammation index: |  | 1.00 (1.00-1.00, *P*=0.285) |
| Fasting blood glucose (mmol/L): |  | 0.89 (0.71-1.12, *P*=0.312) |
| Aspartate aminotransferas (IU/L): |  | 0.95 (0.90-1.00, *P*=0.035) |
| Alanine aminotransferase (IU/L) : |  | 0.96 (0.92-0.99, *P*=0.026) |
| DeRitis ratio: |  | 1.44 (0.78-2.65, *P*=0.247) |
| Blood urea nitrogen (mmol/L) : |  | 1.05 (0.86-1.28, *P*=0.645) |
| Serum creatinine (umol/L): |  | 1.00 (0.98-1.02, *P*=0.863) |
| eGFR (ml/min/1.73m2): |  | 1.00 (0.98-1.02, *P*=0.921) |
| Uric acid (umol/L): |  | 1.00 (1.00-1.01, *P*=0.299) |
| Prothrombin time (s): |  | 0.91 (0.66-1.26, *P*=0.587) |
| Activated partial thromboplastin time (s): |  | 0.98 (0.91-1.05, *P*=0.575) |
| Fibrinogen (g/L): |  | 1.10 (0.67-1.83, *P*=0.701) |
| Thrombin time (s): |  | 0.89 (0.70-1.13, *P*=0.344) |
| International normalized ratio: |  | 0.12 (0.00-8.25, *P*=0.325) |
| D-dimer (mg/L): |  | 1.29 (0.86-1.94, *P*=0.222) |
| fPSA (ng/ml): |  | 1.19 (1.04-1.36, *P*=0.012) |
| tPSA (ng/ml): |  | 1.02 (1.00-1.03, *P*=0.014) |
| fPSA/tPSA |  | 0.30 (0.00-101.52, *P*=0.682) |
| **BioPsy Pathology:** |  |  |
| BioPsy Methods: | Conventional |  |
|  | Systematic bioPsy | 0.79 (0.38-1.65, *P*=0.538) |
|  | MRI-ultrasound fusion-guided targeted bioPsy | 0.75 (0.29-1.92, *P*=0.542) |
|  |  | 1.02 (1.01-1.03, *P*=0.002) |
| Number of biopsy cores: |  | 1.01 (0.88-1.17, *P*=0.854) |
| Number of positive biopsy cores: |  | 1.17 (1.06-1.29, *P*=0.002) |
| Percentage of positive biopsy cores (%): |  |  |
| Primary Gleason grade: | 3 |  |
|  | 4 | 1.41 (0.75-2.66, *P*=0.287) |
|  | 5 | 11.69 (1.25-109.40, *P*=0.031) |
| Secondary Gleason grade: | 3 |  |
|  | 4 | 0.79 (0.40-1.55, *P*=0.495) |
|  | 5 | 3.45 (1.20-9.94, *P*=0.022) |
| Gleason score: | 3+3 |  |
|  | 3+4, 4+3 | 1.26 (0.59-2.67, *P*=0.556) |
|  | 3+5, 4+4, 5+3 | 0.59 (0.19-1.82, *P*=0.356) |
|  | 4+5, 5+4 | 5.86 (1.71-20.10, *P*=0.005) |
|  | 5+5 | 5.86 (0.49-69.66, *P*=0.162) |
| Gleason grade group: | 1 |  |
|  | 2 | 1.00 (0.41-2.45, *P*=0.993) |
|  | 3 | 1.57 (0.66-3.75, *P*=0.312) |
|  | 4 | 0.59 (0.19-1.82, *P*=0.356) |
|  | 5 | 5.86 (1.85-18.55, *P*=0.003) |
| **MRI:** |  |  |
| PI-RADS v2: | 2 |  |
|  | 3 | 1.09 (0.20-5.87, *P*=0.919) |
|  | 4 | 0.39 (0.08-1.94, *P*=0.249) |
|  | 5 | 2.49 (0.79-7.90, *P*=0.120) |
| Urethral invasion: | No |  |
|  | Yes | 2.23 (1.17-4.25, *P*=0.014) |
| Anterior Fibromuscular Stroma invasion: | No |  |
|  | Yes | 2.12 (1.09-4.09, *P*=0.026) |
| Clinical primary tumor Stage (cT stage): | 1 |  |
|  | 2 | 1.66 (0.45-6.11, *P*=0.447) |
|  | 3 | 4.31 (1.10-16.79, *P*=0.035) |
|  | 4 | 10.67 (1.67-68.18, *P*=0.012) |
|  |  |  |
| **Axial Plane** |  |  |
| A-TROIM (mm): |  | 0.98 (0.88-1.09, *P*=0.715) |
| A-TLOIM (mm): |  | 0.97 (0.87-1.09, *P*=0.639) |
| A-DOLAM (mm): |  | 0.99 (0.92-1.05, *P*=0.681) |
| A-DILAM (mm): |  | 1.04 (0.97-1.11, *P*=0.256) |
| A-UW (mm): |  | 0.89 (0.64-1.24, *P*=0.486) |
| A-UWT (mm): |  | 0.84 (0.42-1.69, *P*=0.622) |
| A-TMUT (mm): |  | 1.04 (0.83-1.30, *P*=0.732) |
| A-APMUT (mm): |  | 0.92 (0.73-1.17, *P*=0.507) |
| A-RLP (mm): |  | 1.02 (0.87-1.20, *P*=0.768) |
| A-LLP (mm): |  | 0.99 (0.85-1.16, *P*=0.938) |
| A-LLD (mm): |  | 1.04 (1.02-1.07, *P*=0.001) |
| A-CCL-PZ (mm): |  | 1.02 (1.01-1.03, *P*=0.002) |
| A-OID (mm): |  | 0.99 (0.96-1.02, *P*=0.379) |
| A-AAI (mm): |  | 1.02 (0.97-1.08, *P*=0.466) |
| A-ISD (mm): |  | 1.01 (0.96-1.05, *P*=0.746) |
| A-SW (mm): |  | 1.01 (0.98-1.03, *P*=0.679) |
| A-BFW (mm): |  | 1.04 (0.99-1.08, *P*=0.135) |
| A-ITD (mm): |  | 1.00 (0.97-1.03, *P*=0.955) |
| A-ASP (°): |  | 0.95 (0.91-1.00, *P*=0.069) |
| A-SP-BIS Angle (°): |  | 0.87 (0.82-0.94, *P*<0.001) |
| A-PTD (mm): |  | 1.00 (0.95-1.05, *P*=0.940) |
| A-PAD (mm): |  | 1.00 (0.95-1.05, *P*=0.894) |
| A-LAI (mm): |  | 0.95 (0.86-1.05, *P*=0.349) |
| A-RAI (mm): |  | 0.91 (0.82-1.00, *P*=0.057) |
| A-NTL: | 0 |  |
|  | 1 | 2.12 (0.75-6.01, *P*=0.156) |
|  | 2 | 1.72 (0.50-5.95, *P*=0.390) |
|  | ≥3 | 2.64 (0.47-14.89, *P*=0.271) |
| A-TLI: | No |  |
|  | Yes | 2.06 (0.74-5.75, *P*=0.166) |
| **Sagittal Plane** |  |  |
| S-PUL (mm): |  | 1.02 (0.98-1.06, *P*=0.434) |
| S-MUL (mm): |  | 1.06 (0.87-1.30, *P*=0.549) |
| S-MUA (°): |  | 1.00 (0.97-1.03, *P*=0.946) |
| S-LASP (mm): |  | 1.05 (0.96-1.14, *P*=0.280) |
| S-API (mm): |  | 0.98 (0.94-1.02, *P*=0.245) |
| S-APM (mm): |  | 0.99 (0.95-1.04, *P*=0.702) |
| S-APO (mm): |  | 1.01 (0.97-1.05, *P*=0.580) |
| S-PD (mm): |  | 0.97 (0.94-1.01, *P*=0.112) |
| S-SD (mm): |  | 0.98 (0.94-1.03, *P*=0.497) |
| S-S1AMCAL (mm): |  | 0.99 (0.97-1.02, *P*=0.465) |
| S-AVPJ (mm): |  | 1.00 (0.95-1.05, *P*=0.887) |
| S-AD (mm): |  | 1.02 (0.97-1.08, *P*=0.426) |
| S-BH (mm): |  | 1.00 (0.97-1.03, *P*=0.999) |
| S-IPPH (mm): |  | 1.02 (0.96-1.07, *P*=0.546) |
| S-UUP (mm): |  | 1.02 (0.97-1.07, *P*=0.418) |
| S-DUP (mm): |  | 1.04 (0.98-1.10, *P*=0.236) |
| S-SA (°): |  | 0.96 (0.91-1.01, *P*=0.146) |
| S-RMA (°): |  | 1.02 (0.99-1.04, *P*=0.151) |
| S-PIA (°): |  | 0.97 (0.92-1.03, *P*=0.306) |
| S-LASP-APO Angle (°): |  | 1.02 (0.98-1.07, *P*=0.334) |
| S-LASP-API Angle (°): |  | 0.96 (0.92-1.01, *P*=0.099) |
| S-LASP-PD Angle (°): |  | 1.03 (0.98-1.10, *P*=0.267) |
| S-APO-API Angle (°): |  | 0.98 (0.95-1.02, *P*=0.335) |
| S-MTSP-IMSPA Angle (°): |  | 1.03 (1.00-1.06, *P*=0.025) |
| S-SP-PA-S1 Angle (°): |  | 0.97 (0.94-1.00, *P*=0.032) |
| S-SP-PA-S5 Angle (°): |  | 0.97 (0.95-0.99, *P*=0.014) |
| S-SP-PA-CA Angle (°): |  | 0.97 (0.95-0.99, *P*=0.007) |
| S-PAD (mm): |  | 1.04 (0.99-1.09, p=0.169) |
| S-PCD (mm): |  | 1.02 (0.98-1.06, p=0.249) |
| S-AAI (mm): |  | 0.92 (0.87-0.98, *P*=0.012) |
| S-PAI (mm): |  | 0.30 (0.19-0.47, *P*<0.001) |
| **Coronal Plane** |  |  |
| C-RST (mm): |  | 0.92 (0.79-1.07, *P*=0.285) |
| C-LST (mm): |  | 0.99 (0.86-1.14, *P*=0.870) |
| C-TRLAM (mm): |  | 0.91 (0.70-1.19, *P*=0.506) |
| C-TLLAM (mm): |  | 1.00 (0.76-1.31, *P*=0.983) |
| C-TVPJ (mm): |  | 1.01 (0.97-1.05, *P*=0.654) |
| C-IPPH (mm): |  | 1.03 (0.97-1.09, *P*=0.334) |
| C-TIP (mm): |  | 1.00 (0.96-1.04, *P*=0.865) |
| C-TTP (mm): |  | 1.00 (0.95-1.06, *P*=0.904) |
| C-PTD (mm): |  | 1.03 (0.97-1.09, *P*=0.314) |
| C-PCD (mm): |  | 1.01 (0.98-1.05, *P*=0.480) |
| C-LAI (mm): |  | 0.74 (0.62-0.88, *P*<0.001) |
| C-RAI (mm): |  | 0.72 (0.61-0.86, *P*<0.001) |
| **Calculated value** |  |  |
| A-TSAI: |  | 0.00 (0.00-0.01, *P*<0.001) |
| A-PMI (mm): |  | 0.99 (0.96-1.02, *P*=0.532) |
| A-RR: |  | 3.76 (0.14-98.02, *P*=0.426) |
| A-TAI (mm): |  | 0.96 (0.91-1.01, *P*=0.120) |
| A-LSAI: |  | 0.13 (0.00-9.48, *P*=0.355) |
| A-RSAI : |  | 0.02 (0.00-1.95, *P*=0.096) |
| A-TSAI: |  | 0.19 (0.02-1.92, *P*=0.160) |
| S-RR : |  | 2.93 (0.08-110.37, *P*=0.562) |
| S-TAI (mm): |  | 0.89 (0.83-0.95, *P*<0.001) |
| S-ASAI : |  | 0.03 (0.00-0.28, *P*=0.002) |
| S-PSAI : |  | 0.00 (0.00-0.00, *P*<0.001) |
| S-TSAI : |  | 0.01 (0.00-0.07, *P*<0.001) |
| C-TLAM (mm): |  | 0.95 (0.87-1.04, *P*=0.287) |
| C-RR: |  | 5.45 (0.25-121.33, *P*=0.284) |
| C-TAI (mm): |  | 0.81 (0.73-0.90, *P*<0.001) |
| C-LSAI: |  | 0.00 (0.00-0.00, *P*<0.001) |
| C-RSAI: |  | 0.00 (0.00-0.00, *P*<0.001) |
| A-CSAMU (mm2): |  | 1.00 (0.98-1.02, *P*=0.742) |
| MUV (mm3): |  | 1.00 (1.00-1.00, *P*=0.901) |
| PV (ml): |  | 1.00 (0.99-1.02, *P*=0.879) |
| PSAD (ng/ml/ml): |  | 1.66 (1.00-2.77, *P*=0.051) |
| PCI (mm): |  | 1.02 (0.97-1.07, *P*=0.499) |
| PV/PCI (mm2): |  | 0.94 (0.29-3.06, *P*=0.917) |
| S-BH/AD (mm): |  | 0.72 (0.26-2.00, *P*=0.532) |
| BWI : |  | 1.12 (0.80-1.56, *P*=0.513) |
| SWI : |  | 1.11 (0.81-1.50, *P*=0.518) |
| PDI (mm): |  | 1.08 (0.79-1.48, *P*=0.638) |
| PDI/PV (/ml): |  | 0.26 (0.00-12275.61, *P*=0.806) |
| **Robot-assisted radical Prostatectomy (RARP):** |  |  |
| TI-MRI-PB (day): |  | 0.98 (0.94-1.02, *P*=0.384) |
| TI-PB-S (day): |  | 0.97 (0.93-1.01, *P*=0.123) |
| InPatient ward: | 1 |  |
|  | 2 | 0.81 (0.42-1.55, *P*=0.522) |
|  | 3 | 1.18 (0.40-3.50, *P*=0.763) |
| Surgeons: | A |  |
|  | B | 1.49 (0.60-3.72, *P*=0.389) |
|  | C | 0.93 (0.37-2.35, *P*=0.875) |
|  | D | 0.90 (0.33-2.48, *P*=0.843) |
|  | Others | 1.41 (0.58-3.39, *P*=0.448) |
| Number of laParoscoPic incisions : | 5 |  |
|  | 6 | 1.62 (0.85-3.06, *P*=0.140) |
|  | Others | 6.29 (0.54-72.85, *P*=0.141) |
| Surgical approach: | IntraPeritoneal |  |
|  | ExtraPeritoneal | 0.62 (0.31-1.24, *P*=0.174) |
| Lymph node dissection : | No |  |
|  | Yes | 2.05 (1.05-3.99, *P*=0.034) |

Note: Systemic immune-inflammation index (SII)=Neutrophil*Platelet/Lymphocyte; DeRitis ratio=Aspartate aminotransferas/Alanine aminotransferase; eGFR, Estimated glomerular filtration rate; fPSA, Free prostate-specific antigen; tPSA, Total prostate-specific antigen; PI-RADS v2, Prostate imaging reporting and data system version 2; TI-MRI-PB, The time interval of MRI to prostate biopsy; TI-PB-S, The time interval of prostate biopsy to surgery; MRI measurement abbreviations, names, and definitions were detailed in Supplementary Table 2.
