# Supplementary material for: Interpretable machine learning models based on multi-dimensional fusion data for predicting positive surgical margins in robot-assisted radical prostatectomy: a retrospective study
Source: Front Oncol. 2025 Oct 3;15:1661695. doi: 10.3389/fonc.2025.1661695 (PMC12531042; doi:10.3389/fonc.2025.1661695)
Supplement: Supplementary file 5 [file DataSheet1.zip › Suppl. Table 2. MRI measurement names and definitions. (new).docx]

Supplemental Table 1. MRI measurement names and definitions.

| Item | Feature | Abbreviation | Definition |
| --- | --- | --- | --- |
| Axial plane | Thickness of right obturator internus muscle | A-TROIM | The maximum thickness of the right obturator internus muscle was measured on any plane. |
|  | Thickness of left obturator internus muscle | A-TLOIM | The maximum thickness of the left obturator internus muscle was measured on any plane. |
|  | Distance of outer of the levator ani muscle | A-DOLAM | The maximum distance between the outer edges of the levator ani muscles bilaterally was measured at the apical plane of the prostate. |
|  | Distance of inner of the levator ani muscle | A-DILAM | The narrowest distance between the inner edges of the levator ani muscles bilaterally was measured at the apical plane of the prostate. |
|  | Urethral width | A-UW | The maximum transverse diameter of the prostatic urethra was measured in any plane. |
|  | Urethral wall thickness | A-UWT | The widest thickness of the urethral wall was measured at the plane of measuring urethral width. |
|  | Transverse membranous urethral thickness | A-TMUT | The maximum transverse thickness of the membranous urethra was measured at the penile root level. |
|  | Anterior and posterior membranous urethral thickness | A-APMUT | The maximum longitudinal thickness of the membranous urethra was measured at the level of the penile root. |
|  | Right levator prostate thickness | A-RLP | The maximum thickness of the right levator prostate was measured in any plane. |
|  | Left levator prostate thickness | A-LLP | The maximum thickness of the left levator prostate was measured in any plane. |
|  | Largest lesion diameter | A-LLD | The largest lesion diameter of the tumor. |
|  | Cumulative contact length at the prostate peripheral zone | A-CCL-PZ | The cumulative contact length of tumor lesions in the prostate peripheral zone was measured at the maximum area of the tumor. |
|  | Obturator internus diameter | A-OID | The diameter between the inner edge of the obturator internus muscle (the tangent to the lower edge of the prostate) was measured at the maximum area of the prostate. |
|  | Anterior anatomical structure interval diameter | A-AAI | The anteroposterior diameter of the symphysis pubis to the prostate anterior margin was measured at the maximum area of the prostate. |
|  | Interspinous distance | A-ISD | The narrowest distance between the tips of the ischial spines. |
|  | Soft-tissue width | A-SW | The diameter between the inner edge of the obturator internus muscle (the tangent to the lower edge of the prostate) was measured at the mid-femoral head level. |
|  | Bony femoral width | A-BFW | The bony width of the pelvis at the mid-femoral head level. |
|  | Inter tuberous distance | A-ITD | The widest distance between the ischial tuberosities. |
|  |  |  |  |
| Axial plane | The angle of the symphysis pubis | A-ASP | The angle between the lines connecting the inferior margin of the symphysis pubis and the medial aspects of the bilateral femoral bones. |
|  | Inferior margin of symphysis pubis - bilateral ischial spinous Angle | A-SP-BIS-Angle | The angle between the lines connecting the inferior margin of the symphysis pubis to the medial aspects of the bilateral ischial spinous processes. |
|  | Prostate transverse diameter | A-PTD | The maximum transverse diameter at the largest area of the prostate. |
|  | Prostate anteroposterior diameter | A-PAD | The maximum anteroposterior diameter at the largest area of the prostate. |
|  | Left anatomical structure interval | A-LAI | The horizontal distance between the left edge of the maximum transverse diameter of the prostate and the inner edge of the left obturator medial muscle. |
|  | Right anatomical structure interval | A-RAI | The horizontal distance between the right edge at the maximum transverse diameter of the prostate and the inner edge of the right obturator medial muscle. |
| Sagittal plane | Prostatic urethral length | S-PUL | The anatomical length from the internal urethral orifice through the prostate to the urethral of the prostate apical. |
|  | Membranous urethral length | S-MUL | The length from the top of the prostate to the bottom of the bulbar urethra was measured. |
|  | Membranous urethral angle | S-MUA | The angle was obtained between the membranous urethral and crossing a horizontal line under the pubis. |
|  | The long axis diameter of the symphysis pubis | S-LASP | Measured on the sagittal midplane. |
|  | Anteroposterior diameter of the pelvic inlet | S-API | Diameter from the sacral promontory to the most superior aspect of the symphysis pubis. |
|  | Anteroposterior diameter of the pelvic midplane | S-APM | Diameter from the inferior margin of the symphysis pubis to the sacrococcygeal junction. |
|  | Anteroposterior diameter of the pelvic outlet | S-APO | Length from the inferior aspect of the symphysis pubis to the tip of the coccyx was measured. |
|  | Pelvic depth | S-PD | Diameter from the sacral promontory to the most inferior point of the symphysis pubis. |
|  | Sacrococcygeal diameter | S-SD | Diameter from the sacral promontory to the tip of the coccyx. |
|  | The craniocaudal diameter inferior the S5 to the coccyx apical | S-CS5CA | Diameter from the inferior aspect of the S5 to the coccyx apical. |
|  | Anteroposterior diameter of the vesicoprostatic junction | S-AVPJ | The anteroposterior diameter of the bladder and prostate junction was measured at the sagittal plane at the largest area of the prostate. |
|  | Apical depth | S-AD | The vertical diameter from the prostatic apical to the most superior aspect horizontal line of the symphysis pubis. |
|  | Bottom height | S-BH | The vertical diameter from the prostatic bottom to the most superior aspect horizontal line of the symphysis pubis. |
|  | Intravesical prostatic protrusion height | S-IPPH | The vertical diameter from the highest point of the prostatic bottom to the vesicoprostatic line. |
| Sagittal plane | Upper urethral projection | S-UUP | The vertical diameter from the internal urethral orifice to the most superior aspect horizontal line of the symphysis pubis. |
|  | Distal urethral projection | S-DUP | The vertical diameter from the urethral of the prostate is apical to the most superior aspect horizontal line of the symphysis pubis. |
|  | Symphysis angle | S-SA | The angle between the long axis of the symphysis pubis and the horizontal plane on the mid-sagittal plane. |
|  | Rectal Mesorectal Angle | S-RMA | The angle between the line connecting the anterior margin of S5 and the coccygeal apical, and the anterior tangential line of S5. |
|  | Pelvic inclination angle | S-PIA | The angle between the SD and the APO. |
|  | S-LASP-APO Angle | S-LASP-APO Angle | The angle between the LASP and the APO. |
|  | S-LASP-API Angle | S-LASP-API Angle | The angle between the LASP and the API. |
|  | S-LASP-PD Angle | S-LASP-PD Angle | The angle between the LASP and the PD. |
|  | S-APO-API Angle | S-APO-API Angle | The angle between the APO and the API. |
|  | S-MTSP-IMSPA Angle | S-MTSP-IMSPA Angle | The Angle between the medial tangent of the symphysis pubis and the line connecting the inferior margin of the symphysis pubis and the prostate apical. |
|  | Symphysis pubis-prostate apical-S1 angle | S-SP-PA-S1 Angle | The angle between the line connecting the inferior margin of the symphysis pubis and prostate apical, and the line connecting the prostate apical and sacral promontory. |
|  | Symphysis pubis-prostate apical-S5 angle | S-SP-PA-S5 Angle | The angle between the line connecting the inferior margin of the symphysis pubis and prostate apical, and the line connecting the prostate apical and inferior aspect of the S5. |
|  | Symphysis pubis-prostate apical-coccyx apical angle | S-SP-PA-CA Angle | The angle between the line connecting the inferior margin of the symphysis pubis and prostate apical, and the line connecting the prostate apical and the coccyx apical. |
|  | Prostate maximum anteroposterior diameter | S-PAD | The maximum anteroposterior diameter at the largest area of the prostate. |
|  | Prostate maximum craniocaudal diameter | S-PCD | The maximum craniocaudal diameter at the largest area of the prostate. |
|  | Anterior anatomical structure interval diameter | S-AAI | The horizontal distance between the posterior margin of the symphysis pubis and the anterior margin of the prostate was measured at the level of the maximum anteroposterior diameter with the largest area of the prostate. |
|  | Posterior anatomical structure interval diameter | S-PAI | The horizontal distance between the posterior margin of the prostate and the anterior margin of the rectum, which was measured at the level of the maximum anteroposterior diameter with the largest area of the prostate. |
|  |  |  |  |
| Coronal plane | Right anal sphincter thickness | C-RST | The maximum thickness of the right anal sphincter was measured in any plane. |
|  | Left anal sphincter thickness | C-LST | The maximum thickness of the left anal sphincter was measured in any plane. |
|  | Thickness of right levator ani muscle | C-TRLAM | The measured maximum thickness of the right levator ani muscle was measured in any plane. |
|  | Thickness of left levator ani muscle | C-TLLAM | The measured maximum thickness of the left levator ani muscle was measured in any plane. |
|  | Transverse diameter of the vesicoprostatic junction | C-TVPJ | The anteroposterior diameter of the bladder and prostate junction was measured at the coronal plane at the largest area of the prostate. |
|  | Intravesical prostatic protrusion height | C-IPPH | The vertical diameter from the highest point of the prostatic bottom to the vesicoprostatic line. |
|  | Diameter of the transverse inlet pelvis | C-TIP | The diameter of the transverse inlet pelvis at the level superior to the femoral heads was measured in the coronal plane at the largest area of the femoral heads. |
|  | Diameter of transverse true pelvis | C-TTP | The diameter of the transverse true pelvis at the femoral midline level was measured at the coronal plane at the largest area of the femoral. |
|  | Prostate transverse diameter | C-PTD | The maximum transverse diameter at the largest area of the prostate. |
|  | Prostate craniocaudal diameter | C-PCD | The maximum craniocaudal diameter at the largest area of the prostate. |
|  | Left anatomical structure interval | C-LAI | The horizontal distance between the left edge at the maximum transverse diameter of the prostate and the inner edge of the left obturator internus muscle. |
|  | Right anatomical structure interval | C-RAI | The horizontal distance between the right edge at the maximum transverse diameter of the prostate and the inner edge of the right obturator internus muscle. |
| Calculated value | Thickness of the levator ani muscle | A-TLAM | Calculated using the formula: [(A-DOLAM) - (A-DILAM)]/2 |
|  | Prostate-muscle index | A-PMI | (A-OID)-(A-PTD) |
|  | Roundness ratio | A-RR | Defined as a ratio of the radii of the small circle to the large circle, where the circles have diameters corresponding to A-PAD and A-PTD, respectively. |
|  | Total anatomical structure interval | A-TAI | Calculated based on the formula: (A-LAI)+(A-RAI) |
|  | Left spatial anatomical structure index | A-LSAI | Calculated based on the formula: (A-LAI)/(A-PTD) |
|  | Right spatial anatomical structure index | A-RSAI | Calculated based on the formula: (A-RAI)/(A-PTD) |
|  | Total spatial anatomical structure index | A-TSAI | Calculated based on the formula: (A-TAI)/(A-PTD) |
|  | Roundness ratio | S-RR | Defined as a ratio of the radii of the small circle to the large circle, where the circles have diameters corresponding to S-PAD and S-PCD, respectively. |
| Calculated value | Total anatomical structure interval diameter | S-TAI | Calculated based on the formula: (S-AAI)+(S-PAI) |
|  | Anterior spatial anatomical structure index | S-ASAI | Calculated based on the formula: (S-AAI) / (S-PAD) |
|  | Posterior spatial anatomical structure index | S-PSAI | Calculated based on the formula: (S-RAI) / (S-PAD) |
|  | Total spatial anatomical structure index | S-TSAI | Calculated based on the formula: (S-TAI) / (S-PAD) |
|  | Roundness ratio | C-RR | Defined as a ratio of the radii of the small circle to the large circle, which circles with diameters of C-PCD and C-PTD, respectively. |
|  | Total anatomical structure interval | C-TAI | (C-LAI) + (C-RAI) |
|  | Left spatial anatomical structure index | C-LSAI | (C-LAI) / (C-PTD) |
|  | Right spatial anatomical structure index | C-RSAI | (C-RAI) / (C-PTD) |
|  | Total spatial anatomical structure index | C-TSAI | (C-TAI) / (C-PTD) |
|  | The cross-sectional surface area of the membranous urethra | A-CSAMU | Calculated using the formula: (A-TMUT / 2) × (A-APMUT / 2) × π |
|  | Membranous urethral volume | MUV | Calculated using the following formula: (A-CSAMU) × (S-MUL) |
|  | Prostate volume | PV | Calculated using the following formula: (A-PTD) × (A-PTD) × (S-PCD) × π/6 |
|  | PSA density | PSAD | Calculated using the following formula: tPSA/PV |
|  | Pelvic cavity index | PCI | Calculated by the following formula: (S-API)×(A-ISD)/(S-PD) |
|  | PV-to-PCI ratio | PV/PCI | Calculated by the following formula: PV / PCI |
|  | S-BH-to-AD ratio | S-BH/S-AD | Calculated by the following formula: (S-BH) / (S-AD) |
|  | Bony width index | BWI | Calculated by the following formula: (A-BFW) / (S-AD) |
|  | Soft tissue width index | SWI | Calculated by the following formula: (A-SW) / (S-AD) |
|  | Pelvic dimension index | PDI | Calculated by the following formula: (A-ISD)/ (S-AD) |
|  | PDI to prostate volume ratio | PDI/PV | Calculated by the following formula: PDI/PV |
